# Exploring the potential of MXenes in advanced energy, environmental, and biomedical applications

**DOI:** 10.1039/d5ra04498g

**Published:** 2025-11-18

**Authors:** Enas M. Ahmed, Ahmed Shawki Ali, Eman M. Hieba, Zeinab S. Shaban, Marwa Saeed Fathy, Alaa M. Amer, Abdelrahman M. Ishmael, Ahmed Bakr, Huda R. M. Rashdan, Amir Elzwawy

**Affiliations:** a Egyptian Petroleum Research Institute (EPRI) 11727 Nasr City Cairo Egypt; b Department of Energy and Petroleum Engineering, University of Wyoming Laramie WY 82071 USA; c Chemistry and Entomology Department, Faculty of Science, Cairo University Giza 12613 Egypt; d Faculty of Applied Medical Science, Misr University for Science and Technology (MUST) Giza Egypt; e Biophysics Group, Physics Department, Faculty of Science, Ain Shams University Cairo 11566 Egypt; f Materials Science and Nanotechnology Department, Faculty of Postgraduate Studies for Advanced Sciences (PSAS), Beni-Suef University Beni-Suef 62511 Egypt; g Department of Chemistry, School of Science and Engineering, The American University in Cairo Cairo 11835 Egypt; h Nanomaterials Science and its Applications Program, Faculty of Science, Benha University Benha 13518 Egypt; i Spectroscopy Department, Physics Research Institute, National Research Centre (NRC) 33 El Bohouth St., Dokki Giza 12622 Egypt; j Chemistry of Natural and Microbial Products Department, Pharmaceutical and Drug Industries Research Institute, National Research Centre (NRC) 33 El Buhouth St., Dokki Giza 12622 Egypt; k Refractories, Ceramics, and Building materials Department, Advanced Materials Technology and Mineral Resources Research Institute, National Research Centre (NRC) 33 El Bohouth St., Dokki Giza 12622 Egypt elzwawy1@gmail.com aa.elzwawy@nrc.sci.eg Amir.Elzwawy@gu.edu.eg; l Physics Department, Faculty of Science, Galala University New Galala City Suez 43511 Egypt

## Abstract

MXenes, a rapidly expanding family of 2D transition metal carbides, nitrides, and carbonitrides, have emerged as a ground-breaking class of materials thanks to their specifications as customizable surface chemistry, and electrical conductivity. Since their emergence, MXenes have demonstrated their adaptability in a numerous applications, from electronics and biomedicine to energy conversion and storage, environmental clean up, and water purification. This article provides a thorough and critical review of the state-of-the-art in MXene research, covering recent synthesis studies (from traditional HF etching to more environmentally friendly and bottom-up approaches) and the constantly evolving understanding of how MXene structure influences properties. Their usefulness for energy storage devices is highlighted, as their electrochemical characteristics make them excellent candidates for next-generation lithium/sodium-ion batteries and supercapacitors. We proceed to discuss their potential in electrocatalysis, water purification, sensing and biomedical engineering and comment on the correlation between surface functionality and architecture and the corresponding performance in a given application. By bridging recent experimental evidence with theoretical foundations, the article summarizes existing knowledge and highlights topical challenges to advance MXene research in the future. The review concludes a vision of the near future on scalable production and surface modification and interdisciplinary integration towards enabling MXenes in high-performance and multifunctional applications.

## Abstract 2

MXenes, a rapidly expanding family of 2D transition metal carbides, nitrides, and carbonitrides, have emerged as a ground-breaking class of materials thanks to their specifications as customizable surface chemistry, and electrical conductivity.

## Graphical abstract

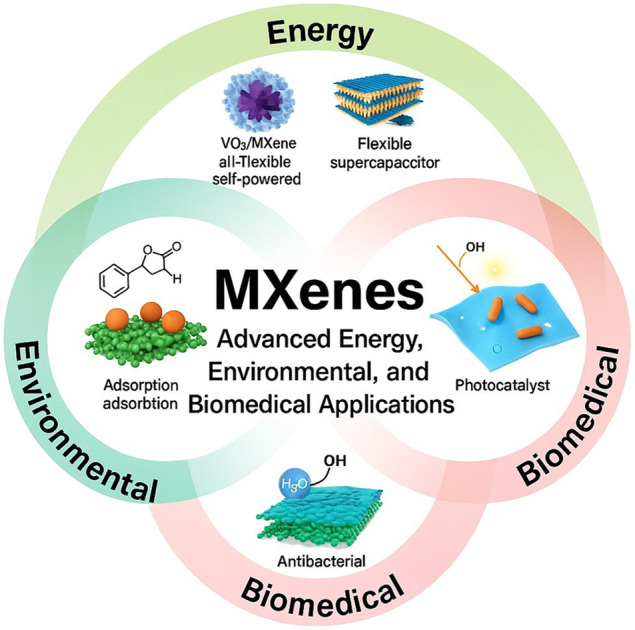

## 1. Introduction

The most recent members of the growing family of two-dimensional (2D) materials include transition metal carbides, nitrides, and carbonitrides.^1^ With the formula M_*n*+1_AX_*n*_T_*x*_ (*n* = 1, 2, or 3; T_*x*_ = –F, –O, or –OH), where M is an early transition metal, A is a group 13 or group 14 element, and X is carbon or nitrogen, MXenes are mostly created *via* wet-chemical exfoliation of precursor MAX phases.^2^ The A layers are eliminated during the process of selective etching, leaving behind delaminated MXene sheets with etchant-added surface terminations. Recent reviews comprehensively map the etching landscape (HF; fluoride-salt + HCl; molten-salt such as SnF_2_; alkaline hydrothermal; and electrochemical routes) and relate synthesis choices to structure and electrochemical performance.^3–5^ For Ti_3_C_2_ specifically, a focused review details how HF concentration/time and bifluoride etchants (NaHF_2_, KHF_2_, NH_4_HF_2_) tune interlayer spacing and surface terminations, linking etch conditions to morphology and electrical behavior.^6^

The number of atomic layers (*n*), surface functional groups (T_*x*_), and M and X elements can all be changed to affect the final structure of MXene.^7^ Owing to these adjustable characteristics, MXenes are being used in a wide range of industries, including composites, water purification, CO_2_ capture, energy storage and conversion, sensing, and catalysis platforms.^8–10^

Concurrently, data-driven catalysis is emerging: ML models trained on DFT datasets now predict adsorption energies on diverse MXene compositions/terminations, accelerating screening for reactions like the water–gas shift.^11^

**Fig. 1:**
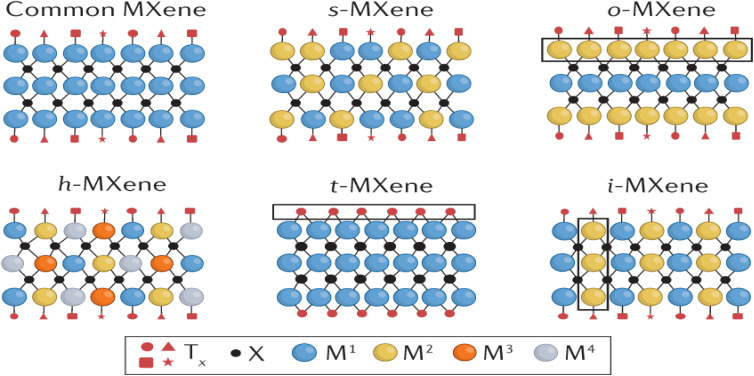
Schematic illustration of the six main structural categories of MXenes, highlighting their compositional and functional diversity within the broader class of 2D materials showing the six key types of MXenes (adapted with permission from ref. 22 Copyright 2024, Elsevier).

There are six main structural types of MXenes as depicted in Fig. 1:^12^ random solid-solution MXenes (s-MXene) like Ti_2−*y*_V_*y*_CT_*x*_,^13^ out-of-plane ordered MXenes (o-MXene) like Mo_2_Ti_2_C_3_T_*x*_,^14^ in-plane ordered MXenes (i-MXene) like (Mo_2/3_Y_1/3_)_2_CT_*x*_,^15^ uniform surface terminations (t-MXene) like Ti_3_C_2_Cl_2_ (ref. 16) and high-entropy MXenes (h-MXene), having multiple elements, *e.g.*, TiVNbMoC_3_T_*x*_^17^ (see Fig. 1). The structural variety allows MXenes to possess tailored properties like high conductivity, remarkable flexibility, large surface area, and good hydrophilicity. The high reactivity of terminally exposed groups also leaves much scope for functionalization and thus MXenes are extremely versatile to adopt to specific purpose. Their exceptional electrochemical properties have brought MXenes to the mainstream in energy-reliant technologies, whereas their biocompatibility and photothermal properties make their area of interest extend to biomedical applications.^18–20^ For example, Si–MXene composites with polydopamine-derived carbon coatings deliver improved stability and conductivity as lithium-ion battery anodes.^3^ For electrochemical systems specifically, simultaneous *in situ* Raman and FTIR tracking of Ti_3_C_2_T_*x*_ and Ti_3_C_2_Cl_2_ under cycling links surface terminations and confined water dynamics directly to charge-storage behavior in H_2_SO_4_, LiCl, and KOH electrolytes.^21^

**Fig. 2:**
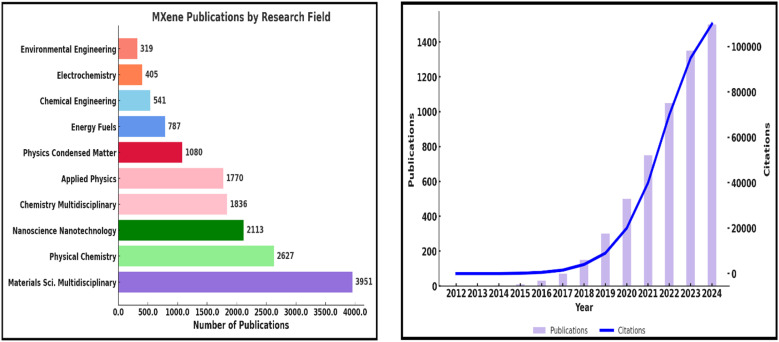
Left: Distribution of MXene-related publications across various research fields, indicating a dominant presence in materials science, chemistry, and nanotechnology. Right: Annual trend in the number of publications and citations related to MXenes from 2012 to 2024, highlighting exponential growth in both research output and impact (WOS database).

This review discusses recent advancements in MXene synthesis methods with a focus on how different conditions and methods impact structural and functional properties. For context, prior comprehensive overviews link MAX-to-MXene protocols and device-level metrics in supercapacitors.^4^ The influence of performance-determining parameters—like conductivity, structure, and hydrophilicity—on MXene behavior in magnetic, optical, mechanical, and electronic dimensions is also analyzed. *In situ* vibrospectroscopy provides these ‘structure–property’ links in real time, resolving potential-dependent shifts in termination modes and O–H signatures of MXene-confined water during ion intercalation.^21^ Moreover, the work links synthesis advances to their practical implementation in renewable energy, environmental remediation, and beyond.^23^ A final section discusses bibliometric trends and market insights relevant to MXene technology.^24^ As illustrated in Fig. 2, a Web of Science search using the keyword “MXenes” yielded 7504 documents. Since their introduction in 2011, research output has surged steadily. MXenes, defined by the formula M_*n*+1_X_*n*_T_*x*_ (where M is a transition metal like Ti, Mo, Zr, or W; X is C or N; and T_*x*_ includes Cl, F, OH, or O), have become a major subclass within 2D materials research^25^ (see Fig. 2). They possess a set of properties—optical, electrical, chemical, and mechanical—that underlie many emerging applications. Recent *in situ* interface studies further clarify how surface chemistry governs these responses during operation.^21^ High surface area, strong chemical stability, hydrophilicity, thermal conductivity, and environmental benefits are all attributes of MXenes.^26^ MXenes are most well-known worldwide for their ability to store energy, but they have also demonstrated significant promise in the realms of healthcare and the environment. These involve applications in photocatalytic and electrocatalytic water splitting, CO_2_ reduction, pollutant sensing, and water purification.^27–31^ For example, ZnO–MXene adsorbents deliver high removal of priority metals while meeting WHO limits, with ML models (RF, SVM) accurately predicting performance under varying pH, dose, and temperature.^32^ They also promote high conductivity to support integration as a component of conductive inks and interconnects as well as to serve as a current collector. This metallic conductivity also permits on-fabric Joule heating, useful for regenerable MXene air-filters.^33^ MXenes' interaction with electromagnetic waves, in a frequency range from terahertz to gigahertz, is the basis of their presence in communication and shielding applications.^34–36^ Recent perspective work highlights MXene fibers and textiles as high-performance, flexible platforms for EMI shielding, sensing, and energy storage, summarizing >1500 fiber-related publications and reporting state-of-the-art conductivities and device metrics.^37^

In environmental engineering, MXenes have surpassed conventional materials in treating contaminated surface and groundwater, industrial and municipal wastewater, and even in desalination efforts.^26,38,39^ MXene-based composites can remove a range of contaminants through adsorption and faradaic capacitive deionization (CDI).^40^ Recent ZnO–MXene nanocomposites achieved 97% Cr, 97% Pb, 96% As, and 91% Cd removal, with Freundlich isotherms and pseudo-second-order kinetics, underscoring MXenes' efficacy for heavy-metal remediation.^32^ This approach mitigates the concentration polarization seen in traditional CDI electrodes and offers a low-energy solution for desalinating brackish water while substantially cutting energy consumption.^41–43^

While previous landmark reviews have significantly advanced the field Li *et al.* (2022, *Nat. Rev. Chem.*)^12^ offered a broad account of MXene synthesis and properties; and Gogotsi *et al.* (2023, *Adv. Mater.*)^44^ integrated emerging biomedical and environmental dimensions these contributions primarily emphasized either chemical classification or application specific demonstrations. In contrast, the present review introduces a unifying quantitative framework linking synthesis conditions, structural evolution, and cross-domain functionality. It consolidates HF-based, HF-free, and bottom-up fabrication strategies within a benchmarking matrix that relates yield, surface chemistry, electrical performance, and safety indices, thus enabling direct comparison across different synthesis families. Furthermore, by including developments up to mid-2025 and emphasizing industrial scalability and biocompatibility, this review advances a comprehensive perspective that bridges laboratory innovation with real-world translation.

Distinct from previous comprehensive reviews, the present work adopts an integrative “synthesis–structure–property–application–scalability” framework that unifies diverse MXene research threads under a coherent conceptual map. While prior overviews have catalogued advances in either synthesis chemistry or application-specific performance, our review explicitly correlates synthetic parameters such as etchant type, termination chemistry, and interlayer control with electrochemical, catalytic, and biocompatibility outcomes. This approach not only synthesizes knowledge across energy, environmental, and biomedical domains but also reveals cross-domain design rules (*e.g.*, surface functionalization balance between –O/–OH terminations enhancing both charge transport and biological tolerance). Furthermore, by integrating recent data-driven and industrial studies published through mid-2025, the review highlights evolving directions toward large-scale, green, and multifunctional MXene technologies.

## 2. Most commonly used techniques for synthesis of MXenes

**Fig. 3:**
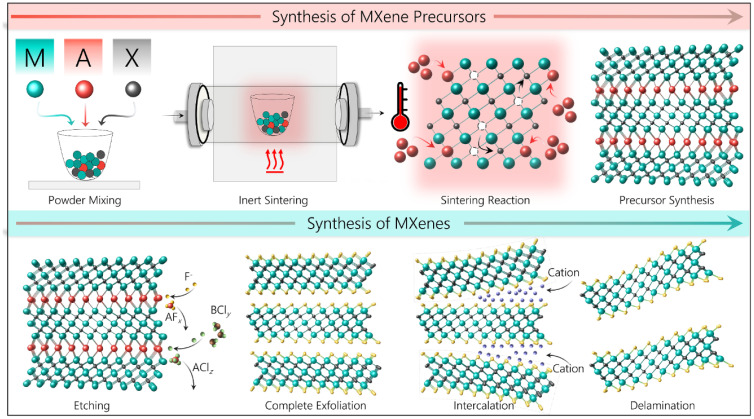
The synthesis of different MXene precursors and MXenes (reproduced with permission from ref. 48 Copyright 2023, Elsevier).

The process and circumstances of their synthesis have an impact on the quality of MXenes, which determines their potential or utility. The synthesis circumstances and settings can be changed to change the desired properties of MXenes because they are created for a variety of applications.^45^ The initial precursor, which is primarily the MAX phase, is where the fundamental M_*n*+1_X_*n*_ chemistry and structure of MXenes are generated, suggesting that proper MAX phase synthesis is crucial for the successful generation of MXenes. Reactive sintering of elemental powder at high temperatures, typically 1350–1600 °C, is the standard technique for MAX phase synthesis. Fig. 3 illustrates the formation of a layered crystalline structure. Since the “A” layer is more reactive than the M_*n*+1_X_*n*_ structure and the metallic M–A bonds in the MAX phases are comparatively weaker than the covalent/ionic M–X interactions, which are sometimes challenging to break, Traditionally, the “A” atomic layers from the MAX phase precursors are selectively etched to produce MXenes. Fluoride-containing acids have been widely utilized as etchants for this. Multilayered MXene powders are produced following the selective etching process, and they can be delaminated into single flakes of M_*n*+1_X_*n*_T_*x*_ sheets^46,47^ (Fig. 3).

Nowadays, the synthesis methods of MXenes can be classified broadly into top-down and bottom-up methods. Because of their low cost, ease of usage, and scalability for large-scale 2D MXene synthesis, the top-down methods have been the primary and preferred method for MXenes synthesis since the discovery of MXenes. Although bottom-up approaches are more complex, expensive, and yield smaller-scale production, they are preferred for producing high-quality 2D MXenes since top-down methods often result in lower-quality products. The ‘A’ atomic layers from their MAX phases can be selectively etched to create MXenes using the top-down method, whereas MXenes can be built from an atomic or molecular scale using the bottom-up method.^49,50^

**Fig. 4:**
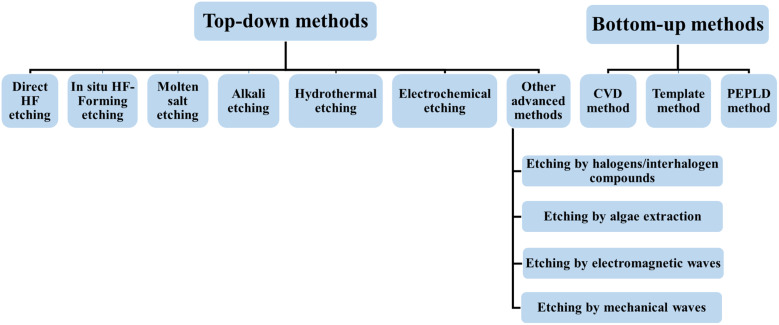
Flow chart illustrating different top-down and bottom-up synthesis methods of MXene.

“In Fig. 4”, a flow chart illustrating various top-down and bottom-up synthesis strategies for MXenes is displayed. The characterization of MXenes is pivotal for understanding their structure, properties, and potential applications. A variety of characterization techniques are frequently employed to analyze and confirm MXene's structure, morphology, and properties, including X-ray diffraction (XRD), X-ray photoelectron spectroscopy (XPS), transmission electron microscope (TEM), scanning electron microscope (SEM), energy-dispersive X-ray spectroscopy (EDS), atomic force microscope (AFM), ultraviolet-visible spectroscopy (UV-vis), *etc.* It is important to consider that the actual properties can differ depending on the specific formulations, synthesis techniques, and intended applications.

### 2.1. Top-down methods

**Fig. 5:**
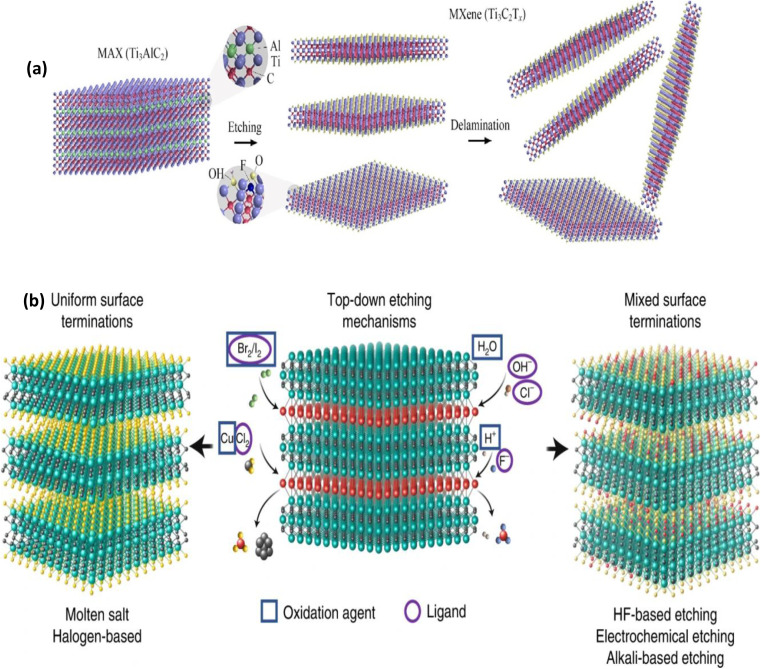
(a) Schematic of the top-down method for Ti_3_C_2_T_*x*_ synthesis. Reproduced with permission from ref. 54 Copyright 2023, Elsevier. (b) Different top-down etching routes for MXene synthesis. Etching routes are categorized based on the uniformity of T_*x*_ surface terminations in the resultant MXenes after etching. Molten salt and halogen-based etching processes yield MXenes with uniform surface terminations (left), whereas HF-, LiF/HCl-, NaOH/KOH-solution and electrochemical etching produce MXenes with mixed surface terminations (right). Reproduced with permission from ref. 50 Copyright 2023, Elsevier.

The “A” atoms (A = Al, Sn, Ga, Zn, *etc.*) from the MAX phase or (A–X) m from the non-MAX phase precursors of MXenes are selectively etched using top-down techniques, as was previously indicated. Because they allow for the synthesis of products on a greater scale and at a cheaper cost, top-down techniques are especially noteworthy in the industrial field.^49,51^ The surface of the resulting MXenes is frequently adorned with a range of concentrations of surface functional groups, regardless of the etchant. “Oxygen (O), hydroxyl (OH), and/or fluorine (F)” are the most prevalent surface groups on MXenes. It has been demonstrated that the synthesis process affects the concentration of each surface termination^52^ (Fig. 5a). The ternary titanium aluminum carbide (Ti_3_AlC_2_) is still the most studied MAX phase and was the first to be etched. Several etching techniques have been discovered to convert it into Ti_3_C_2_T_*x*_ MXene.^53^ Fluoride-containing methods including direct hydrofluoric acid (HF), *in situ* HF, and molten fluoride, as well as fluoride-free methods like electrochemical etching and alkali, are all included in the top-down approach (Fig. 5b).

#### 2.1.1. Synthesis of MXenes in fluoride-based acid etchants

There are numerous etching methods for creating MXenes. The primary and most popular method is etching in fluoride-containing aqueous solution, which is accomplished by soaking MAX phase powders in acid solutions containing fluoride, such as HF and fluoride salt with hydrochloric acid (HCl)^55^ (see Fig. 5). Although other compounds have been identified for etching, fluoride-based materials continue to be the most often used reagents in the manufacture of MXenes.^56^

##### 2.1.1.1. Direct HF etching

**Fig. 6:**
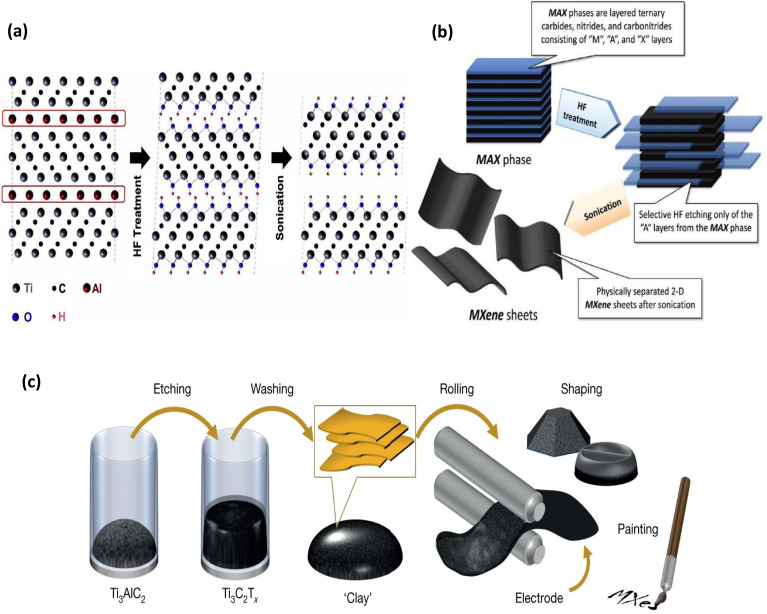
(a) Schematic of the exfoliation process for Ti_3_AlC_2_ showing the replacement of Al atoms by OH after reaction with HF. Reproduced with permission from ref. 59 Copyright CC BY 3.0 2021, IOP Science, (b) schematic showing the preparation of MXene sheets by HF chemical etching of MAX phase, followed by sonication. Reproduced with permission from ref. 60 Copyright CC BY 3.0 2020, Wiley. (c) Schematic representation of clay-like Ti_3_C_2_T_*x*_ MXene paste preparation. Reproduced with permission from ref. 61 Copyright 2021, Wiley-VCH.

Direct HF etching is a basic and often used technique for creating MXenes, and HF solution was the first known etchant to extract MXenes from their corresponding MAX precursors.^57^ It was initially documented by Naguib *et al.* in 2011,^58^ and since then, many additional publications have synthesized MXenes in the same manner. In this groundbreaking study, Ti_3_AlC_2_ (MAX phase) powder was left at room temperature (RT) for two hours while submerged in a 50% concentrated HF solution. As a result, Ti_3_C_2_T_*x*_ multilayers held together by hydrogen and/or van der Waals bonds were obtained by selective etching of the Al atomic layers from Ti_3_AlC_2_ and their replacement by surface termination groups (–F/–OH) due to the high reactivity between the Al-containing MAX phase and F ions. The resulting suspension was then washed several times using deionized water and sonicated in methanol to separate the MXene layers (Fig. 6a and b). The reactions of the HF solution with Ti_3_AlC_2_ are as follows^58^ (see Fig. 6a and b):

1 Ti_3_AlC_2_ + 3HF → AlF_3_ + 3/2H_2_ + Ti_3_C_2_

2 Ti_3_C_2_ + 2H_2_O → Ti_3_C_2_(OH)_2_ + H_2_

3 Ti_3_C_2_ + 2HF → Ti_3_C_2_F_2_ + H_2_

It is strongly advised to adopt the HF etching technique because of its simplicity and ease of use. It is particularly taken into consideration when preparing MXene at a low reaction temperature. Fully fluoride-terminated MXenes have been seen to have strong oxidation resistance, which increases their stability. Moreover, additional advantages of HF aqueous etching include enhanced yield and morphology as well as complete removal of the Al layer from the MAX phase. Because of these benefits, researchers mostly employed this technique to produce MXenes.^12,25^ However, HF is a very poisonous and corrosive chemical that can cause long-term damage by penetrating the tissues of muscles, bones, and skin. As a result, HF must be handled and disposed of very carefully. As a result, a number of substitute techniques were created to reduce or eliminate the need for concentrated HF and to make the reaction less hazardous, milder, safer, and more ecologically friendly.^53,61^Table 1 delivers the benchmarking of MXene synthesis approaches.

**Table 1:** Quantitative benchmarking of MXene synthesis routes

Synthesis method	Medium	Yield (%)	BET (m^2^ g^−1^)	Conductivity (S cm^−1^)	Interlayer spacing (Å)	Ref.
HF etching	HF (40–50 wt%)	85–93	20–65	8 × 10^3^ to 1.0 × 10^4^	12.0 ± 0.3	58
*In situ* HF (LiF/HCl)	LiF + HCl (1 : 10 M)	88–91	40–70	5 × 10^3^ to 6 × 10^3^	12.5 ± 0.2	62
Alkali-hydrothermal	NaOH or KOH (6–10 M)	90–92	80–120	3 × 10^3^ to 5 × 10^3^	13.2 ± 0.4	63
Molten-salt	ZnCl_2_/NaCl (550–600 °C)	78–82	25–45	3 × 10^3^ to 4 × 10^3^	11.8 ± 0.3	12
Electrochemical	NH_4_Cl/NaCl electrolyte	40–50	50–90	2 × 10^3^ to 2.5 × 10^3^	13.0 ± 0.5	64
Bio-assisted	Algae-plant extract	85–90	70–110	2 × 10^3^ to 3 × 10^3^	13.5 ± 0.5	65

##### 2.1.1.2. *In situ* HF-forming etching

In order to selectively etch Al from the MAX phase precursors, a gentler etching technique based on the *in situ* creation of HF upon interaction of an acid with a fluoride salt was devised, taking into account the harmful nature of HF acid. Ghidiu *et al.* first presented this acid/fluoride salt etching technique in 2014 (ref. 66) etching Al from Ti_3_AlC_2_ with a solution of lithium fluoride (LiF) salt and HCl. As demonstrated by eqn (4), HF is still created *in situ* in this instance. This method successfully produced Ti_3_C_2_T_*x*_ in the form of wet clay that could be moulded and dried to produce highly conductive shapes, hence called the “clay” method^66^ (Fig. 6c).

4 LiF + HCl → HF + LiCl

**Fig. 7:**
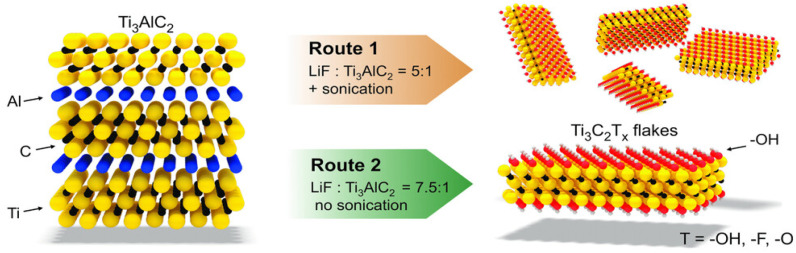
Summary of the clay method (Route 1) and MILD method (Route 2), where schematic structures of Ti_3_AlC_2_ and Ti_3_C_2_T_*x*_ and differences in the LiF to Ti_3_AlC_2_ ratio are noted. Reproduced with permission from ref. 68 Copyright CC BY 3.0 2016, Wiley-VCH.

In this method, Li^+^ cations spontaneously intercalate between MXene sheets to enhance reduce inter-flake contacts and the interlayer gap, making delamination simpler. The properties and performance of MXenes are directly related to the synthesis conditions used. For example, the molar ratio of LiF to MAX phase and the acid concentration affect whether sonication is needed for delamination or whether manual shaking suffices. The optimized “minimally intensive layer delamination” (MILD) method improves yield and flake size by adjusting these ratios^66–70^ (see Fig. 7).

Despite the effectiveness of HF-based and *in situ* HF-forming methods like LiF/HCl in producing high-quality MXenes, their reliance on hazardous chemicals continues to pose serious safety and environmental risks. This is especially important in biomedical applications in which even small residual HF levels cause dramatic cytotoxicity or cell death. An additional disadvantage of fluoride etchants is a high density of fluoride ion (F^−^) on MXene surfaces, which decreases the percentage of more functionally diverse groups like –OH and –O. Although it is difficult to conjugate F^−^ groups chemically, –OH and –O terminations are much better candidates for additional functionalization. Therefore, fluoride-free etching processes are thought to be more desirable to customize surface terminations, particularly to MXenes targeted towards biological or environmentally friendly applications.^70^

#### 2.1.2. Molten salt etching

##### 2.1.2.1. Molten flouride salt etching

**Fig. 8:**
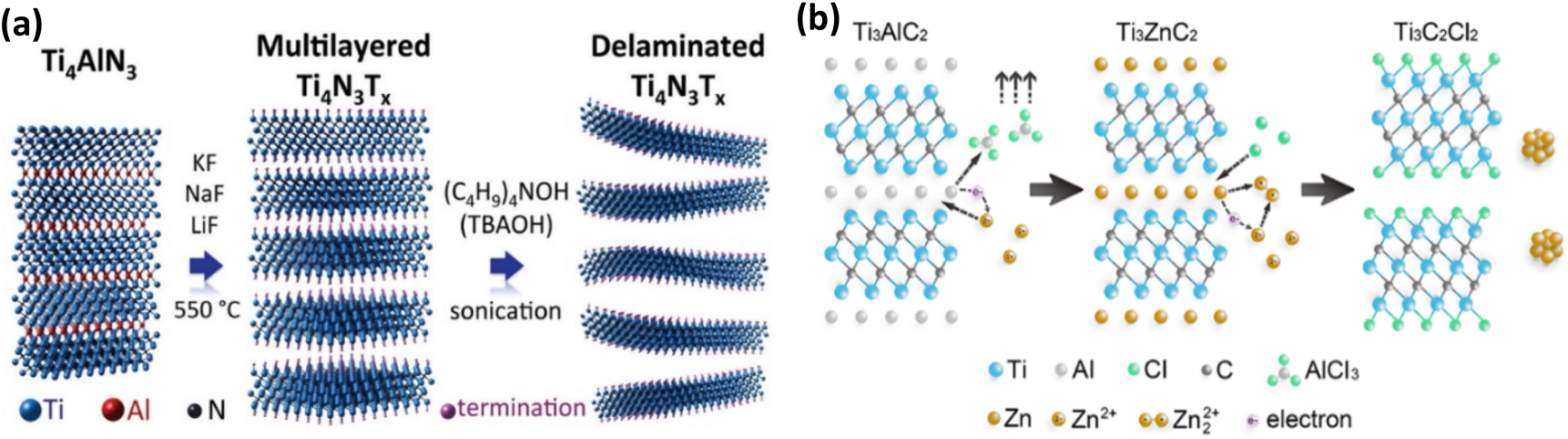
(a) Schematic illustration of the synthesis of the first nitride MXene (Ti_4_N_3_T_*x*_) by the molten salt approach. Reproduced with permission from ref. 72. Copyright 2017, Elsevier. (b) Schematic illustration of the synthesis of Zn-based MAX phase and Cl^−^ terminated MXene through Lewis acid molten salt. Reproduced with permission from ref. 73 CC BY 3.0, 2020 MDPI publisher.

For the etching of nitride-based MAX phases and the production of 2D nitride MXenes, the use of acidic solutions—like those in the preceding sections—has not worked out. This was thought to be because Ti_*n*+1_N_*n*_ has a lower cohesive energy than its comparable carbides and carbonitrides, which suggests that it is not as structurally stable and dissolves easily in aqueous solutions containing fluoride. However, theoretical calculations verified the thermodynamic limitation to etch the Ti_*n*+1_AlN_*n*_ MAX phase because of the strong bonding between Ti and Al atoms, confirming that the transformation from Ti_*n*+1_AlN_*n*_ to Ti_*n*+1_N_*n*_ had a higher energy barrier than the transformation from Ti_*n*+1_AlC_*n*_ to Ti_*n*+1_C_*n*_.^49,61^ To overcome such problems and to successfully synthesis nitride MXenes, another sophisticated technology, molten salt etching technique, was devised. In this procedure, MAX phase precursor is etched by molten salt under high temperatures in a short period to form MXenes. In 2016, Urbankowski *et al.* successfully synthesized the first nitride MXene (Ti_4_N_3_T_*x*_) using this method. They did this by heat-treating a mixture of Ti_4_AlN_3_ powder and molten fluoride salt (a mixture of 59% potassium fluoride (KF), 29% lithium fluoride (LiF), and 12% sodium fluoride (NaF)) at 550 °C for 30 minutes under argon (Ar) gas flow. This allowed the Al sheet to be etched out of the Ti_4_AlN_3_ MAX phase, which was then delaminated^71^ (see Fig. 8 a).

##### 2.1.2.2. Lewis acid molten salt etching

Lewis acid molten salt melts are a non-toxic and more ecologically friendly choice. Li *et al.* showed in 2019 how to create Cl-terminated MXene, like Ti_3_C_2_Cl_2_, by employing molten ZnCl_2_ to redoxally replace Al in the MAX phase with Zn (Fig. 8b).^74^ The procedure enables etching to proceed with Si, Ga, and Zn-containing MAX phases in addition to those containing Al.^75^

The MXene family is expanded by MXenes made in this manner, which frequently exhibit smoother surfaces and distinct terminations.^50,76,77^ This method of synthesising MXenes was first documented in 2019 by Li *et al.*^74^ This work involved a replacement reaction between the Zn element from molten ZnCl_2_ and the Al element in MAX phase precursors (Ti_3_AlC_2_, Ti_2_AlC, Ti_2_AlN, and V_2_AlC). Cl-terminated MXenes (Ti_3_C_2_Cl_2_ and Ti_2_CCl_2_) and many new MAX phases (Ti_3_ZnC_2_, Ti_2_ZnC, Ti_2_ZnN, and V_2_ZnC) were generated by the replacement reaction. The removal of the Al atomic layer from the MAX phase was made easier by the strong acidity of a Lewis acid, like ZnCl_2_, in its molten form.

By etching Ti_3_AlC_2_ with ZnCl_2_ molten salt at 550 °C in an Ar gas atmosphere, Ti_3_AlC_2_ MXene was produced, for instance. A new Zn-MAX phase (Ti_3_ZnC_2_) is created when Zn^2+^ cations react with Al layers during the etching process and then occupy the Al sites in Ti_3_AlC_2_. Additionally, as seen in Fig. 8b, the excess ZnCl_2_ erased the Zn atoms in the interlayer of Ti_3_ZnC_2_ to create Ti_3_AlC_2_ MXenes. The following is a summary of the reactions that were involved:^74^

5 Ti_3_AlC_2_ + 1.5ZnCl_2_ → Ti_3_ZnC_2_ + 0.5Zn + AlCl_3_

6 Ti_3_ZnC_2_ + ZnCl_2_ → Ti_3_C_2_Cl_2_ + 2Zn

After carefully analyzing the mechanism of the molten salt technique, Li *et al.* in 2020 developed a broad Lewis acid etching route that went beyond etching only Al-containing MAX phases to non-Al-MAX phases (*e.g.*, Si, Zn, and Ga). This method created a variety of MXenes from their MAX phases by using the redox interaction between the A element and the cation in the Lewis acid molten salt. MXenes could be made in this way by mixing Lewis acid molten salts with MAX phases and heating them to 750 °C for 24 hours.^75^

#### 2.1.3. Alkali etching

Alkaline etching is an alternate method for creating MXenes from MAX phases that uses potent bases such as potassium hydroxide (KOH) and sodium hydroxide (NaOH), are used. The alkalis utilized for synthesis are regarded as etching agents because of their strong affinity for amphoteric elements (*i.e.*, Al, Ga, Pb, and Sn), which can be etched by both acid and alkali treatments.^57,78^ In contrast to more toxic etchants such as HF, alkaline etching employs hydroxide ions (OH^−^) to etch away the “A” layer (predominantly Al), resulting in a cleaner and safe process. Alkaline solution etching involves less toxic chemistry overall and with safe handling practices in place, such as neutralization and treating alkaline byproducts, the byproducts are less toxic to minimize the effect on the environment. Although this method can be slower and less efficient, it has the advantage of being less toxic and potentially causing fewer defects in the MXene structure.^76,79^

**Fig. 9:**
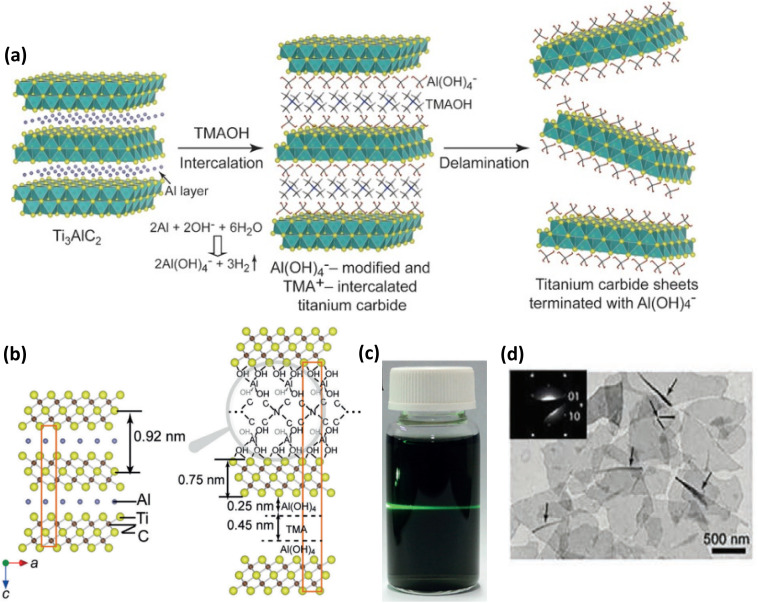
(a) Schematic illustration of the etching of Ti_3_AlC_2_ using an organic base, TMAOH, which helps in the breaking of Ti–Al bond through Al hydrolysis, and TMA^+^ acts as an intercalant which helps in the delamination of MXene flakes. Reproduced with permission from ref. 80 Copyright 2016, Wiley-VCH. (b) Structural illustration of Ti_3_AlC_2_ before and after the reaction with TMAOH. Reproduced with permission from ref. 80 Copyright 2016, Wiley-VCH. (c) Photograph of the MXene nanosheets dispersion in H_2_O showing an apparent Tyndall effect. (d) Representative bright-field TEM image of MXene nanosheets. Reproduced with permission from ref. 80 Copyright 2016, Wiley-VCH.

According to a 2016 study by Xuan *et al.*, the strong reactivity between the organic base tetramethylammonium hydroxide (TMAOH) and Al atoms allowed TMAOH to be used as an etchant and an intercalant to create titanium carbide (Ti_3_C_2_) sheets from the Ti_3_AlC_2_ MAX phase. Ti_3_C_2_ was created by immersing the Ti_3_AlC_2_ powders in 25% aqueous TMAOH after they had been pretreated for 30 minutes in a low-concentration (10–20 wt%) HF solution. In this etching process, TMAOH interacted with Al atoms after intercalating into the MAX phase's interlayers, which led to the hydrolysis of Al to yield Al(OH)_4_^−^. Because of the negative charge of Al(OH)_4_^−^, it would instantly be prone to bonding with the surface Ti metals of Ti_3_C_2_ sheets through O–Ti bonds, thus resulting in the surface functionalization by Al(OH)_4_^−^ ions followed by TMA^+^ ion intercalation, producing delaminated Ti_3_C_2_ MXene sheets terminated by Al(OH)_4_^−^ without any sonication (Fig. 9a). Fig. 9b shows the structure of Ti_3_AlC_2_ both before and after the reaction with TMAOH. The interlayer gap is noticeably larger. The as-obtained nanosheet dispersion in H_2_O (Fig. 9c) demonstrated obvious Tyndall effects, indicating significant hydrophilicity of the delaminated Ti_3_C_2_. The TEM image in Fig. 9d, which shows a 2D structure for the delaminated Ti_3_C_2_, was used to examine the delamination effect of this method. The sheet was nearly transparent, demonstrating the efficiency of TMAOH in delamination into exceedingly thin sheets.^80^ Combining alkali and hydrothermal techniques can also be used for etching, as in some cases, higher temperatures or pressures are required to remove the Al atomic layer in an alkaline environment.^52,79^

#### 2.1.4. Hydrothermal etching

##### 2.1.4.1. *In situ* fluoro-hydrothermal etching

From etching solely Al-containing MAX phases to non-Al-MAX phases (*e.g.*, Si, Zn, and Ga), Li *et al.*^75^ presented a broad Lewis acid etching technique. The hydrothermal approach allows the reaction temperature to be higher than 100 °C when the etching process is conducted in a closed environment. It involves a reaction that takes place at high pressures and temperatures in an aqueous environment.

Since this approach can further boost an etchant's exfoliating capacity, it is mostly used in an autoclave, a sealed container that offers perfect control over both temperature and pressure. This facilitates the necessary chemical processes to create MXenes. The hydrothermal process in the closed reactor is less hazardous and more ecologically friendly than the HF etching approach. As a result, they are essential to MXenes' real-world uses.^55,56^ Using ammonium fluoride (NH_4_F) as an etchant, Maleski *et al.*^52^ created a hydrothermal method in 2016 to create Ti_3_C_2_T_*x*_ MXene from their MAX phase Ti_3_AlC_2_. In this investigation, a mixture of Ti_3_AlC_2_ and NH_4_F solution was heated to 150 °C for 24 hours in a sealed Teflon-lined autoclave in order to convert Ti_3_AlC_2_ to Ti_3_C_2_T_*x*_.

Following the hydrolysis of NH_4_F to produce NH_3_·H_2_O and HF, HF reacted with Ti_3_AlC_2_ to produce Ti_3_C_2_T_*x*_ MXene. Temperature, time, and NH_4_F concentrations can all affect the structure and morphology of the resulting Ti_3_C_2_T_*x*_. The following reactions took place during the etching procedure:^81^

7 NH_4_F + H_2_O ↔ NH_3_·H_2_O + HF

8 3HF + Ti_3_AlC_2_ → Ti_3_C_2_T_*x*_ + AlF_3_ + 3/2H_2_

9 3NH_4_F + AlF_3_ → (NH_4_)_3_AlF_6_

Peng *et al.* reported using a mixture of sodium tetrafluoroborate (NaBF_4_) and HCl to synthesize Ti_3_C_2_ and Nb_2_C MXenes through a closed hydrothermal route that was carried out at 180 °C. This hydrothermal technique provided a greater degree of Al layer removal, a greater interlayer distance, and a bigger BET surface area of 2D MXenes in comparison to the conventional HF etching procedure. This was due to the hydrothermal process's gradual release of the F-mechanism, which made sonication easier to achieve delamination.^82^

In terms of avoiding the direct use of concentrated HF, this method is straightforward, one-step, and suitable for large-scale synthesis. Notwithstanding these benefits, fluoride terminations are still present in the synthesized MXenes.^45^

The following are the reactions that take place:^82^

10 NaBF_4_ + HCl → HBF_4_ + NaCl

11 HBF_4_ → HF + BF_3_

12 BF_3_ + 3H_2_O → 3HF + H_3_BO_3_

13 Ti_3_AlC_2_ + 3HF → AlF_3_ + 3/2H_2_ + Ti_3_C_2_

14 Ti_3_C_2_ + 2H_2_O → Ti_3_C_2_(OH)_2_ + H_2_

15 Ti_3_C_2_ + 2HF → Ti_3_C_2_F_2_ + H_2_

16 Nb_2_AlC + 3HF → AlF_3_ + 3/2H_2_ + Nb_2_C

17 Nb_2_C + 2H_2_O → Nb_2_C(OH)_2_ + H_2_

18 Nb_2_C + 2HF → Nb_2_CF_2_ + H_2_

##### 2.1.4.2. Fluoride-free hydrothermal etching (alkali-assisted hydrothermal etching)

There have been proposals for fluoride-free etchants that combine hydrothermal and alkali treatments.^52^ Multilayer Ti_3_C_2_T_*x*_ MXene (T = OH, O) was successfully created by Li *et al.*^83^ In 2018 utilising an alkali-assisted hydrothermal process using an aqueous NaOH solution as the etchant.

**Fig. 10:**
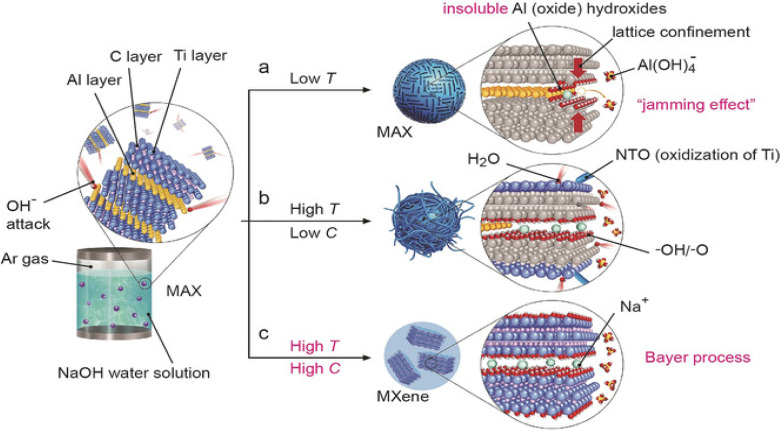
The reaction between Ti_3_AlC_2_ and NaOH/water solution at different NaOH concentrations and temperatures. (a) Al (oxide) hydroxides hinder the Al extraction process under low temperatures, and this jamming effect blocks the MXene formation. (b) Some Al (oxide) hydroxides dissolve in NaOH under high temperatures, but the low NaOH concentrations and high-water content lead to the oxidation of MXenes and yield NTOs. (c) According to the Bayer process, high temperatures and high concentrations of NaOH facilitate the dissolution of the Al (oxide) hydroxides in NaOH. Reproduced with permission from ref. 83 Copyright 2018, Wiley-VCH.

Typically, Ti_3_AlC_2_ was heated hydrothermally to a high temperature of 270 °C after being added to a 27.5 M NaOH solution. As depicted in Fig. 10. This process can only produce a Ti_3_C_2_T_*x*_ MXene at high temperatures and high NaOH concentrations. A Bayer method utilized in the refinement of bauxite served as the model for this technique. They stated that Al was successfully extracted from Ti_3_AlC_2_ using this process, and that high-quality Ti_3_C_2_T_*x*_ powder with 92% purity was produced. When the reaction temperature was lowered from 270 to 250 °C, it was found that the etching process's efficiency dropped. The MAX phase interacts with OH in this hydrothermal reaction, which oxidizes the Al atoms in the Al layer to produce –OH and –O terminated MXene, Al(OH)_4_, which dissolves in the alkali, and H_2_ gas, as shown in eqn (19) and (20). This is the first successful synthesis of a high-purity MXene using the alkali solution approach and opens the door for the potential successful synthesis of fluorine-free MXenes using this method. Compared to MXenes from the HF etching technique, MXenes from this synthesis route will have more –OH and –O terminations, which will improve their supercapacitor performance. The etching process involves the following reactions:^83^

19 Ti_3_AlC_2_ + OH^−^ + 5H_2_O → Ti_3_C_2_(OH)_2_ + Al(OH)_4_^−^ + 5/2H_2_

20 Ti_3_AlC_2_ + OH^−^ + 5H_2_O → Ti_3_C_2_O_2_ + Al(OH)_4_^−^ + 7/2H_2_

Fig. 10 illustrates that the two main variables influencing the reaction kinetics as well as the quality and purity of as-synthesized MXenes are hydrothermal temperature and NaOH concentration. The application of this high-temperature hydrothermal treatment in high-concentration alkali solutions is limited due to the substantial risks and hazards involved, even though etching the MAX phase with concentrated alkali is an efficient technique that can produce highly hydrophilic products with F-free terminations. Furthermore, the resultant MXenes often have an accordion-like morphology and are multilayer; further intercalation and delamination are required to produce single-layer MXene nanosheets.^61,77^

#### 2.1.5. Electrochemical etching

Generally, Electron transfer is a natural electrochemical phenomenon that occurs as part of the surface reaction in chemical etching techniques for MAX phases. Hence, electrochemical etching, which uses the MAX phase as an electrode in electrolytes like HCl, NaCl, NH4Cl, *etc.*, to selectively etch the “A” atomic layer at a certain voltage, may be a viable substitute technique for MXene synthesis.^61,78,84^

Both cathodic and anodic reactions take place on the interfaces between the electrodes and the etching solution in electrochemical etching, as opposed to the chemical etching covered in the preceding sections. To create an electric field, electrodes of different voltages are inserted into the electrolyte, or etching solution. By controlling the voltage differential (etching potential) between the “A” and “M” atomic layers within the reaction potential range, the “A” atomic layer in the MAX phase can be selectively etched.^55^

This method yielded MXenes with –Cl, –O, and/or –OH surface terminations. Both the “A” and “M” layers may be eliminated using this electrochemical etching technique, which over-etches and produces carbide-derived carbons (CDCs). Therefore, to selectively erode the “A” atomic layers without over-etching and preserve the 2D structure of MXenes, a careful balancing of etching settings is necessary.^56^

**Fig. 11:**
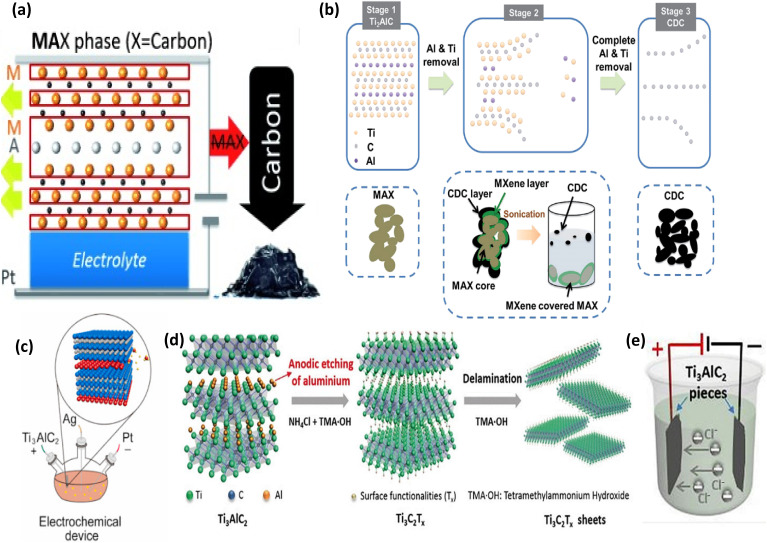
The working principle of electrochemical etching. (a) Schematic representation of the room-temperature synthesis of CDC from the MAX phase. Reproduced with permission from ref. 85 Copyright CC BY 3.0, 2014, Wiley-VCH. (b) The proposed mechanism for the electrochemical etching of Ti_2_AlC in an aqueous HCl electrolyte. Reproduced with permission from ref. 86 CC BY 3.0, Copyright 2024, Wiley-VCH. (c) Scheme of the three-electrode electrochemical etching system. Reproduced with permission from ref. 87 Copyright CC BY 3.0, 2025, Elsevier. (d) Schematic of the anodic etching and delamination process of bulk Ti_3_AlC_2_ in a binary aqueous electrolyte. (e) The configuration of an assembled electrochemical cell using bulk Ti_3_AlC_2_ as the anode and cathode in a binary aqueous electrolyte. (d and e) Reproduced with permission from ref. 88 Copyright CC BY 3.0, 2018, Wiley-VCH.

Using diluted NaCl, HCl, and HF as the electrolytes, CDCs were produced from the MAX phases (Ti_3_AlC_2_, Ti_2_AlC, and Ti_3_SiC_2_) by the electrochemical technique. Both the Ti and Al atoms were eliminated during this room-temperature electrochemical anodic etching of the MAX phases at a constant current density of 100 mA cm^2^, which produced a mostly amorphous CDC with a restricted pore size distribution^85^ (see Fig. 11a).

In 2017, Sun *et al.* successfully reported the production of F-free Ti_2_CT_*x*_ MXene for the first time using the electrochemical etching of the Al layers from porous Ti_2_AlC electrodes in diluted HCl aqueous electrolyte. This study used a three-electrode setup, with low-concentration HCl (1 or 2 M) aqueous solution acting as the electrolyte, porous Ti_2_AlC electrodes acting as the working electrode (anode), and Pt foil acting as the counter electrode (cathode) (see Fig. 11c). A reference electrode was Ag/AgCl in 3 M KCl. The HCl electrolyte helped to produce Ti_2_CT_*x*_ MXene and etch Al. However, continuous etching leads to the conversion of the outer layer of MXene to CDC and the inner MAX core to MXene. Therefore, a unique three-layered core–shell structure is obtained, consisting of an outer CDC layer on the surface, an intermediate MXene layer, and an inner unetched MAX phase core. By using bath sonication, MXenes can be further isolated from this three-layer structure (Fig. 11b).^89^

This electrochemical etching process produces MXenes with just –Cl, –O, and –OH surface terminations and does not use any fluoride ions during the etching process. However, because CDC layers grow and cover the unreacted MAX phases during the etching process, which slows the etching process, this technique is relatively sluggish and only yields a modest amount. Therefore, in order to achieve large-scale synthesis of MXenes, more effective techniques must be devised to remove the CDC layers, as this strategy is not suitable for mass manufacturing.^55,56^

In 2018, Yang *et al.*^88^ demonstrated another F-free etching method based on the anodic etching of Ti_3_AlC_2_ in an alkaline mixture of 1 M ammonium chloride (NH_4_Cl) and 0.2 M TMAOH as the electrolyte solution for the fabrication of Ti_3_C_2_T_*x*_ MXene (T = O, OH) with a yield of >40%. More than 90% of the exfoliated MXene were mono or bilayers after TMAOH delamination, making this approach a promising etching procedure (see Fig. 11d). In order to prevent etching from occurring solely on the surface and to prevent the CDC layer from interfering with the etching process, a binary aqueous electrolyte was employed to enable the electrolyte ions to enter the layers deeply. Two pieces of the bulk MAX phase Ti_3_AlC_2_ were used as the anode and cathode in a two-electrode arrangement, as shown in Fig. 11e. The cathode served as a counter electrode, and only the anode participated in the etching reactions. With this technique, the electrolyte's chlorine ions (Cl^−^) broke the Ti–Al bonds, allowing the Al atoms to be removed selectively. By opening the margins of the etched anode, the subsequent intercalation of ammonium hydroxide (NH_4_OH) into the layers allowed for more etching. With ambient conditions and a small voltage of +5 V, this approach produced larger MXene sheets and higher yields in just 5 hours. The mechanism of the etching process is as follows:^88^

21 Ti_3_AlC_2_ − 3e^−^ + 3Cl^−^ → Ti_3_C_2_ + AlCl_3_

22 Ti_3_C_2_ + 2OH^−^ − 2e^−^ → Ti_3_C_2_(OH)_2_

23 Ti_3_C_2_ + 2H_2_O → Ti_3_C_2_(OH)_2_ + H_2_

Because electrochemical etching frequently uses mild reaction conditions and aqueous electrolytes, reducing the need for harsh chemicals and minimizing environmental impact, it is becoming a more viable, safe, and economical method for producing MXenes. It also offers a non-toxic alternative to conventional fluoride-based etching. In addition, this procedure uses less energy than high-temperature techniques because it may be done at room temperature. In addition to the inadequate yield, the presence of the CDC layer remains a problem to be solved.^55,76,90^ A summary of top-down methods for MXenes synthesis is introduced in Table 2.

**Table 2:** A summary of top-down methods for MXenes synthesis

Methods	Precursors	MXenes	Etchants	Etching conditions	Surface terminations	Ref.
HF etching	Ti_3_AlC_2_	Ti_3_C_2_T_*x*_	50 wt% HF	RT, 2 h	–F, –OH	58
*In situ* HF-forming etching	Ti_3_AlC_2_	Ti_3_C_2_T_*x*_	5 M LiF + 6 M HCl	40 °C, 45 h	–F, –OH, –O	66
	Ti_3_AlC_2_	Ti_3_C_2_T_*x*_	12 M LiF + 9 M HCl	RT, 24 h	–F, –OH, –O, –Cl	67
			2 M NH_4_HF_2_	RT, 24 h	–F, –OH, –O	67
Molten fluoride salt etching	Ti_4_AlN_3_	Ti_4_N_3_T_*x*_	KF + LiF + NaF	550 °C, 0.5 h	–F, –OH, –O	71
Lewis acid molten salt etching	Ti_3_AlC_2_	Ti_3_C_2_Cl_2_	ZnCl_2_	550 °C, 5 h	–Cl	74
	Ti_2_AlC	Ti_2_CCl_2_	ZnCl_2_	550 °C, 5 h	–Cl	74
	Ti_3_SiC_2_	Ti_3_C_2_T_*x*_	CuCl_2_	750 °C, 24 h	–O, –Cl	75
	Ti_2_AlC	Ti_2_CT_*x*_	CuCl_2_/CdCl_2_	650 °C, 24 h		
	Ti_3_AlC_2_	Ti_3_C_2_T_*x*_	CuCl_2_/FeCl_2_/CoCl_2_/NiCl_2_	700 °C, 24 h		
	Ti_2_GaC	Ti_2_CT_*x*_	CuCl_2_	600 °C, 24 h		
	Nb_2_AlC	Nb_2_CT_*x*_	AgCl	700 °C, 24 h		
	Ti_3_AlCN	Ti_3_CNT_*x*_	CuCl_2_	700 °C, 24 h		
	Ta_2_AlC	Ta_2_CT_*x*_	AgCl	700 °C, 24 h		
	Ti_3_ZnC_2_	Ti_3_C_2_T_*x*_	CdCl_2_/FeCl_2_/CoCl_2_/NiCl_2_/AgCl	650 °C −700 °C, 24 h		
Alkali etching	Ti_3_AlC_2_	Ti_3_C_2_T_*x*_	10–20% HF+ 25% aqueous TMAOH	RT, 0.5 h + RT, 24 h	–Al(OH)_4_^−^	80
Alkali-assisted hydrothermal etching	Ti_3_AlC_2_	Ti_3_C_2_T_*x*_	27.5 M NaOH	270 °C, 12 h	–OH, –O	83
Hydrothermal etching	Ti_3_AlC_2_	Ti_3_C_2_T_*x*_	NH_4_F	150 °C, 24 h	–F, –OH, –O	81
	Ti_3_AlC_2_	Ti_3_C_2_T_*x*_	NaBF_4_ + HCl	180 °C, 8–32 h	–F, –OH	82
	Nb_2_AlC	Nb_2_CT_*x*_		180 °C, 15–35 h		82
Electrochemical etching	Ti_2_AlC	Ti_2_CT_*x*_	2 M HCl	+0.6 V, 120 h	–OH, –O, –Cl	89
	Ti_3_AlC_2_	Ti_3_C_2_T_*x*_	1 M NH_4_Cl + 0.2 M TMAOH	+0.5 V, RT, 5 h	–OH, –O	88
Other etching methods	Ti_3_AlC_2_	Ti_3_C_2_T_*x*_	Br_2_	RT, 8 h	–Br, –I	91
			I_2_	70 °C, 8 h		
			ICl	−78 °C, 4 h		
			IBr	RT, 8 h		
	Ti_3_AlC_2_	Ti_3_C_2_T_*x*_	I_2_	100 °C, 4 days	–OH, –O	92
	V_2_AlC	V_2_C	Algae	RT, 24 h	–OH, –O	65
	Mo_2_Ga_2_C	Mo_2_C	Ultraviolet light (100 W), H_3_PO_4_	3–5 h	–O	93
	Ti_3_AlC_2_	Ti_3_C_2_T_*x*_	Surface acoustic waves (SAWs), LiF (∼0.05 M)	Milliseconds	–F, –OH, –O	94

#### 2.1.6. HF-free routes (comparative summary)

To consolidate the fluoride-independent top-down approaches discussed in the subsections titled “Fluoride-free molten salt etching,” “Alkali etching,” “Fluoride-independent hydrothermal etching (alkali-assisted),” “Electrochemical etching,” and “Other advanced top-down methods,” Table 3 compares key metrics across representative routes: reaction temperature and time, yield/purity, flake characteristics after delamination (lateral size, thickness), and dominant surface terminations (T_*x*_).

**Table 3:** HF-free top-down routes to MXenes: comparative summary of conditions, yields, flake size and surface terminations

Route (HF-free)	Representative system (s)	Etchant/medium	Typical conditions (*T*, time)	Yield/purity	Flake characteristics (after delamination)	Dominant surface terminations (T_*x*_)	Notes/trade-offs
Lewis-acid molten salt	Ti_3_C_2_Cl_2_, Ti_2_CCl_2_; also non-Al MAX	Molten ZnCl_2_ (Lewis acid)	550 °C (Ar); generalized route up to 750 °C, 24 h	NR	Often smoother surfaces; distinct terminations	Cl (Cl-MXenes)	Broader scope (Al, Si/Ga/Zn MAX); device-friendly Cl termination; high-*T* solid-state processing
Alkali-assisted hydrothermal	Ti_3_C_2_T_*x*_	NaOH (27.5 M) in sealed autoclave	270 °C, high [NaOH]; produces MXene only at high *T*/concentration	≈92% purity	Multilayer “accordion”; intercalation/delamination needed	–O/–OH-rich	Fluoride-free; safety improved *vs.* HF, but high-*T*, concentrated alkali; good for energy (supercapacitors) due to O/OH terminations
Electrochemical (HCl)	Ti_2_CT_*x*_ from Ti_2_AlC	Dilute HCl (1–2 M), three-electrode	∼RT; time NR; forms CDC outer layer if over-etched	Low/modest (sluggish due to CDC)	Core–shell (MAX/MXene/CDC) forms; sonication helps isolate	–Cl/–O/–OH (no F)	Fluoride-free but CDC formation slows etch; optimize to avoid over-etching
Electrochemical (binary electrolyte)	Ti_3_C_2_T_*x*_ from Ti_3_AlC_2_	NH_4_Cl (1 M) + TMAOH (0.2 M); two-electrode	+5 V, ∼5 h, RT	>40% yield; >90% mono/bi-layers after delamination	Large flakes possible; high monolayer fraction	–O/–OH (±–Cl); no F	Mild, scalable, equipment-light; manage CDC risk with binary electrolyte diffusion
Iodine-assisted (halogen)	Ti_3_C_2_T_*x*_	I_2_ in CH_3_CN, HCl for delamination	25–100 °C, 1–6 h (typical)	NR	Avg. lateral size ∼1.8 µm; 71% of sheets <5 nm thick; stable films in water ∼2 weeks	–O/–OH-rich	Fluoride-free; good flake size and film conductivity; multi-step workflow
Halogen/interhalogen (ambient)	Ti_3_C_2_T_*x*_ (T = Br, I)	I_2_, Br_2_, IBr, ICl	RT (ambient)	NR	NR	–Br/–I (as reported)	Very mild; terminations controlled by halogen; data on yield/size often case-specific
Bio-etching (“green”)	V_2_C from V_2_AlC	Algae extract (biochemicals)	RT, ∼24 h	∼90% yield	Lateral 50–100 nm; thickness ∼1.8 nm	NR	Green, low-cost; purity control can be challenging; promising for biocompatible use
UV-induced selective etching	Mo_2_C from Mo_2_Ga_2_C	H_3_PO_4_ + UV	∼25–60 °C, 3–5 h (stirring under UV)	NR	2D mesoporous Mo_2_C (chemistry-dependent)	Likely –O/–OH (phosphate work-up); no F	Fast, device-oriented porosity; dependent on UV-responsive precursors

In short, Lewis-acid molten salts (*e.g.*, ZnCl_2_) provide Cl-terminated MXenes with smooth surfaces in high-temperature solid-state processes—good for interconnects/EMI shielding/shielding but necessitating high *T* and special handling. Alkali-assisted hydrothermal and electrochemical processes function without fluorides and provide, respectively, commonly preferred O/OH-rich terminations for electrochemistry and separation, and ambient-temperature processing with yields that are cell design- and voltage-dependent. The iodine/halogen-assisted methods can be carried out in ambient to moderate temperatures and can facilitate control over terminations and sheet size, although frequently involving multi-step workups. The bio-etching (“green”) processes provide mild, low-temperature processing that is attractive for safety and possible biocompatibility yet remains early in scalability. The UV/photochemical methods (*e.g.*, non-fluoride acid photolyses) facilitate high-speed, selective etching and can add mesoporosity, favoring mass-transport-limited applications. See Table 3 for summary of detailed conditions and associated trade-offs (HF-free MXene processes and key figures).

#### 2.1.7. Other advanced top-down methods

In addition to the most widely utilised techniques mentioned above, researchers have consistently put forth intriguing new ways to create MXenes. Using halogen (I_2_, Br_2_) and interhalogen compounds (IBr, ICl) to etch Al atoms off Ti_3_AlC_2_ at ambient temperature, Jawaid *et al.* successfully synthesised Ti_3_C_2_T_*x*_ (T = Br, I) MXene in 2021.^91^

**Fig. 12:**
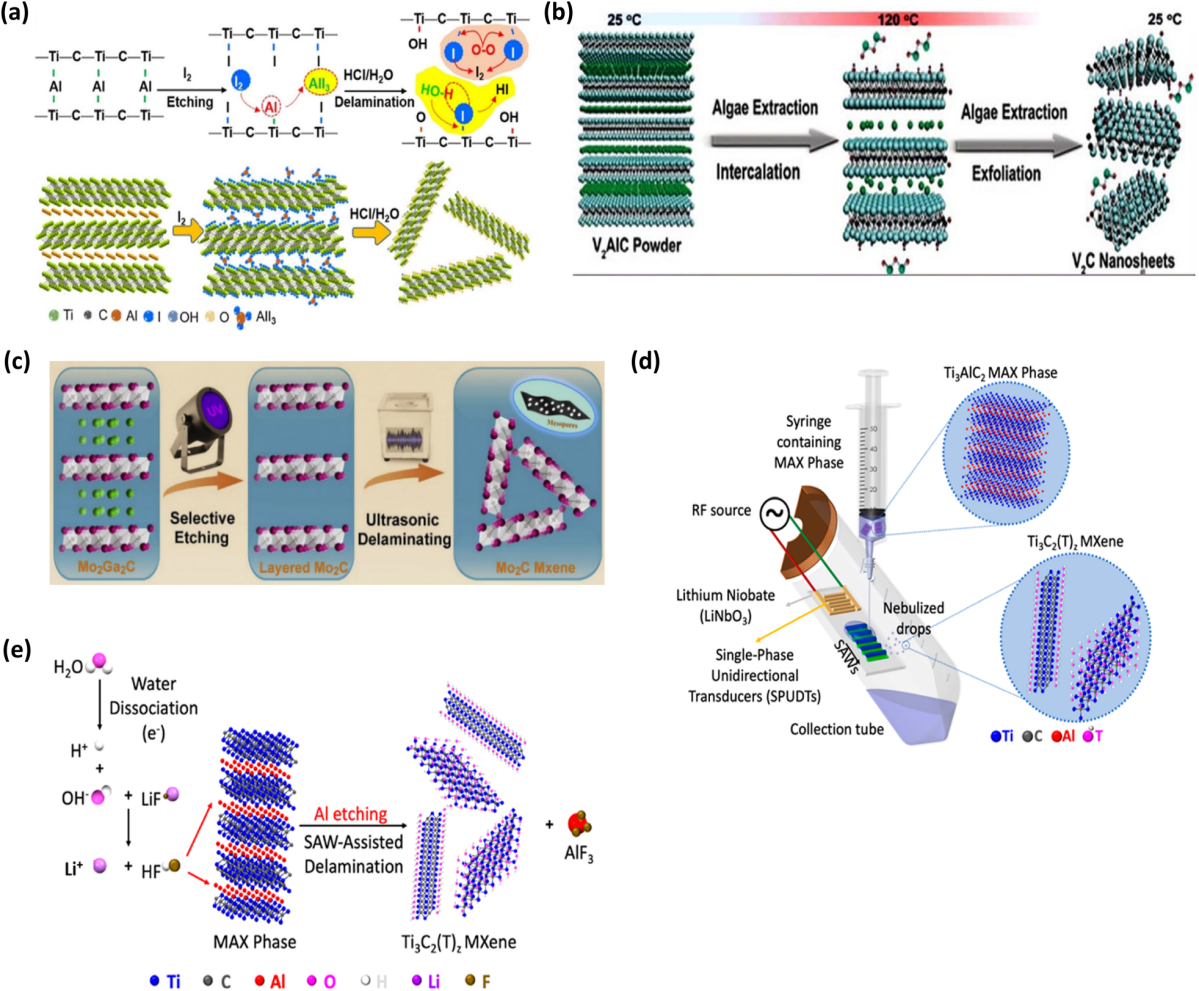
Other methods for the preparation of MXenes. (a) Iodine-assisted etching and delamination of Ti_3_AlC_2_ to form 2D MXene sheets. Reproduced with permission from ref. 92 Copyright CC BY 3.0, 2021, Wiley-VCH. (b) Schematic illustration of the production of V_2_C nanosheets *via* algae extraction. Reproduced with permission from ref. 65 Copyright CC BY 3.0, 2020, Wiley-VCH. (c) Schematic illustration of 2D mesoporous Mo_2_C MXene synthesis *via* a UV-induced selective etching method. Reproduced with permission from ref. 95 Copyright 2020, Elsevier. (d and e) Schematic representation of the experimental setup and underlying physiochemical mechanism responsible for the SAW facilitated derivation of Ti_3_C_2_T_*x*_ MXene. Reproduced with permission from ref. 96, Copyright 2022, Elsevier.

Additionally, by etching with iodine (I_2_) in anhydrous acetonitrile (CH_3_CN) and then delaminating in HCl solution, Shi *et al.* showed how to manufacture Ti_3_C_2_T_*x*_ (T = O, OH) MXene sheets from the MAX phase using an iodine-assisted etching method (Fig. 12a). When the temperature was raised from room temperature (25 °C) to 100 °C, the Al concentration in the iodine-etched samples obtained following the delamination in HCl dropped from 16.7 weight percent in the MAX phase to 0.9 weight percent. It was found that the performance increased proportionately with the temperature increase. As a result of this etching technique, oxygen-rich Ti_3_C_2_T_*x*_ sheets with an average size of 1.8 µm were created. Over 71% of sheets have a thickness of less than 5 nm. Additionally, it has been noted that MXene sheets have outstanding thin-film conductivity and are very stable in water for up to two weeks.^92^

To create mass vanadium carbide (V_2_C) MXene from the V_2_AlC MAX phase for use as a photothermal agent (PTA) in photothermal therapy (PTT) applications, Zada *et al.* presented a unique green synthesis technique in 2020 (ref. 65) (Fig. 12b). Proteins, lipids, carbs, oil, polyunsaturated fatty acids, minerals, and bioactive substances are all present in algae extraction. It was hypothesized that the organic acids in the algae extraction could efficiently attack the V–Al bonds and result in the intercalation of a large number of bioactive compounds between the V_2_C layers during the etching process. This resulted in V_2_C MXene nanosheets with good structural integrity by further speeding up the delamination and cleavage process in a more cooperative way.

After a full day of etching at room temperature, a considerable number of V_2_C nanosheets with a high yield (90%) and lateral sizes ranging from 50 to 100 nm and an average thickness of roughly 1.8 nm were produced using this etching process.

By avoiding the use of conventional concentrated alkali or acid solutions as an etchant, the algae extraction etching method has the unique advantage of being comparatively biocompatible when compared to traditional chemical treatments. Furthermore, algae are relatively cost-effective and can be cultivated using sunlight. Therefore, an environment-friendly, simple, low-cost, and high-yielding delamination method has been developed to break down MAX phase and create MXenes with desirable properties for a wide range of applications. These multiple advantages make this method superior to other approaches used for MXene development. However, maintaining purity during the etching process and ensuring its reliability is challenging.^57,65^

The “A” layers can likewise be etched away from the layered ternary precursors using mechanical and electromagnetic waves. To create fluoride-free mesoporous molybdenum carbide (Mo_2_C) MXene from its precursor (Mo_2_Ga_2_C), Mei *et al.* suggested a new and efficient UV-induced selective etching technique (Fig. 12c). This technique to etch the layers of gallium (Ga) atoms away from the Mo_2_Ga_2_C precursor was suggested because of the excellent UV-responsive property of Mo_2_Ga_2_C. This process involved adding the Mo_2_Ga_2_C precursor to a solution of phosphoric acid (H_3_PO_4_). After that, this combination was agitated for three to five hours while exposed to UV light. To get layered MXenes that exhibited a typical 2D graphene-like morphology and a high degree of purity with an overall thickness of about 6 nm. Using UV-sensitive precursors, it has been demonstrated that high-yield production of 2D MXene structures with high-quality may be created in a matter of hours without the use of dangerous and extremely caustic acids.^93^

El-Ghazaly *et al.* reported an ultrafast (approximately millisecond) Ti_3_AlC_2_ MAX phase to Ti_3_C_2_T_*x*_ MXene conversion by exposing surface acoustic waves (SAWs) to an aqueous mixture of MAX phase and a low concentration of LiF (∼0.05 M), this exposure leads to proton production, and these protons decrease the pH of the solution, which in turn increases the dissociation of LiF. The protons produced from SAWs with fluorine ions from LiF combine to produce *in situ* HF that selectively etches away the Al from Ti_3_AlC_2_ MAX phase to produce Ti_3_C_2_T_*x*_ MXene (see Fig. 12d and e).^94^

Beyond their safer chemistry, these “green” routes also show promising performance. For example, algae-extract bio-etching at room temperature (∼24 h) produced V_2_C MXene at ∼90% yield, with lateral flake sizes 50–100 nm and average thickness ∼1.8 nm, and films stable in water for ∼2 weeks.^65^ UV-induced selective etching of Mo_2_Ga_2_C in H_3_PO_4_ under UV (3–5 h, 25–60 °C) yields 2D mesoporous Mo_2_C within hours without concentrated hazardous acids; while overall yield was not reported, the rapid processing and built-in mesoporosity are attractive for mass-transport-limited applications. These quantitative characteristics complement the environmental profile of green etchants and provide benchmarks to compare with the HF-free routes summarized in Table 3.

### 2.2. Bottom-up methods

Despite the scalability advantages of using bulk precursors, a bottom-up method is better for creating a material from its constituent composition. This is explained by bottom-up synthesis's capacity to precisely control the material's chemistry, making it possible to design custom materials. Bottom-up synthesis usually begins with small organic or inorganic molecules or atoms. After that, crystal formation occurs, which can be structured to form layered structures in two dimensions. Bottom-up synthesis methods provide you more control over the growth settings and are used to synthesize high-purity MXenes.^10^

2D MXenes with a variety of potential applications in electronics, optoelectronics, and photovoltaics can be synthesized using bottom-up methods. These materials usually have good crystalline quality and fewer defects and impurities. Recently, several bottom-up methods have been developed to create MXenes, such as chemical vapor deposition (CVD), the template approach, plasma-enhanced pulsed laser deposition (PEPLD), *etc.*^49,51,61,70^

#### 2.2.1. Chemical vapor deposition (CVD)

The most popular bottom-up method for creating relatively pure materials with a controlled structure at the atomic or nanoscale level over the last 30 years has been chemical vapour deposition (CVD), which involves the chemical reactions of gaseous precursors in an active (plasma, heat, and light) environment.^56^

**Fig. 13:**
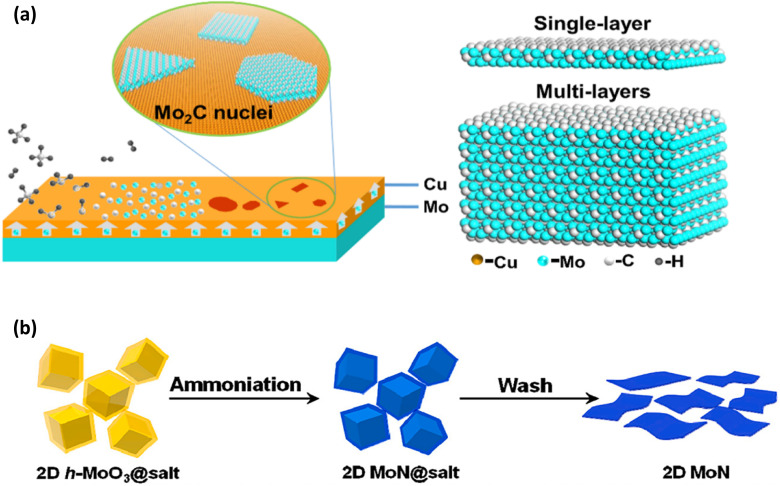
(a) Schematic diagram showing the growth process of 2D α-Mo_2_C crystals using a CVD method and the atomic models of 2D α-Mo_2_C crystals with different thicknesses. Reproduced with permission from ref. 53 Copyright 2019, Elsevier. (b) Schematic illustration of synthesis of 2D ultrathin MoN nanosheets using the salt-assisted template method. Reproduced with permission from ref. 53 Copyright 2019, Elsevier.

In order to create solid, thin films of MXenes, this process typically involves introducing precursor gases containing the components required for the synthesis of MXenes into a vacuum-heated reaction chamber. The gases then react at high temperatures on a heated substrate. With its ability to precisely regulate growth factors like pressure, temperature, gas composition, *etc.*, CVD is a very successful process for synthesising MXenes and producing high-quality thin films. Typically, CVD creates ultrathin, multi-layered MXene films^70,97^ (at least six layers). First reported by Xu *et al.*^98^ in 2015, CVD produces superb ultrathin transition metal carbide (TMC) crystals. The CVD development process is schematically shown in (Fig. 13a). As the growth substrate, they initially positioned an ultrathin copper (Cu) foil on top of a molybdenum (Mo) foil. Then, in the presence of H_2_ gas, this stack of Cu/Mo foils was heated to a temperature higher than Cu's melting point (1085 °C), allowing Cu to melt and Mo atoms to diffuse to the liquid Cu surface. Then, by breaking down on the Cu surface, a tiny quantity of methane (CH_4_) was added as the carbon source. This reacted with the Mo atoms and created 2D Mo_2_C crystals on the liquid Cu surface. The Mo_2_C crystals' thickness, nucleation density, and lateral dimension can all be easily customized by varying the experimental parameters, such as growth time and operating temperature.

This bottom-up CVD approach can be used as a strategy for a controlled growth of high-purity MXenes with fewer defects and impurities compared to the top-down approaches. However, it has a low yield, which makes it less suitable for large-scale production and commercial manufacturing. Moreover, MXenes synthesized by CVD exhibit a lack of surface terminations, which may not be optimal for biomedical applications. Thus, the surface chemistry of MXenes with no or limited kinds of functional groups should be considered.^43,49,99^

#### 2.2.2. Template method

Template-based techniques have also been developed for the synthesis of 2D transition metal carbides (TMCs) and nitrides (TMNs) in addition to CVD. To create 2D TMCs or TMNs, all template methods start with 2D transition metal oxide (TMO) nanosheets as templets, which are subsequently carbonised or nitrided. Depending on the structure of the corresponding TMOs utilized, the synthesized TMCs and TMNs have different structures. By reducing hexagonal oxides in ammonia (NH_3_), Xiao *et al.*^100^ initially reported salt-templated synthesis in 2017 for the synthesis of 2D molybdenum nitride (MoN), vanadium nitride (V_2_N), and tungsten nitride (W_2_N). As an illustration, the production of 2D MoN nanosheets entailed the following four fundamental processes (see Fig. 13b).^48,51,100^

(I) 2D-template preparation: using the Mo precursor for MoO_3_ synthesis.

(II) Template coating with salt: 2D hexagonal MoO_3_-coated NaCl (2D h-MoO_3_@NaCl) was synthesized by annealing the MoO_3_ with NaCl salt under Ar atmosphere at 280 °C.

(III) Ammoniation: the produced 2D h-MoO_3_@NaCl powders were slowly ammoniated at 650 °C in NH_3_ atmosphere to yield MoN@NaCl.

(IV) Template removal: using deionized water to remove the salts.

In order to demonstrate that different 2D metal oxides produced using the salt-template method might serve as building blocks for the production of other 2D metal carbides and nitrides, Xiao *et al.* have used this procedure to prepare W_2_N and V_2_N.^100^ Although the template method is more time-consuming and less scalable than more industrial methods like HF etching, it has significantly higher yields and is more economical than CVD, making it a better option for synthesizing MXene in some applications where high-scale production and lower costs are crucial.^79^

#### 2.2.3. Plasma-enhanced pulsed laser deposition (PEPLD)

**Fig. 14:**
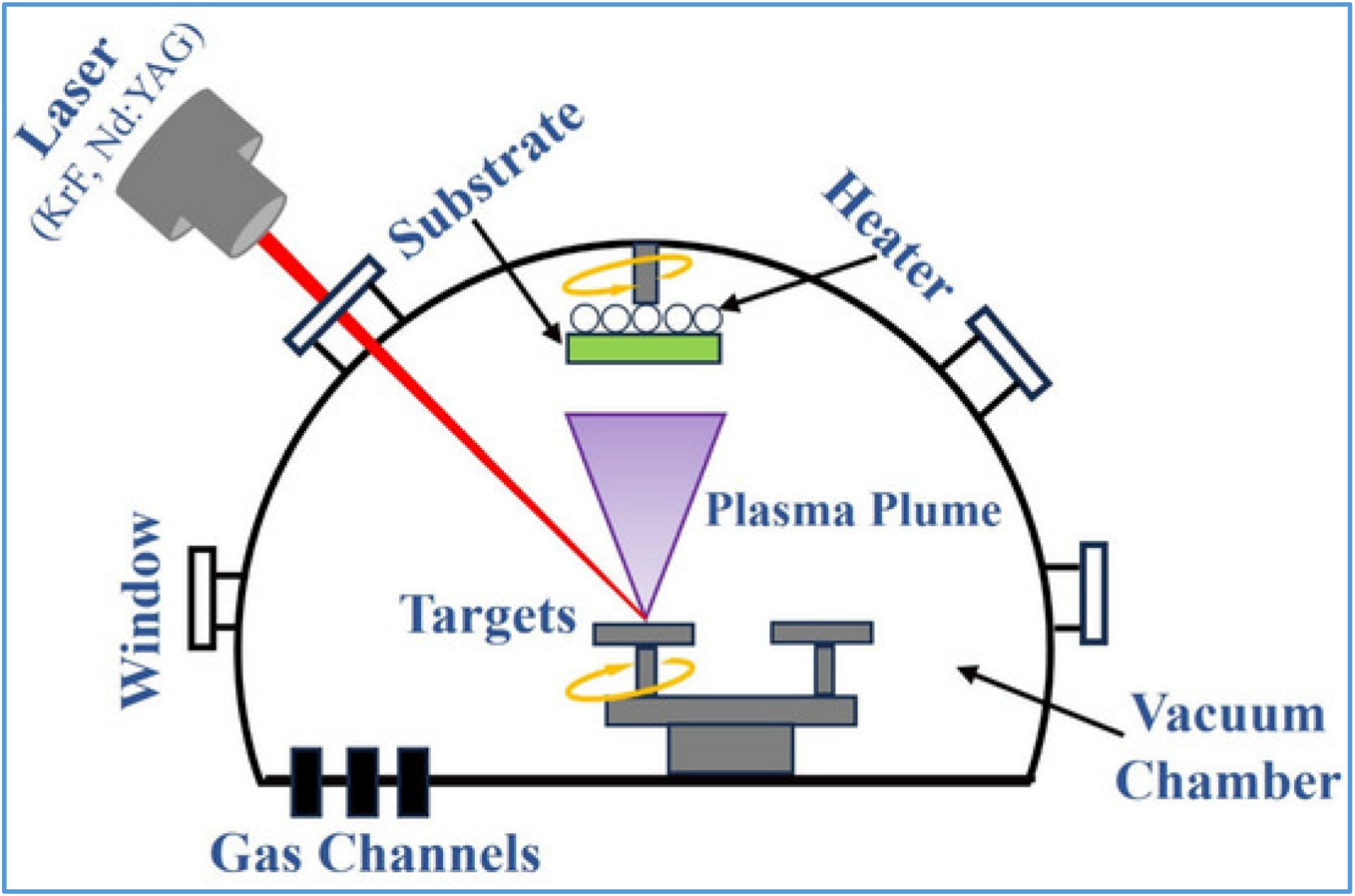
The working principle for the preparation of 2D materials using the PLD technique. Reproduced from ref. 102, Copyright CC BY 3.0, 2025 MDPI publisher.

Plasma enhancement was originally adopted in the conventional high-temperature CVD method to enable reduced temperature and higher quality synthesis, which is called plasma enhanced chemical vapor deposition (PECVD). PEPLD allows single-crystal films to grow continuously by combining the benefits of PECVD and pulsed laser deposition.^51^ In 2017, Zhang *et al.*^101^ proposed the first PELPD-synthesized ultrathin Mo_2_C films using CH_4_ plasma as a carbon source, which reacted with the produced Mo vapor by the pulsed laser. The PEPLD system was equipped with a high-voltage electrode to generate CH_4_ plasma at the chamber's CH_4_ entrance using this method. The general set-up fpr PEPLD is shown in (Fig. 14). In order to furnish the Mo source, a KrF laser beam with a wavelength of 248 nm was focused simultaneously on a Mo metal target. To create high-quality films, this reaction was carried out on a sapphire substrate that had been heated to 700 °C. It was found that CH_4_ plasma favors the formation of Mo_2_C as there was no Mo_2_C deposited without ionized CH_4_, which shows that ionized CH_4_ plasma plays a key role in the synthesis process of Mo_2_C films. The resulting 2D face-centered cubic (FCC) shaped Mo_2_C films are smooth and uniform, and the laser pulse rate may be adjusted to manage the film thickness. Zhang and colleagues' efforts. Suggested a method for producing high-quality, large-area TMCs at comparatively low temperatures.^101^

Nevertheless, compared to films produced by CVD, these resulting films have substantially inferior crystalline quality.^53,79^

#### 2.2.4. Benchmarking and reporting MXene quality

To enable fair comparison across HF-free and conventional routes, we recommend reporting a minimal set of quality metrics alongside synthesis details (Table 4). Lateral flake size distributions should be extracted from SEM/TEM (AFM for small flakes) and given as a median with interquartile range (IQR) or D10/D50/D90 from at least *N* ≥ 300 flakes, noting the segmentation method/software. Thickness/layer count should be determined by AFM step heights on single flakes and/or XRD (002) spacings for ensembles, reporting mode/median plus IQR and indicating the intercalant and wet *vs.* dried state. Structural defect density from HRTEM/STEM should include areal defects (per 100 nm^2^) and edge/step density (µm^−1^), with electron dose and magnification specified. XPS should deconvolute M 2p, X 1s, O 1s, F 1s, Cl 2p, and C 1s to obtain termination fractions (–O/–OH/–F/–Cl), a metal-vacancy proxy (*e.g.*, M:(X + C) deficiency relative to stoichiometry), and—where applicable—Ti^3+^/Ti_total for Ti_3_C_2_T_*x*_; report charge neutralization, fitting model, and sampling depth. Thermal stability is captured by the oxidation onset temperature (*T*_onset) from TGA in air using a consistent criterion (*e.g.*, tangent-intercept at 5% mass loss), together with heating rate, sample mass, and gas flow. Film transport should include sheet resistance (Ω □^−1^) and conductivity (S cm^−1^) for a film of known thickness and density (four-point probe), including anneal/humidity and geometry. Colloidal stability should be reported as *ζ*-potential at a fixed pH and ionic strength (*e.g.*, pH 7.0 ± 0.2 in 1 mM NaCl) with dispersion concentration and aging time. Phase/stacking should include XRD (002) position and FWHM (plus SAED where relevant). Purity should state residual A-element and salts/intercalants *via* XPS/ICP-OES and TGA-MS, with detection limits.

**Table 4:** Standard MXene quality metrics and how to report them

Metric	Why it matters	Primary technique(s)	What to report (units)	Notes/standardization
Lateral flake size distribution	Affects percolation, membrane pores, electrode packing	SEM/TEM (image analysis); AFM for small flakes	Median ± IQR (µm); or D10/D50/D90; *N* ≥ 300 flakes; segmentation method	State dispersion/deposition; exclude aggregates; report scale calibration
Thickness/layer count	Governs conductivity, ion transport	AFM step height; XRD (002) spacing	Mode/median thickness (nm) ± IQR; (002) *d*-spacing (Å)	Specify substrate, tip, humidity; note intercalant/wet *vs.* dry
Defect density (structural)	Impacts mobility, catalytic sites	HRTEM/STEM; SAED	Defects per 100 nm^2^; edge/step density (µm^−1^)	Report dose, magnification; avoid beam-damage artifacts
Termination chemistry & vacancies	Controls hydrophilicity, work function, stability	XPS (M 2p, X 1s, O 1s, F 1s, Cl 2p, C 1s)	Fraction of –O/–OH/–F/–Cl (%); Ti^3+^/Ti_total or M-vacancy (%); C/O ratio	Provide fitting constraints, reference energies, neutralizer settings, sampling depth
Oxidation onset temperature	Stability/handling window	TGA (air); DSC-TGA (optional)	*T*_onset (°C) with definition; heating rate (°C min^−1^); gas flow (sccm)	Use same onset criterion across samples; report sample mass and pan type
Electrical transport (films)	Device relevance	Four-point probe; profilometry	Sheet resistance (Ω □^−1^), conductivity (S cm^−1^), thickness (nm), density (g cm^−3^)	State anneal/temp/humidity; substrate; measurement geometry
Colloidal stability/*ζ*-potential	Processability, ink stability	ELS/*ζ*-meter	*ζ* at pH (value) and ionic strength (mM); concentration (mg mL^−1^)	Use standard electrolyte (*e.g.*, 1 mM NaCl) and report aging time
Phase/stacking order	Ion pathways, swelling	XRD; SAED	(002) position & FWHM; turbostratic features	Report scan rate, step size; fit model for FWHM
Residual A-element/salts	Purity, performance	XPS; ICP-OES; TGA-MS	A-element at%; residual salt/intercalant (wt%)	Include detection limits and calibration method

In summary, while MXenes show noteworthy properties for energy storage, sensing, catalysis, and biomedicine, their commercial translation is constrained by synthesis-related issues, counting safety, scalability, control of surface terminations, and oxidation stability. Addressing these limitations through greener etching chemistries, scalable production methods, and surface engineering strategies will be crucial for bridging the gap between laboratory research and industrial implementation.

## 3. Physicochemical properties of MXenes

The amazing and adjustable physicochemical features of MXenes, which are two-dimensional sheets generated from transition metal MAX phases, have drawn a lot of interest. MXenes, which are composed of transition metal carbide, nitride, or carbonitrides, have a special combination of mechanical toughness, rich surface chemistry, and excellent conductivity. Their atomic arrangement—typically an interleaved transition metal layer (M), an interleaved layer of carbon or nitrogen (X), and surface functional groups like –OH, –F, or –O—is the source of these characteristics. A wide range of characteristics, including electronic conductivity, magnetism, hydrophilicity, optical activity, and chemical reactivity, are influenced by the structure, composition, and surface terminations of MXenes. Because of their inherent adaptability, MXenes can be functionalized and chemically formulated to suit specific needs. As a result, MXenes have shown considerable promise in a variety of sectors, including enhanced electrical applications, environmental cleanup, sensing, energy storage, and catalysis. The primary physicochemical characteristics of MXenes are examined in the paragraphs that follow, arranged according to their structural, electrical, optical, thermal, magnetic, and mechanical characteristics. Understanding these characteristics is essential for developing MXenes for intended technological uses as well as for investigating new uses for basic science and technology advancement.

### 3.1. Structure of MXenes

**Fig. 15:**
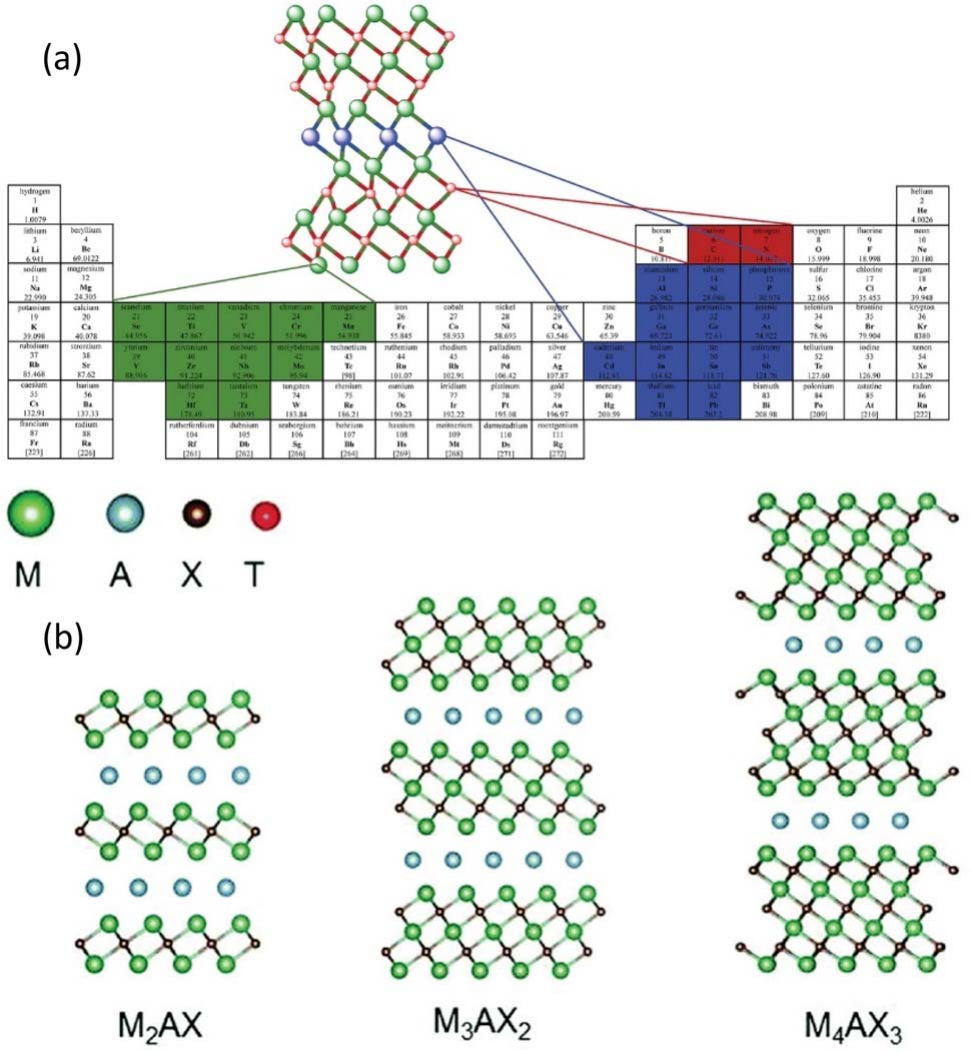
(a) Elemental selection map for MAX phases (M_*n*+1_AX_*n*_), showing the most common elements used for transition metal M (green), A-group element (light blue), and X (carbon or nitrogen) in red/blue overlays on the periodic table. Reproduced from ref. 112 Copyright CC BY 3.0, 2021, Wiley, (b) Schematic representation of MAX phase structures (M_2_AX, M_3_AX_2_, M_4_AX_3_) and their transformation into MXenes *via* selective etching: hydrofluoric acid (HF) for multilayer MXenes or a combination of lithium fluoride (LiF) and hydrochloric acid (HCl) for monolayer MXenes. The green, light blue, brown, and red spheres represent M, A, X, and surface terminations (T_*x*_ = O, OH, F), respectively (adapted with permission from refs. Reproduced from ref. 112 Copyright CC BY 3.0, 2021, Wiley).

MXenes are an important class of two-dimensional transition metal carbides, nitrides, and carbonitrides that have a wide range of potential applications in biology, energy storage, and electronics.^103^ Etching phase complexes involving metal nitrides and metal carbides result in the creation of MXenes, a two-dimensional nanomaterial (see Fig. 15, right). According to (Fig. 15, left), “MAX” stands for “metal transition metal,” “A” for elements belonging to groups 13 and 14, and “X” for carbide, nitride, or carbonitride. The nomenclature for MXene is M_*n*+1_X_*n*_T_*x*_, where T is a functional group that caps the molecule's surface (for example, OH, F, O, and Cl, in addition to Sc or –NH_2_ from molten salt operations). For instance, M might represent early transition metals such as Ti, V, Mo, and others, while X could represent carbide, nitride, or carbonitride if *n* is between 1 and 4.^104^ Hexagonal symmetry characterises the resultant MXenes, which is in line with the previous MAX phases. Different layers of X and M alternate amongst each other in the layered atomic structure.^105^ The outside layers of o-MXenes are created only by the M′ element, whereas the inner layer or levels are formed by the M″ element. The usual formulas 
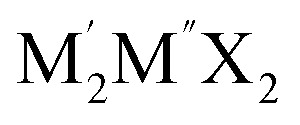
 or 
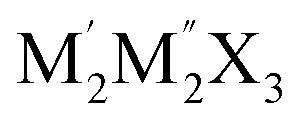
 can be used to express these compounds, which also have hexagonal symmetry. When a layer of two different transition metal elements is present, the larger M″ atoms shift somewhat from their typical positions, causing the system's symmetry to change from hexagonal to monoclinic.^105^ Exfoliation of the in-plane ordered MAX phases may result in two-dimensional i-MXenes with the formula 
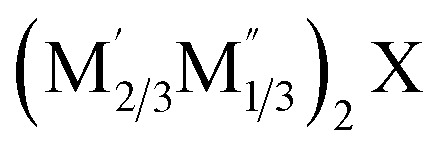
 or a two-dimensional M_1.33_X system with ordered divacancies. When the terminal functional group in MXenes causes them to display hydrophilic characteristics. Clay, colloidal, and film are just a few of the forms in which MXenes can be produced and utilised due to their special hydrophilic feature. To create nanocomposites, they can also be combined with polymer matrices. Compared to carbon nanoparticles, MXenes offer a significant advantage. Excellent conductivity is exhibited by the free-standing films produced from delaminated MXenes. Additionally, MXene compounds' single-flake form has outstanding conductivity and flexibility. The electrical and chemical characteristics of MXenes can be modified by changing the compounds chemistry. The chemical composition mostly consists of sp^2^ carbon, which is an extra advantage compared to nanoparticles based on carbon. A significant feature of MXenes is their capacity for large-scale synthesis, enabling their application in bulk forms such as extensive conductive films^106,107^ electronic interference shields, electrodes for batteries and supercapacitors, energy storage device binders and current collectors, antennas.^108,109^ Consequently, much research on MXenes has focused on large-scale assemblages of 2D flakes in film or 3D scaffold or composite form.^62^ It has been demonstrated through physical measurements of these assemblies that the intrinsic properties of MXenes interact with various interfacial processes involving 2D flakes.^110^ Measuring individual monolayer flakes of MXenes could reveal their intrinsic features; however, such measurements are currently sparse and have only been conducted on Ti_3_C_2_T_*x*_.^68,111^

### 3.2. Hydrophilicity

Among the many intriguing properties of MXenes are their excellent hydrophilicity, strong sensitivity to chemical species, and high electrical conductivity. Since their discovery, MXenes have found application in numerous technological applications due to their exceptional characteristics. These include semiconductors, supercapacitors, lithium-ion batteries, hydrogen storage, and many more.^113^ A summary of these key properties and use-cases is presented in (Table 5). Previous studies covered a wide range of MXene applications, but there has been little focus on its use in waste remediation.^114^ For instance, adsorbents based on MXene, such as Ti_3_C_2_–SO_3_H, MXene@Fe_3_O_4_, and V_2_CT_*x*_, have been investigated for the purpose of removing methylene blue (MB) from wastewater. These materials have been chosen for their anionic functional groups, excellent dispersibility, easy separation, and simple unit cell structure.^115^ Besides these benefits, MXenes have a lot of active surface sites and are hydrophilic, thus they could be able to absorb a lot of molecular or ionic species. This could be another viable way to address environmental pollutants.^113^ In order to manufacture MXenes, the most common synthetic process is the wet etching method that uses hydro-fluoric acid (HF) *in situ*. By altering the surface of the 2D sheets with oxygen-containing groups (=O and –OH), this technique makes them hydrophilic.^116^ As a result, MXenes are very compatible with water-based processes, which shows how versatile they may be for modifying system wettability and forming hybrids with other materials.^117^ Several factors impact the interaction between the solid and liquid phases, which in turn dictates how a liquid droplet would wet the surface of MXene. These factors include electrostatic reactions, hydrogen bonds, and van der Waals forces.^118^ Owing to their intrinsic two-dimensional characteristics, MXene sheets are prone to deformation, overlap, and the incorporation of various functional groups during wet-chemical synthesis and subsequent post-treatments.^119^ Consequently, it is difficult to study the interactions at the liquid–MXene interface due to the surface heterogeneities that are common on an as-synthesized sample, such as the uneven distribution of various termination types and micro/nano-structures.^120^ Conversely, the wetting characteristics of MXenes can be modulated through intentional design of surface heterogeneities, enabling their utilization in a wide array of uses, including as sensors and actuators.^121^ The wettability of a filtering membrane is crucial for the successful purification of oil/water emulsions, as good separation of water-in-oil and oil-in-water emulsions necessitates a hydrophobic or hydrophilic surface with optimal underwater wetting characteristics.^122^ Therefore, there is a remarkable opportunity to meet the needs in real-world applications through proper management of the wettability of surfaces based on MXene. MXene is a type of advanced metamaterial with significant potential, having demonstrated success in reinforced water-soluble and thermoplastic polymers.^123,124^

**Table 5:** The detailed summary of many facets concerning MXenes, emphasizing their hydrophilicity and associated properties

Feature	Details
Hydrophilicity	High affinity for water, excellent hydrophilicity
Sensitivity	Strong sensitivity to chemical species
Electrical conductivity	High electrical conductivity
Technological applications	Semiconductors, supercapacitors, lithium-ion batteries, hydrogen storage
MXene-based adsorbents	Ti_3_C_2_–SO_3_H, MXene@Fe_3_O_4_, and V_2_CT_*x*_
Target pollutant	Methylene blue (MB) in wastewater
Advantages of MXene adsorbents	Anionic functional groups, excellent dispersibility, easy separation, simple unit cell structure
Synthesis method	Wet etching method using hydro-fluoric acid (HF) *in situ*
Surface modification	Oxygen-containing groups (=O and –OH) enhance hydrophilicity
Water-based processes	High compatibility due to hydrophilicity
Factors affecting interaction	Electrostatic reactions, hydrogen bonds, van der Waals forces
Characteristics of MXene sheets	Prone to deformation, overlap, and incorporation of functional groups
Surface heterogeneities	Common on as-synthesized samples, uneven termination distribution, micro/nano-structures
Wetting characteristics modulation	Enables sensor and actuator applications *via* surface heterogeneities
Wettability of filtering membrane	Crucial for oil/water emulsion purification, needs specific underwater wetting
Real-world application potential	Proper wettability management meets real-world needs
Additional applications	Reinforced water-soluble and thermoplastic polymers

### 3.3. Conductivity

Concerns about climate change have prompted a thorough review of the energy production process, with a new emphasis on finding cheaper and more efficient ways to generate and use energy. This is particularly applicable to the thermal processes involved in electricity generation utilizing oil, nuclear fuels, and coal. Energy production systems' cooling processes are a part of this, as they are crucial to the process of energy creation and utilization and can affect the performance of these operations as a whole.^125–127^

It was shown that these coolants could have their overall thermal efficiency significantly increased when base fluids were combined with nanoparticles. According to studies, nanofluids can potentially increase the heat transfer coefficient by a large margin. In addition, these coolants might have better viscosity and effective heat conductivity and other thermophysical properties.^127^ Researchers have also determined that certain nanoparticles, when added to different base fluids at concentrations between 0.5 and 4%, can boost the effective thermal conductivity. These boosts might reach 15 to 40 percent compared to the base fluids alone.^128^

An important part of cooling is the effective thermal conductivities of nanofluids; these values are used to measure the cooling capacity and overall heat transfer rate of both new and old nanofluids; therefore, this improvement is noteworthy.^129^

An innovative two-dimensional nanoparticle called MXene has a layered structure similar to graphene. Because of its unique electrical, optical, thermal, and mechanical properties, the MXene version Ti_3_C_2_T_*x*_ has been the subject of extensive research.^130^

Researchers have utilized MXene, a prominent nanofluid filler, to enhance the effective mechanical and thermal properties of several base fluids. Solar energy collector operating fluids are a significant area. A. S. Abdelrazi, *et al.*^131^ investigated the application of a water-and-MXene nanofluid in two-stage etching and sonication-based direct absorption solar collectors (DASCs). The findings indicated that the diminished transmittance of the MXene nanofluid was attributable to its enhanced absorption of solar light.^132^ Formulated a nanofluid comprising MXene and soybean oil, using it as the working fluid in a photovoltaic/thermal (PV/T) solar collector that integrates photovoltaic and thermal energy. Nanofluids have an effective thermal conductivity that is 60.82 percent more than pure soybean oil, according to the results. One measure of thermal performance showed an improvement of approximately 24.49%. The operational fluid in solar collectors that directly absorb solar energy; it is a nanofluid composed of MXene, graphene, and water. The results showed that MXene nanosheets' Localized Surface Plasmon Resonance (LSPR) effect enhanced the optical characteristics of MXene nanofluids.^133^ Nanofluids containing ethylene glycol and MXene were studied. The nanofluids were made using either multi-layer or delaminated single-layer MXene. In contrast to the base fluid, nanofluids containing multi-layer Ti_3_C_2_T_*x*_ had an effective thermal conductivity that was 53.1% higher, while nanofluids containing single-layer Ti_3_C_2_T_*x*_ had an effective thermal conductivity that was 64.9% higher. Presented a new method for controlling nanofluidic systems by means of active ion transport over a lamellar MXene membrane that is triggered by light and driven by heat. The results indicated that this approach increased the power density of osmotic energy conversion fourfold.^134^

### 3.4. Chemical versatility

MXenes possess incredible chemical tunability, not only based on the tunability of the surface terminations, but also because they can host various catalytic functions. A milestone step towards the development of MXenes was the synthesis of Ti_3_C_2_T_*x*_ derived from Ti_3_AlC_2_, the initial synthetic MXene, which paved the way to investigate more than 70 known different compositions of MXenes based on the M, A, and X constituents.^113,135–138^

Multifunctional surface chemistry is the focus of applications relating to catalysts. Ti–OH and Ti–O functional groups are active sites for heterogeneous catalysis, facilitating reactions such as ethylbenzene dehydrogenation^139^ and electrosynthesis of ammonia (NH_3_) through N_2_ activation enhancement.^140^ Their high affinity towards the adsorption of single-atom catalysts like precious metals, *e.g.*, Pt, using surface vacancies,^141^ is another facet of their chemistry. MXenes' metal–support interactions are not what one would expect with conventional oxide supports. Under reducing conditions, metal–C bonds can lead to the formation of ordered intermetallics, modifying catalyst activity and stability.^142^ Surface engineering through partial oxidation can also produce oxide–carbide heterojunctions, further extending their catalytic versatility.^134^ Thermal processes like NH_3_ annealing can replace lattice carbon with nitrogen, which enhances the oxidation resistance at high temperatures.^134^ These processes enhance their use as supports for metal nanoparticles in catalyst systems, utilizing the nature of layered structure, high surface area, and dense functional group coverage.^135^

### 3.5. Magnetic properties

A wide variety of magnetic characteristics are displayed by the two-dimensional MXene layers. Of all the MXenes, the ferromagnets are expected to be 2D Cr_2_C, Cr_2_N, Ta_3_C_2_, and Cr_3_C_2_ phases have the potential to be exfoliated and become ferromagnets, but Ti_3_N_2_ and 2D Ti_3_C_2_ do not exhibit ferromagnetism.^138–140^ Ti_2_C and Ti_2_N were found to exhibit nearly half-metallic ferromagnetism.^141^ The Mn_2_C monolayer exhibits antiferromagnetism with a considerable Néel temperature of 720 K, according to theoretical calculations. A ferromagnetic state with a high Curie temperature of 520 K is made possible by appropriate surface functionalization with (F, OH, and Cl).^142,143^

Although ferromagnetism is expected to be present in many bare MXenes, experimentally verified MXenes are usually terminated with OH, F, O, or other atoms. As a result, creating pure MXenes like Cr_2_C is quite difficult. According to recent research, MXene's electrical conductivity can be increased by partially removing surface terminations.^144,145^

It^142,143^ is demonstrated that functionalized Cr_2_C and Cr_2_N MXenes, namely those containing F, O, H, Cl, and OH, are magnetic; nonetheless, some of them exhibit FM-AFM transitions.^146,147^

The iron atoms' d-orbitals are what give these MXenes their magnetic characteristics. Because of the significant energy difference between their ferromagnetic (FM) and antiferromagnetic (AFM) forms, chromium-based MXenes may maintain their magnetic characteristics at ambient temperature.^148^

The researchers found that ferromagnetism in non-terminated Cr_2_C was linked to the half-metallic gap of 2.85 eV. While surface terminations (F, OH, H, or Cl) may cause d-electron localization, which could result in ferromagnetic–antiferromagnetic (FM–AFM) transitions and related metal-to-insulator transitions, the mobile d-orbital electrons were also connected to ferromagnetism.^149^

Strong candidates for spintronic applications are ferrimagnetic half-metals with complete (100%) electron spin polarisation at the Fermi level. For example, oxygen-passivated Cr_2_NO_2_ has a ferromagnetic ground state with a significant half-metal gap of 2.79 eV, whereas Cr_2_N MXenes functionalized with F and OH are thought to have antiferromagnetic characteristics.^150^

Numerous mixed MXenes of Cr_2_M″C_2_T_2_ have had their magnetic properties examined. In these materials, T may be F, OH, or O, while M″ may be Ti or V +*z*/*z*. Materials can be categorized as semiconducting or metallic based on their termination, which can cause them to display nonmagnetic, antiferromagnetic, or ferromagnetic properties.^151^ The asymmetric surface functionalization allows for precise control of the magnetism of Janus MXenes M_2_XO_*x*_F_2−*x*_, where M is an early transition metal, X is carbon or nitrogen, and *x* ranges from 0.5 to 1.5, by means of tiny electric fields.^152^ Recent studies on mono-layers of Ti_2_MnC_2_T_*x*_, terminated with O, OH, and F, indicate that these materials surpass existing 2D ferromagnetic substances regarding robust ferromagnetism and elevated Curie temperatures (495–1133 K). Ferromagnetism is demonstrated by both Hf_2_MnC_2_O_2_ and Hf_2_VC_2_O_2_, as stated in the same publication. Recent studies have demonstrated that Hf_2_VC_2_F_2_ exhibits ferroelectric polarization due to its type-II multiferroic structure, which has a naturally strong magnetoelectric coupling and an in-plane non-collinear 120° Y-type antiferromagnetic order.^153,154^

### 3.6. Optical

The capacity to absorb both visible and ultraviolet light is crucial for devices using transparent conductive electrodes, photovoltaics, optoelectronics, or photocatalysis. Ti_3_C_2_T_*x*_ films that were 5 nm thick were able to absorb light in the 300–500 nm UV-vis spectrum and showed a transmittance of up to 91.2%.^155^ An important feature for photothermal treatment (PTT) applications, the film's broad and strong absorption band often appears at around 700–800 nm, contributing to its light greenish hue. The film's thickness also plays a role in this characteristic.^156^ Notably, by adjusting its thickness and ion intercalation, the transmittance values could be improved. For example, tetra methylammonium hydroxide (NMe_4_OH) enhanced the Ti_3_C_2_T_*x*_ film transmittance from 74.9 to 92.0%, but hydrazine, urea, and DMSO decreased it.^155,157^ Optic response is intimately tied to electrical and structural properties of materials. It is theoretically possible to separate the real part of a material's dielectric constant *ω*(*ε*)Re and its imaginary part *ω*(*ε*)Im when the frequency (*ε*) is varied. As with the structure and electrical properties, the optical properties of typical MXene Ti_3_C_2_T_*x*_ are greatly affected by the termination (T_*x*_).^28^ A possible influence on the imaginary part of the dielectric constant could come from electrical transitions between and within bands. The combined effect of materials' interband transitions has a direct correlation with the computed absorption spectra.^158^ A single electron can be accepted by hydroxylated or fluorinated terminations in general due to structural variations. This means that in the visible spectrum, both ends behave similarly. Alternatively, two electrons are required to stabilize oxygen termination.^149^ It has approved that functional group presence also affects the optical characteristics of these 2D compounds.^159^

The functionalization dependency optical characteristics of Ti_3_C_2_T_*x*_ using computational approaches has been discussed by Berdiyorov.^159^ They found that the functionalization made the static dielectric function less than twice as strong. Oxidized samples absorb more light in the visible region of the spectrum than pure Ti_3_C_2_ samples, while surface fluorination reduces absorption.^28^ On the other hand, oxygen terminations are characterized by their similarities to fluorinated and hydroxyl terminations. While –F and –OH terminations decrease visible-light absorption and reflectivity, they all improve UV reflectivity relative to pure MXene.^159^ The optical performance of hydroxylated/fluorinated terminations is different from that of oxygen terminations because, near the Fermi level, the total density of states of MXenes is greatly affected by oxygen atoms. When contrasted with pure Ti_3_C_2_, surface terminations increase UV reflectance and absorption. Oxygen termination enhances absorption and reflectance within the visual spectrum. In contrast, compared to pure Ti_3_C_2_, the hydroxylated and fluorinated ends are noticeably more see-through. Partial oxidation of the Ti_3_C_2_T_*x*_ surface in a humid environment is a viable option for many long-term uses.^160^

The metallic conductivity and optical transparency of MXenes make them potential transparent electrode materials for flexible electronics, and their high reflectivity in the UV range implies they could be useful as UV-blocking coatings.^159^ End results demonstrated an exceptional light-to-heat conversion efficiency of almost 100%, which has implications for biomedical and evaporation-related applications.^161^

There are several optical properties that need additional explanation before MXenes applications may advance. These include emission colors, plasmonic and non-linear optical capabilities, luminescence efficiency and more.^132,162^

### 3.7. Mechanical

The mechanical properties of MXenes are greatly affected by surface terminations. The stiffness of MXenes finished with O is expected to be much higher than that of MXenes terminated with F or OH, which show lower elastic stiffness.^38^ The reason behind this could be because MXenes with different terminations have varied lattice constants. Typically, MXenes with an O termination have smaller lattice parameters compared to those with a F or OH termination.^163^ MXenes with functionalized surfaces are more malleable than their unmodified counterparts. An example of a material whose Young's modulus is reduced by functionalization is Ti_2_C. However, functionalized Ti_2_C is more strain-tolerant than both naked Ti_2_C and graphene.^164^ When subjected to tensile deformation, the surface-terminated groups operate as a barrier between Ti_2_C and the surrounding material, slowing the collapse of Ti layers and increasing the critical strain value. In addition to the number of atomic layers, or (*n*) in the chemical formula M_*n*+1_X_*n*_, the mechanical properties of MXene are affected by this component. Bare MXenes' elastic constant and Young's modulus were studied using classical molecular dynamics. The results show that among all the Ti_*n*+1_C_*n*_ (*n* = 1, 2, 3) materials, the thinnest Ti_2_C exhibits the highest Young's modulus and an elastic constant approximately double that of MoS_2_.^165^ Results for functionalized MXene demonstrate that M_*n*+1_X_*n*_T_*x*_'s strength and hardness progressively improve with decreasing (*n*).^166^ The results show that MXenes can have their mechanical properties enhanced by mixing them with *Nanoscale Horizons*' polymers or carbon nanotubes. Combining MXenes with other polymers can enhance their toughness, durability, tensile and compressive strengths, flexibility, and toughness to varied degrees. A good example is the Ti_3_C_2_T_*x*_–polyvinyl alcohol (PVA) composite, which is very flexible and has high tensile and compressive strengths. Composites containing PVA have a tensile strength that is more than four times greater than that of pure Ti_3_C_2_T_*x*_. Also, Ti_3_C_2_T_*x*_–UHMWPE8 and Ti_3_C_2_T_*x*_–PAM are robust and have a strong yield strength.^167,168^

Amongst the most studied MXenes, Ti_3_C_2_T_*x*_ monolayers exhibit a Young's modulus in the range of about 480–500 GPa, with an elastic strain limit of roughly 3.2%. These values, obtained from both *in situ* tensile experiments and theoretical calculations, indicate excellent stiffness, though thicker multilayer flakes often show reduced performance due to defects. In comparison, Ti_2_C demonstrates an even higher theoretical Young's modulus of approximately 600 GPa. Depending on loading direction and the type of surface terminations, Ti_2_C can sustain critical strains up to nearly 9.5% under biaxial loading and as high as 18% under uniaxial tension, underscoring its potential for mechanically robust applications. Data on Mo_2_TiC_2_T_*x*_ remain limited, with most research concentrating on its synthesis and electronic properties rather than detailed mechanical characterization. Overall, the mechanical response of MXenes is strongly influenced by thickness, defect density, and surface terminations, which together govern their stiffness and strain-to-failure behavior.^168^

### 3.8. Electronic

MXenes' variable thickness, extensive surface functionalization options, and significant compositional diversity allow for the observation of a variety of electrical characteristics, including metallicity, semi conductivity, and topological insulativity.^169,170^ Numerous theoretical studies of electronic properties, such as density of states (DOS) and band structures, have been carried out. Thus far, first-principles calculations and experimental data have demonstrated that only a small fraction of MXenes are semiconductors, with the majority of functionalized MXenes predicted to be metallic or semi metallic.^141^ MXenes are classified as either topologically trivial or nontrivial, and they can be semiconductors or metal/semimetals, depending on the strength of the relativistic spin–orbit coupling (SOC).^171–173^ Examples of functionalized MXenes that change from metallic to semiconducting behavior following surface functionalization include Ti_2_CO_2_, Zr_2_CO_2_, Hf_2_CO_2_, and Sc_2_CT_2_ (T = F, OH, and O). The Generalized Gradient Approximation (GGA) for Ti_2_CO_2_ produces 0.24 eV, 0.88 eV, and 1.0 eV, while the energy gaps for Sc_2_CT_2_ with T = F, OH, and O are 1.03, 0.45, and 1.8 eV, respectively.^171,174^ Sc_2_C(OH)_2_ has a straight band gap, according to band structure simulations, whereas most semiconductors have indirect band gaps. The corresponding MXene systems exhibit comparable metallic to semiconducting behavior after the same kind of functionalization because Ti, Zr, and Hf belong to the same group of the periodic table and have the same outermost partially filled atomic orbital configurations ([Ar]3d^2^4s^2^, [Kr]4d^2^5s^2^, and [Xe]4f^14^5d^2^6s^2^).^144^ It is anticipated that the F and OH groups will have an equivalent impact on the electrical structure of an unaltered MXene system. This is because each F or OH group can only take in a maximum of one electron from surfaces. The O group differs from the F and OH groups in that two electrons must be taken off its surfaces to stabilize it. The presence of heavy 4d and 5d transition metals in various MXenes has a major impact on the electrical structures of relativistic spin–orbit coupling SOC. If the SOC is ignored, the semiconducting, semi metallic compounds Ti_3_N_2_F_2_, M_2_CO_2_ (M = Mo, W), and M_2_M′C_2_O_2_ (M = Mo, W; M′ = Ti, Zr, W) have a zero-energy gap. This is because these compounds' valence and conduction bands are parabolic around the Γ point, and they only touch at that point.^171^

## 4. Applications of Mxene

**Fig. 16:**
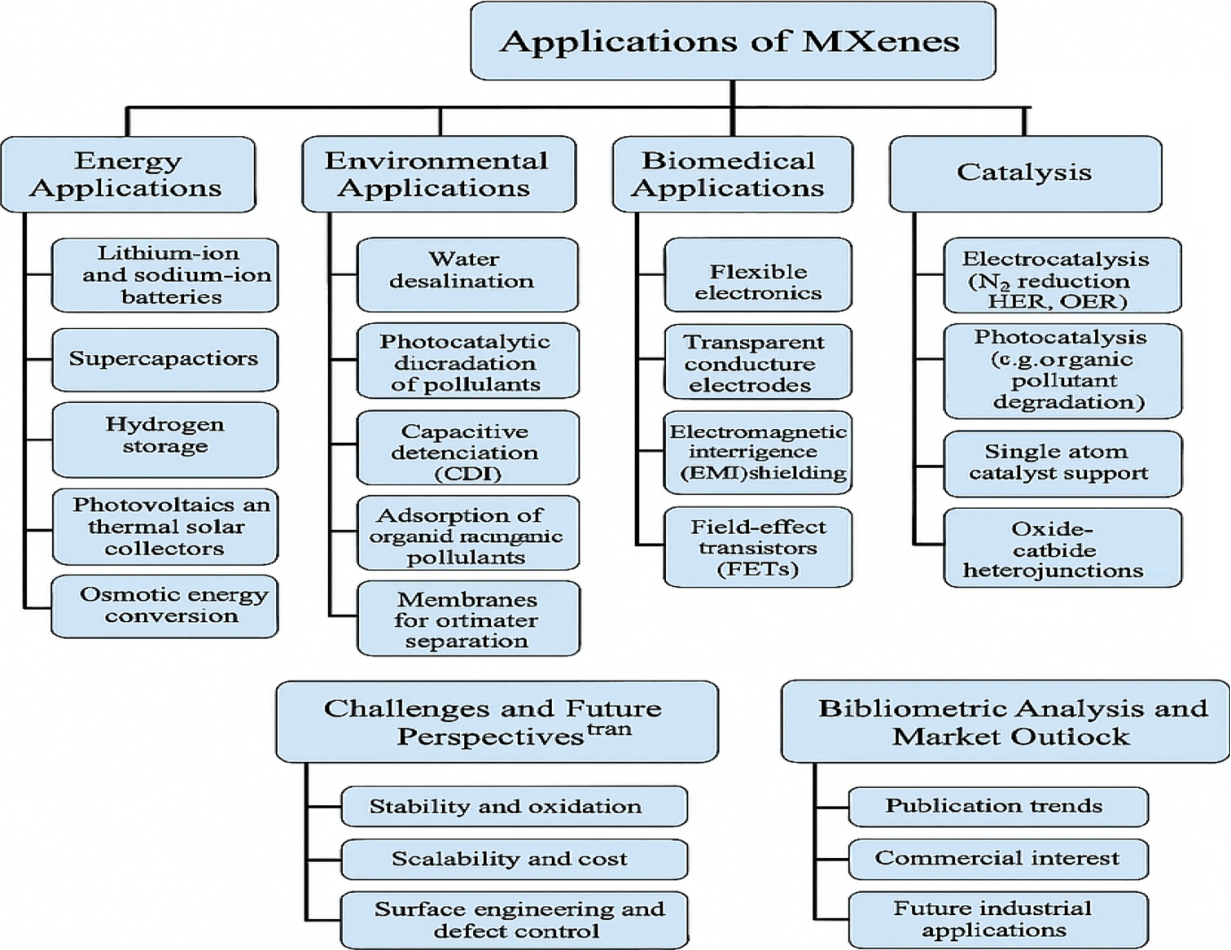
Flowchart summarizing the diverse applications of MXenes across five major domains: energy, environment, biomedicine, sensors and electronics, and catalysis. It also highlights challenges, future perspectives, and market trends relevant to MXene development.

Because of their remarkable conductivity, tunable surface chemistry, and large surface area, MXenes—a more recent generation of two-dimensional transition metal carbides, nitrides, and carbonitrides—have rapidly emerged as outstanding possibilities for a wide range of applications. In the energy area, they are investigated as a candidate material for batteries, supercapacitors, and hydrogen evolution catalysis, in which their conductivity and high abundance of active sites facilitate efficient charge transport and catalysis kinetics. Their hydrophilicity and surface functional groups also qualify them as an efficient candidate for water purification and environmental cleanup. MXenes also exhibit promise in wound healing and drug delivery and biosensing in biomedical applications. They are also shown to possess optical and electromagnetic functions to serve as a sensor and as a component for field-effect transistors and EMI shielding. These multifunctional features are outlined in (Fig. 16) and represent an overall chart of MXene applications in the energy, environmental, biomedical, and electronic fields.

### 4.1. Applications in energy storage systems

MXenes are excellent candidates for the creation of high-performance energy storage devices like batteries and supercapacitors because of their layered 2D structure, high electrical conductivity, adjustable surface chemistries, and huge surface area. Their metal conductivity as high as 10 000 S cm^−1^ enables fast electron mobility, while interlayer spacing diversity enables efficient ion intercalation and deintercalation—critical to enhance the performance of batteries and cycling stability. Functionalized surface groups (–O, –OH, –F) also impart a contribution to the pseudocapacitive response and redox activity. Composite structures like Ti_3_C_2_T_*x*_@graphene and WS_2_@Ti_3_C_2_T_*x*_ enhance efficient ionic transport and prevent stacking. MXene is a perfect material because of the following features: lithium/sodium-ion batteries and supercapacitors. Some MXene composition and their corresponding energy storage applications are illustrated in (Table 6).

**Table 6:** Summary of MXene types, compositions, key properties, applications, and references

MXene type	Composition	Applications/key properties	References
Ti_3_C_2_T_*x*_	Titanium carbide	High conductivity, hydrophilicity, energy storage (batteries, supercapacitors), anticorrosion coatings	175 and 176
Ti_2_C_2_T_*x*_	Titanium carbide	Lightweight, thinner structure, enhanced ion transport, compact energy storage	177
Nb_2_C_*x*_T_*x*_	Niobium carbide	Electrochemical properties, used in batteries and supercapacitors	178
Nb_4_C_3_T_*x*_	Niobium carbide	Advanced electrochemical properties for high-energy storage	179
Mo_2_C_*x*_T_*x*_	Molybdenum carbide	Catalytic applications, robust mechanical stability, energy storage	180
V_2_C_*x*_T_*x*_	Vanadium carbide	Unique electrochemical properties, used in supercapacitors and batteries	181
V_4_C_3_T_*x*_	Vanadium carbide	Similar to V_2_C_*x*_T_*x*_, with applications in complex electrochemical setups	182
Zr_3_C_2_T_*x*_	Zirconium carbide	Structural materials, electronic applications	183
Hf_3_C_2_T_*x*_	Hafnium carbide	Specialized high-performance applications requiring robustness	184
Ti_3_CNT_*x*_	Titanium carbonitride	Combines carbide and nitride properties for catalysis and advanced devices	185
Ti_3_C_2_T_*x*_@MoS_2_	Titanium carbide + MoS_2_	Hybrid MXene for enhanced catalytic performance and corrosion resistance properties	186
WS_2_@Ti_3_C_2_T_*x*_	Titanium carbide + WS_2_	Improved chemical stability, enhanced corrosion resistance	187
Ti_3_C_2_T_*x*_@graphene	Titanium carbide + graphene	Prevents restacking, improves ion transport, and achieves high electrochemical performance	188
N-doped Ti_3_C_2_T_*x*_	Nitrogen-doped MXene	Boosts conductivity and redox performance for flexible, printed energy storage devices	145
Sulfur-decorated Ti_3_C_2_T_*x*_	Sulfur-modified MXene	Designed for specific battery applications, including sodium-ion batteries	189
Mo_2_TiC_2_T_*x*_	Double transition metal MXene	Enhanced structural and electrochemical diversity for advanced applications	190
Mo_2_Ti_2_C_3_T_*x*_	Double transition metal MXene	Complex composition for high stability and tailored applications	191
Cr_2_CT_*x*_	Chromium carbide	Applications in catalysis and energy storage systems	59
Ta_4_C_3_T_*x*_	Tantalum carbide	High robustness and structural diversity for specific applications	192

Supercapacitors are a subclass of electrochemical capacitors, which are devices used to store energy. Between batteries and traditional capacitors, they bridge the gap. High power density, quick charge–discharge rates, and superior cycling stability are all hallmarks of supercapacitors. Unlike batteries, supercapacitors store energy electrostatically by either accumulating ions on the electrode surface (electric double-layer capacitance) or creating pseudocapacitance at the electrode surface through rapid and reversible redox reactions.

**Fig. 17:**
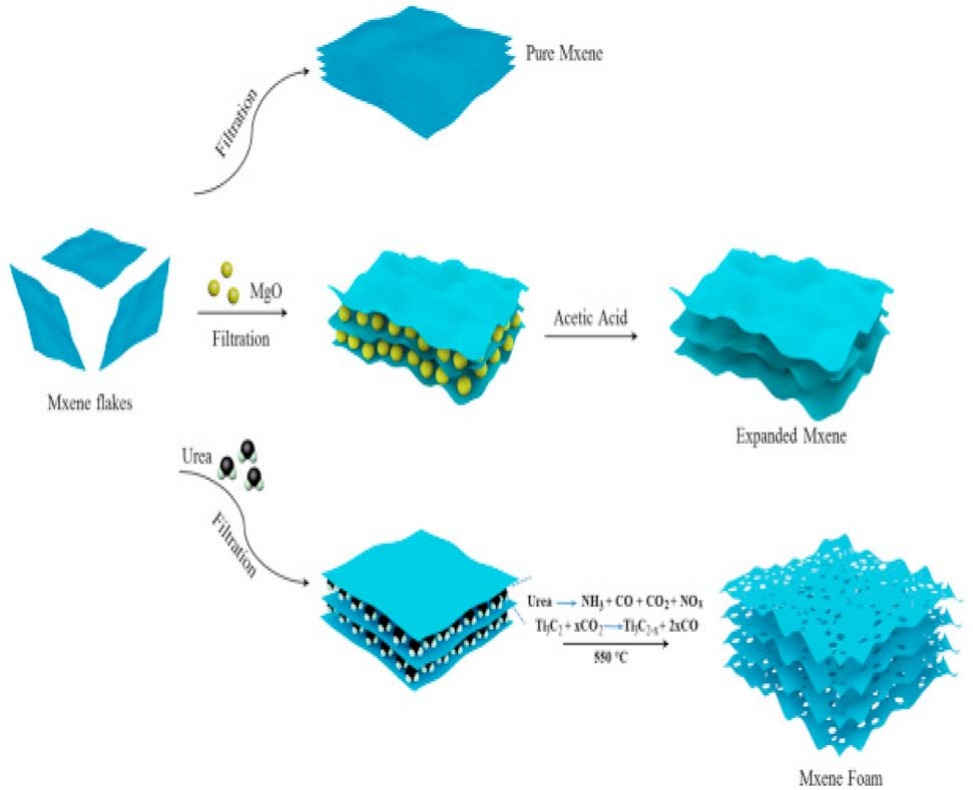
Fabrication and structural modification of MXene-based materials for supercapacitor applications: schematic representation of the fabrication and structural modification of MXene-based materials for supercapacitor applications. Starting from MXene flakes, pure MXene is obtained by filtration. Interlayer spacing is expanded by incorporating and subsequently removing MgO using acetic acid, producing expanded MXene. Further treatment with urea and thermal processing leads to the formation of porous MXene foam, enhancing ion transport and surface accessibility for improved supercapacitor performance reproduced with permission from ref. 192, Elsevier, Copyright 2020.

These features make supercapacitors ideal in systems requiring quick bursts of energy such as hybrid vehicles, renewable energy systems, and portable electronics. MXenes are a very promising family of electrode materials for supercapacitors, with excellent specific capacitance, rapid ion intercalation, and pseudocapacitive behavior. For example, the volumetric capacitance for the class of Ti_3_C_2_T_*x*_ MXene is manyfold higher compared to that from typical carbon materials represented by graphene and carbon nanotubes. Besides, in hybrid nanocomposites with conducting polymers or metal oxides, the improvement of conductivity, preventing the restacking of the layers, further promotes superior electrochemical performance. Thin and flexible films of MXene enable several other applications in micro supercapacitors and, further, wearable energy storages since MXenes can easily meet most such requirements. These advantages are a great asset, but individual pure MXenes present very critical issues of restacking layers from a two-dimensional planar structure that significantly diminish the effective surface area accessible, likely causing ion transportation restrictions. Hence, modification, structural engineering, or simply tailoring MXene samples to enhance performances accordingly for supercapacitive applications has attracted a strong emphasis of effort so far. As shown in (Fig. 17) (Zhu *et al.*,^192^ 2020), expanded MXenes and MXene foams will be designed by strategic modification. Pure MXene flakes are prone to stacking due to the van der Waals attraction; thus, the preparation is initiated with pure MXene flakes. Introducing MgO nanoparticles as spacers and their subsequent removal by acetic acid increase the interlayer spacing of MXenes. Such modification will enhance ion diffusion pathways and improve electrolyte accessibility, hence increasing the specific capacitance. Apart from the above, the urea thermal treatment forms MXene foams with a macro-porous structure and a three-dimensional framework of cross-linked channels. This unique architecture allows fast ion transport and prevents layer restacking, further enhancing the electrochemical performance. These structural modifications significantly enhance the specific capacitance, ion intercalation rates, and pseudocapacitive behavior of MXenes. Accordingly, the volumetric capacitance of Ti_3_C_2_T_*x*_ MXene thus outperforms their previously thought volumetric capacitances over traditional carbon-based alternatives such as graphene or nanotubes. Besides hybridizing the MXenes composited with conducting polymers/metal oxides provides greater conductivity and stability upon considerations that are considered very good at high performance for these storages of energy. The thin and flexible MXene films lend themselves to applications in either micro-supercapacitors or wearable energy storage devices, further demonstrating the versatile and promising use of MXenes in next-generation energy systems.

#### 4.1.1. Lithium-ion batteries (LIBs)

LIBs are a type of rechargeable energy storage device that acts based on the principle of lithium ion movement between anodes and cathodes during their charge/discharge cycling. With their high energy density, lightweight, and long-life features, LIBs are becoming indispensable in many areas of application, including portable electronics, electric vehicles, and renewable energy systems. The anode is usually of graphite or silicon and serves like a reservoir for lithium ions during charging, while at the cathode, it releases them, creating an electron flow that powers up devices. The layered nature of the MXene allows fast electron transfer and ion diffusion. Because of these properties, within the LIB frame, MXene is one of the widely used electrode materials. Used together with active materials like silicon and metal oxides, it has been seen that MXenes can overcome certain difficulties-for example, the volume expansion of electrodes-allowing a high specific capacity to be attained, along with prolonged cycling stability.

**Fig. 18:**
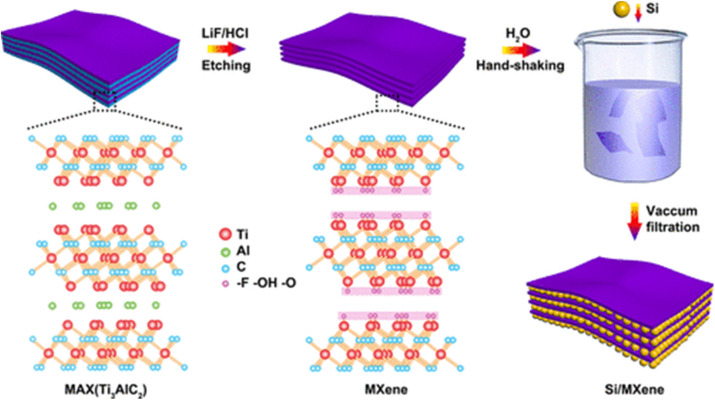
Schematic diagram for the preparation of flexible and freestanding Si/MXene composite paper for lithium-ion batteries: the process begins with selective etching of Al from the MAX phase (Ti_3_AlC_2_) using a LiF/HCl solution, yielding multilayered MXene (Ti_3_C_2_T_*x*_). Subsequent delamination through hand-shaking in water results in few-layer MXene sheets. These are then mixed with silicon (Si) nanoparticles and subjected to vacuum-assisted filtration, producing a layered Si/MXene composite with high flexibility, improved conductivity, and enhanced mechanical integrity for use as a high-performance anode material reproduced with permission from ref. 194, Copyright 2025, Elsevier.

As shown in (Fig. 18) (Tian *et al.*, 2019),^193^ the Schematic illustration of the preparation procedure of flexible and freestanding Si/MXene composite paper applied as an advanced anode in LIBs. Firstly, the synthesis of the Ti_3_C_2_ MXene is conducted by the etching of aluminum from the Ti_3_AlC_2_ MAX phase with a LiF/HCl solution, and then by hand-shaking with water to delaminate the MXene sheets into a colloidal suspension. This results in highly conductive and flexible MXene sheets with a two-dimensional layered structure. Silicon (Si) nanoparticles are then integrated into the MXene solution, forming a homogeneous mixture.

Subsequently, the Si/MXene composite is prepared, by which the silicon nanoparticles undergo covalent anchorage through vacuum-assisted filtration onto the MXene sheet. Such a structure has several advantages in LIBs: first, the layered MXene allowed fast electron transfer and ion diffusion; second, the incorporation of Si brings more active sites and avoided the restacking of MXene; and third, the flexibility of the composite can tolerate the volume variation of silicon during the cycles of lithiation/delithiation.

The merits of these features for Si-based anodes include high specific capacity, improved cycling stability, and long lifetime in LIB applications, addressing some of the fundamental bottlenecks, such as poor conductivity and significant volume changes of traditional Si anodes.^193^

#### 4.1.2. Sodium-ion batteries (SIBs)

Ion transport and storage capacity are enhanced by sodium ion intercalation, which is supported by the wide interlayer spacing in MXenes. Optimizing ion diffusion pathways with varied surface chemistries enhances reversible capacity. Composites made of other materials help to mitigate the issues of volume expansion and dendrite growth, ensuring better cycling performance and stability.

#### 4.1.3. Lithium–sulfur batteries (LSBs)

**Fig. 19:**
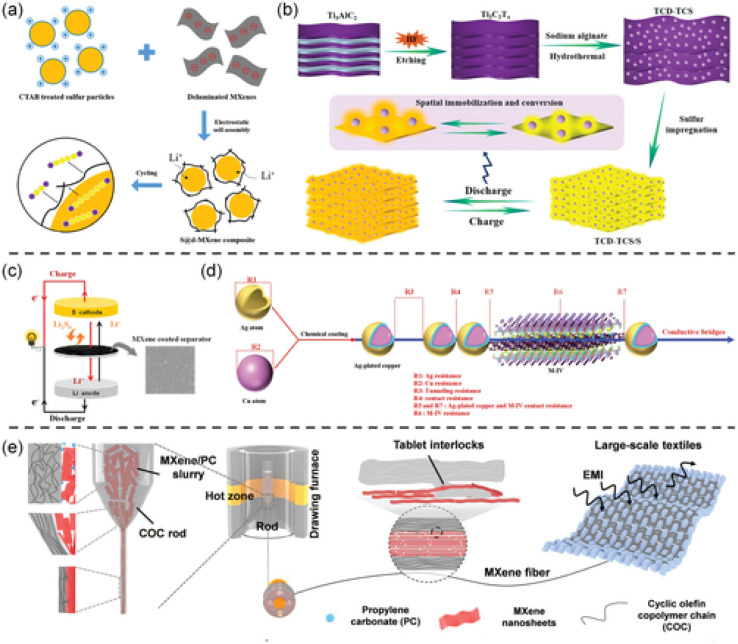
Comprehensive applications of MXenes in lithium–sulfur battery design and optimization: comprehensive schematic illustrating the multifunctional applications of MXenes in lithium–sulfur (Li–S) battery design and optimization. (a) MXenes act as conductive hosts for sulfur particles, enhancing cycling stability and mitigating polysulfide shuttling. (b) Their incorporation into sulfur composites helps spatially confine and convert polysulfides during charge/discharge cycles. (c) MXene-coated separators provide improved ionic conductivity and suppressed polysulfide diffusion. (d) As conductive interlayers on current collectors, MXenes facilitate redox kinetics and minimize resistance. (e) MXene-based fibers integrated into large-scale textiles show potential for flexible and wearable energy storage with enhanced mechanical and electromagnetic interference (EMI) shielding properties reproduced from ref. 196, Copyright CC BY 3.0, 2024. Wiley.

With their strong polysulfide adsorption capabilities, MXenes have been acting as sulfur hosts that address the shuttle effect, thus enhancing cycling stability. High conductivity in them accelerates redox kinetics, while structural diversity can adapt to volume changes in the charge and discharge cycles. As shown in (Fig. 19) (Tian *et al.*,^195^ 2022), MXenes have been developed in multiple ways for LSBs, including acting as a host for sulfur and helping with overall battery performance. With intrinsically high conductivity and strong polysulfide adsorption, MXenes have indeed successfully eliminated one of the major problems of LSBs, which is known as the ‘shuttle effect’, a problem arising due to dissolution and migration of lithium polysulfides. Serving as sulfur hosts, MXenes provide a robust platform that physically traps and chemically interacts with polysulfides, reducing loss during cycling and thus enhancing the stability of the battery. Fig. 19 emphasizes some of the big hurdles that need to be overcome in LSBs, which have been addressed in the design of MXene-based materials. For example, sulfur encapsulated in delaminated MXene (S@d-MXene) composites not only suppresses the dissolution of polysulfide but also accommodates volumetric expansion during charge–discharge cycles. In addition, as schematically illustrated, the use of MXene-coated separators and conductive networks enhances redox kinetics because of their high electrical conductivity and reduced charge-transfer resistance. The structural diversity of MXenes allows flexibility in adapting to the volumetric changes of sulfur, hence improving cycling stability and capacity retention. The value addition of MXenes has been due to their versatility in structure, conductivity, and polysulfide trapping ability, hence opening new frontiers in developing high-performance LSBs. As the figure shows, scalability of MXene fibers and conducting media points toward next-generation battery applications requiring robust design and improvement in electrochemical performances.^196^

### 4.2. Biomedical applications of MXenes

MXenes are most frequently used in biomedical applications for wound healing and haemostasis based on polysaccharides. Haemostasis, inflammation, proliferation, and tissue repair and remodelling are the four primary phases of wound healing, which is a complex biological process.^197^ Haemostasis and antibacterial activity are extremely important during these phases. Haemostasis, the initial stage of wound healing, is the process by which the wound closes through clotting and occurs within a few seconds of the blood vessel's epithelial wall being injured.^198^ Hemostasis is the mechanism of the bleeding cessation from the blood vessels; the platelets were aggregated and adhered to the endothelium surface of the ruptured blood vessel forming a plug to close the damaged part of the blood vessel preventing the bleeding. The effectiveness of the hemostasis is not only to prevent the ruinous effect of the bleeding but also to provide a stabilized microenvironment for the sequential inflammatory and remodeling responses.^199,200^

**Fig. 20:**
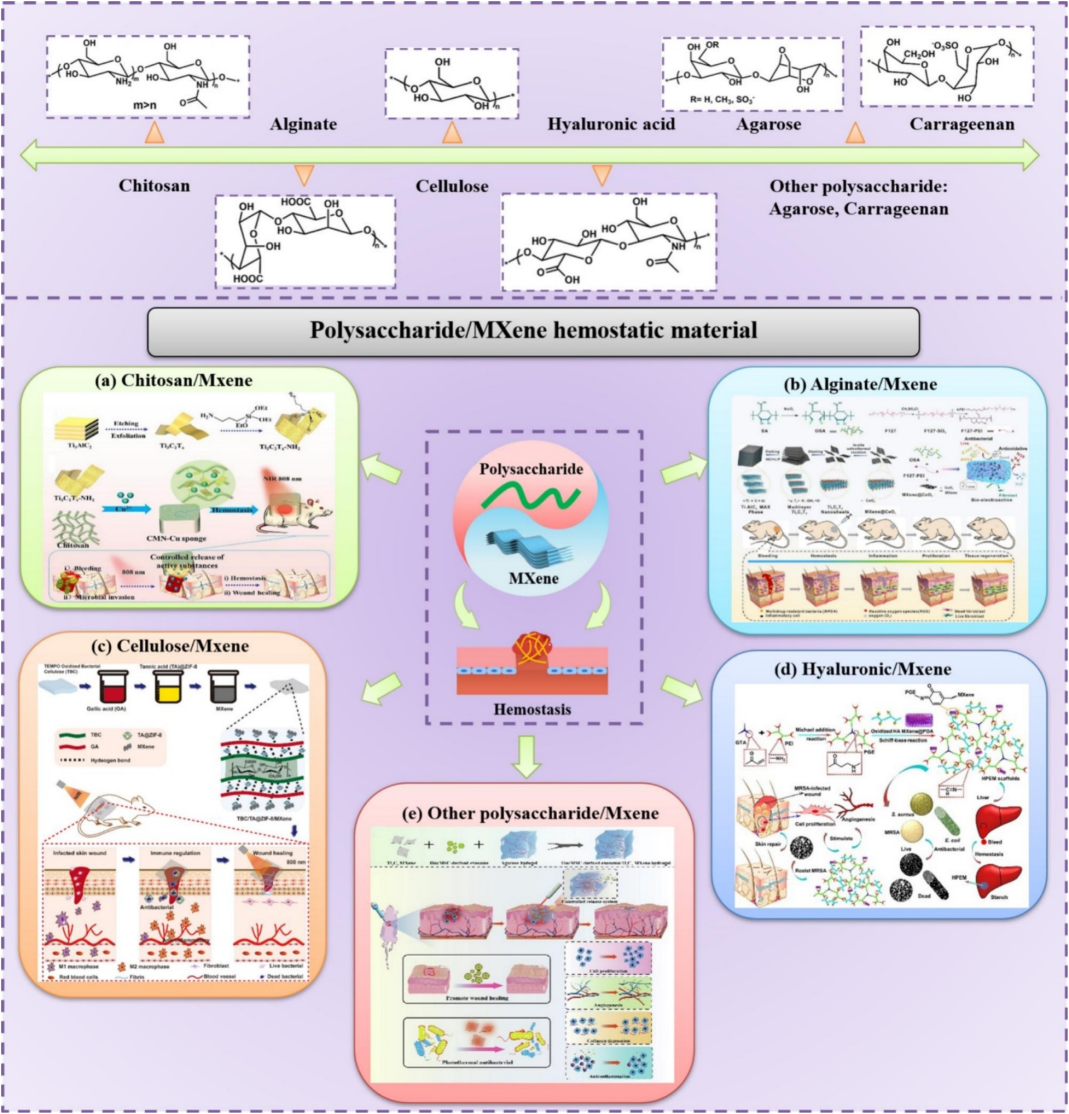
Different polysaccharide and polysaccharide/MXene composites structures, for wound healing and hemostasis. (a) Chitosan/MXene material, (b) alginate/MXene, (c) cellulose/MXene material, (d) hyaluronic/MXene; (e) other polysaccharide/MXene. Reproduced with permission from ref. 210, Copyright, 2025, Elsevier.

**Fig. 21:**
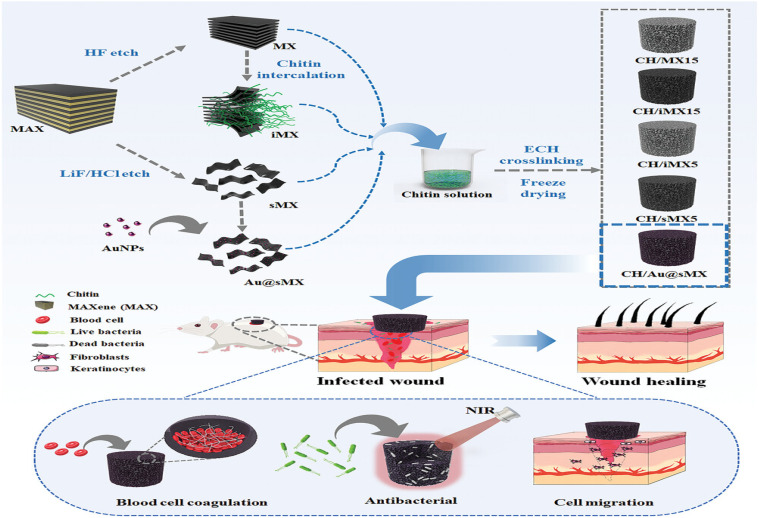
MXene-enhanced chitin composite sponges for wound healing with antibacterial and hemostatic properties reproduced from ref. 211, Copyright CC BY 3.0, 2022 Wiley.

In addition, it promotes the promptly wound closure to minimize risk of infection from external pathogens exposure, accordingly accelerating and enhancing the wound healing process. Chitosan, xanthan, cellulose, hyaluronic acid, sodium alginate and other polysaccharide-based biopolymers can be used widely as hemostasis materials owing to their distinguished biocompatibility and designability features.^201–206^ However, their low antimicrobial potency and insufficient hemostatic capabilities limited their applications in the wound care strategies. MXene represents an emerging promising two-dimensional nanomaterial constructed from transition metal carbonitriles, carbides or nitrides represented by the formula M_*n*+1_X_*n*_T_*x*_. MXenes revealed high surface area and biocompatibility. Additionally, they exhibit excellent conductivity and hydrophilicity near the infrared responsiveness. MXenes possess a significant antimicrobial activity. Furthermore, their large surface can enhance the hemostasis by concentrating the clotting factors and increasing the moisture absorption. Consequently, MXenes can offer brilliant solutions to the limitations of the other polysaccharide-based biomaterials (chitosan, cellulose, *etc.*) in wound care. In recent years, many researchers in the medical field have emphasized the potential of MXenes in the wound care applications. Their methods of preparation and medical applications have generated a remarkable attention. However, MXenes have some limitations.^207^ For instance, easy oxidation and poor adhesion capability. To address their shortcomings and enhance their performance, MXenes–polysaccharide nanomaterials have been developed, included MXene/sodium alginate^205^ MXene/cellulose^208^ MXene/chitosan^209^*etc.* (Fig. 20 and 21). To substantiate these claims with quantitative benchmarks, representative MXene–polysaccharide dressings achieve rapid hemostasis in small-animal trauma models: bleeding from mouse liver injury is arrested within ∼26.5–48 s, and rabbit ear vein rupture within ∼9.1 s. In parallel, infected-wound healing accelerates markedly—*e.g.*, 84% closure by day 9, ∼96% by day 14, and complete closure reported within 11–14 days depending on the system and infection status. On the antibacterial side, MXene-based composites (often combined with photothermal/biocidal components such as lysozyme or peptides) suppress MRSA effectively (*e.g.*, ∼90% healing by day 7; full closure by day 12) and exhibit efficient photothermal conversion. Where studies quantified antibacterial action *via* zone-of-inhibition assays against *S. aureus* or *E. coli*, clear inhibition was observed; details of the specific protocols and values are summarized in the cited review.

#### 4.2.1. MXenes and photo thermal therapy (PTT)

MXene has a photothermal efficacy, it can convert laser light energy into heat power. Scientists have therefore studied MXenes for the PTT of cancer, which uses heat to kill malignant tumors, causing protein denaturation and ultimately cell death.^212–218^ A novel treatment that can overcome the limitations of chemotherapy or surgery is photothermal therapy (PTT), which generates heat under irradiation to achieve photothermal ablation of breast cancer with minimal invasiveness and exceptional spatial-temporal selectivity. PTT is one of the most effective and dependable cancer therapy techniques since it has been shown to decrease tumors by triggering cell death through the apoptosis internal pathway.^219^

Reactive oxygen species are produced by MXene to affect live cells biologically. MXenes' anti-cancer potential stems from the increased baseline level of reactive oxygen species in cancer cells, which is caused by improved anabolic and catabolic activities.^220^

While A375 (malignant melanoma cell of skin) and A549 (human alveolar basal epithelial cell) were used as cancerous cell lines, A. M. Jastrzębska *et al.* used MRC-5 normal lung cells and HaCaT normal skin cells to investigate the biological activity of MXenes *in vitro* on both normal and cancerous cell lines. MXenes had a greater cytotoxicity to cancerous cell lines. The primary mechanism for cytotoxicity was the production of “Reactive Oxygen species (ROS) as MXenes affect oxidative stress”.^221^

The A 459 cell lines showed the highest amounts of cytotoxicity after being incubated with MXenes. Drug delivery is one of the many medical applications for MXenes^222,223^ biomedicine, cancer treatment,^224^ anti-bacterial,^215,225^ and diagnosis.^226^

For instance, Hussein and associates created plasmonic-based nano composites Au/Fe_3_O_4_/Ti_3_C_2_ and Au/Ti_3_C_2_ that have anticancer properties and are less harmful *in vivo* than Ti_3_C_2_. Therefore, compared to MXene alone, Au/Fe_3_O_4_/MXene and AU/MXene showed decreased foetal murrain. Additionally, Au/Fe_3_O_4_/MXene and Au/MXene's cellular photothermal transformation capabilities were evaluated using the MCF7 breast cancer cell line. The relative vitality of the cell without and with laser treatment was assessed after incubation (using different concentrations of nanocomposites). There was no discernible cytotoxicity for “laser-free”.^227^

#### 4.2.2. Biosensors based on MXenes

An unlabeled, highly sensitive electrochemical biosensor based on Ti_3_C_2_ nanosheets was developed by Kumar *et al.* for the identification of carcinoembryonic antigen (CEA). Following that, APTES was used to functionalize Ti_3_C_2_ nanosheets for antiCEA covalent stabilization. An illustration of how the redox probe interacts with the electrode surface. A broad detection range is demonstrated by the biosensor (BSA/anti-CEA/f-Ti_3_C_2_-MXene/GCE) that was designed.^228^

To find MUC1 (Mucin1) as a marker for breast cancer, Wang *et al.* developed a cDNA-Fc/MXene probe based on a competitive electrochemical biosensor in a different investigation.^213^ The transmembrane glycoprotein MUC1 is of interest for detection because of its abnormal expression in tumour tissues.^39^ To improve diagnostic signals and provide a high number of binding sites for cDNA-Fc, MXene was used as a cDNA-Fc nano carrier. Ti_3_C_2_–IONPs–SPs demonstrate remarkable photothermal conversion efficiencies (48.6%) to shrink tumor tissues and eliminate cancer cells in both *in vitro* and *in vivo* conditions.

#### 4.2.3. MXenes as drug delivery systems

In the context of healthcare and the expanding field of medical sciences, the development of efficient drug delivery systems holds significant promise for the treatment of a variety of disorders. Drug delivery systems have made significant strides, but several issues still exist, requiring additional improvements to maximize patient outcomes. Examples of smart nano-carriers include 2D sheets. The recently developed nano-sheets known as nano-carriers may attract interest for the targeted administration of medications, genes, and bioactive substances that destroy cancer cells. Ti_3_C_2_T_*x*_-MXene has emerged as a notable intelligent nano-carrier in nanomedicine because of these developments. Its remarkable qualities made it the perfect nano-carrier for cancer treatment. Ti_3_C_2_T_*x*_-MXene 2D nano carriers have been developed in recent drug delivery research to release pharmaceuticals in response to stimuli, guided by unique physicochemical properties.^229^

MXenes have a vast surface area that can be used as a carrier for freight delivery because of their naturally flat nature. In the meantime, surface engineering creates multifunctional Nano platforms by combining various therapeutic agents to produce synergistic therapy.^230^

One essential and crucial step in the treatment of cancer is chemotherapy. In addition to killing cancerous cells, cancer drugs also cause horrible side effects in people.

Thus, it is necessary to create drug delivery systems that can target drug delivery and focus more on cancer tissues. Ti_3_C_2_ MXene nanosheets are thought to be promising drug delivery agents because of their distinct structure and adjustable functionalization.^231^

As an example, the anti-cancer medication doxorubicin (Dox) was conjugated onto the surface of electrostatic adsorption of Ti_3_C_2_. The Dox release was found to be significantly better. MXenes' increased surface area and numerous functional groups allow them to bind a wide variety of medicines for tailored medication delivery.^232^ Al^3+^ ultrathin Ti_3_C_2_ MXene nanosheets (100) were also tested for cancer treatment in another study; they demonstrated an effective single oxygen production (^1^O_2_) after laser irradiation at 808 nm, a high mass extinction coefficient (28.6 L g^−1^ cm^−1^ at 808 nm), and a high photothermal conversion efficiency (about 558.3 percent).^233^ Another study reported using MXene (Ti_3_C_2_) as a PTT platform to cure cancer. Studies conducted both *in vitro* and *in vivo* have demonstrated that MXene nanosheets coated with soybean phospholipid and a phase-changeable organic–inorganic hybrid (PLGA/Ti_3_C_2_) have demonstrated effective photo-response and strong biocompatibility in killing cancer cells.^232^ Li *et al.*^161^ also demonstrated MXene nanosheets' exceptional light-to-heat conversion capabilities.

#### 4.2.4. MXenes as an anti-bacterial agent

The death rate among infected individuals is rising due to multidrug-resistant bacteria, which is a global concern.^234^ Globally, antibiotic-resistant bacteria cause around 7 million deaths annually; if appropriate treatments are not developed, this number is predicted to soar to 10 million by 2050.^235^

Because of its antibacterial properties, which include physical damage, oxidative stress, and photothermal and photodynamic therapy, MXene as a nanoparticle can be an effective treatment.^236^

Various antibacterial techniques, such as nanothermal blades, ROS generators, and nanoknives, work against different microorganisms. Both Gram-positive and Gram-negative bacteria, such as *Bacillus subtilis*, *E. coli*, and *Staphylococcus aureus*. Because of their sharper edges, the thinner MXenes inhibited *Escherichia coli*, *Sarcina*, *K. pneumoniae*, *Pseudomonas aeruginosa*, *S. typhi*, and faecalis more successfully.^237^

According to studies, 2D Ti_3_C_2_T_*x*_ nanosheets' sharp edges serve like nano knifes, destroying the bacteria's outer cell walls. When bacteria encountered MXene flakes, the sharp edges of the Ti_3_C_2_T_*x*_ nanosheets severely damaged their membranes and caused cytoplasm to spill out. The sharp edges of the MXenes easily pierce and cut the cell membrane, which has a thickness of 20 to 50 nm. Smaller nanosheets may physically penetrate bacteria or enter them directly through endocytosis.^237^ The DNA of the bacteria is cleaved by the nanosheets that have entered the cell. Other factors, such as the high hydrophilic and negative character of Ti_3_C_2_T_*x*_'s antibacterial activity, are also essential for the blade's antibacterial activity in addition to its sharp edge. MXene has been shown to have concentration-dependent antibacterial activity. It also boosts the direct death rate of bacteria by inactivating them and forming an H-bond between its oxygenate groups and the lipopolysaccharide cords on the bacterial cell layer, which prevents the bacteria from feeding. MXene's efficacy against microbial species was raised to almost 95%.^238^

According to other research, MXenes cause oxidative stress and ROS, which are chemically reactive unpaired electrons of oxygen molecules that have the ability to destroy microbes and cancer cells.^239,240^

Because of their rich electrical properties, oxidative stress and ROS formation have been thought to be further pathways for upsetting the bacteria in MXenes.^241,242^

Other studies showed that MXenes have a photo-thermal activity against bacteria. It kills bacteria by photothermal means when exposed to light.^243^

The heat generated might seriously damage the bacterial cell membrane. Membrane permeability and intracellular homeostasis are both affected by damaged membranes. Denaturation of DNA and proteins is another direct impact of heat that may result in bacterial inactivation.^244^ MXenes' cutting edges to rupture the bacterial barrier.^245,246^ According to another study, Ti_3_C_2_T_*x*_-modified membranes' bactericidal qualities were evaluated against *B. subtilis* and *E. coli*. When compared to control PVDF, the antibacterial rate of fresh Ti_3_C_2_T_*x*_ MXene membranes is over 73% against *B. subtilis* and 67% against *E. coli*. In contrast, aged Ti_3_C_2_T_*x*_ membranes demonstrated over 99% growth suppression of both bacteria under the same conditions.^247,248^ Rasool *et al.* initially examined the antibacterial qualities of Ti_3_C_2_T_*x*_ MXene and used SEM and TEM to observe the alterations in bacterial morphology and membrane integrity following interaction with *E. coli* and *B. subtilis*.^215^ The experimental group displayed membrane damage and cytoplasmic leakage, while the control group displayed neither cell death nor membrane damage, according to SEM pictures. All the bacteria experienced widespread cell lysis, which was characterized by significant membrane rupture and cytoplasmic leaking, when the concentration of Ti_3_C_2_T_*x*_ was raised to 100 µg mL^−1^. According to the TEM images, the experimental group of bacteria lost intracellular material because the Ti_3_C_2_T_*x*_ nanosheets were heavily adsorbed around and even pierced the cells. Furthermore, the bacteria's overall intracellular density reduced. TEM analysis was used by Pandey *et al.*,^241^ following ultrathin bacterial sectioning. They found that Nb_2_C_2_T_*x*_ and Nb_4_C_3_T_*x*_ nanosheets were absorbed by the cell walls of *S. aureus* and *E. coli*. A few of the nanosheets managed to penetrate the cell walls, causing cytoplasmic membrane and cell wall breakage and pore development, cellular contents to leak out, and severe intracellular structural distortion and damage. The impact of Ti_3_C_2_T_*x*_ nanosheets of different diameters on the development of *B. subtilis* and *E. coli* strains was investigated by Arabi Shamsabadi *et al.* According to their growth kinetics experiments, the bacteria were harmed by the sharp edges of the nanosheets because they physically interacted with their membrane surface, causing the bacterial envelope to break down and cytoplasmic DNA to leak out.^249^ The effectiveness of MXene as an antibacterial agent was investigated by Yu *et al.*,^250^ who found that the ROS produced by the MXene group was 1.8 times greater than that of the control group. They then used a lipid peroxidation assay to assess whether the ROS damaged the bacteria's membranes. They found that the MXene group had a 1.3-fold greater capacity to oxidize the bacterial membranes than the control group. This implies that MXene may cause oxidative stress, which can damage bacterial membranes, by generating ROS. The antibacterial properties of single-layer and few-layer Ti_3_C_2_T_*x*_ MXene flakes in colloidal solution were investigated in another investigation. Bacterial growth curves based on optical densities (OD) and colony growth on agar nutritive plates against *Escherichia coli* (*E. coli*) and *Bacillus subtilis* (*B. subtilis*) were used to assess Ti_3_C_2_T_*x*_'s antibacterial properties. It has been widely documented that graphene oxide (GO) is not as effective as Ti_3_C_2_T_*x*_ as an antibacterial agent against Gram-positive *B. subtilis* and Gram-negative *E. coli*.^215^

### 4.3. Environmental applications of MXenes

Because of their high surface area, modulable surface groups, and high adsorption capability, MXenes have demonstrated exceptional promise in environmental remediation.^251–255^ Their negatively charged features facilitate discriminatory removal of heavy metal contaminants and organic dyes by their modulated efficiency by means of surface modification by means of alkali treatments. Moreover, their photothermal and photocatalytic capabilities also promote the decomposition of contaminants by the generation of reactive oxygen species. These properties make MXenes efficient materials in water purification, gas cleaning and purification of air and water, and sensing of the environments. The widespread industrialization and urbanization globally have resulted in serious environmental issues such as water pollution, air pollution, and buildup of toxic substances. MXenes were found to be advanced materials on account of their large surface area, numerous functional groups, good hydrophilicity, and tunable surface chemistry to solve all such issues. Their distinctive physicochemical nature allows efficient adsorption, photocatalysis, electrocatalysis, and sensing of a vast variety of environmental contaminants. Recent advances in the use of MXenes as effective environmental remediation agents are covered in the section that follows. These developments are based on the compounds' effectiveness in removing organic pollutants and heavy metals, extracting radionuclides, sensing gases, desalinating seawater, and detecting pesticides. The discussions involve both experimental and theoretical perspectives on account of the vast scope of MXene in providing efficient and sustainable solutions to environmental issues.

#### 4.3.1. Water treatment

##### 4.3.1.1. Heavy metal removal

**Fig. 22:**
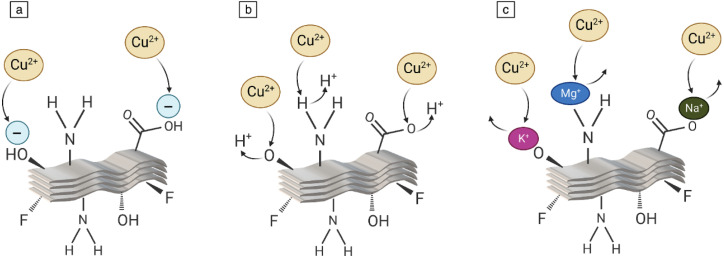
Adsorption modes of MXenes. Schematic illustration of the mechanisms by which MXene materials remove Cu^2+^ ions from aqueous solutions, including (a) electrostatic attraction, (b) surface complexation through functional groups, and (c) ion exchange with pre-adsorbed cations such as Na^+^, K^+^, and Mg^2+^. Reproduced from ref. 254, Copyright 2023, Springer.

MXenes demonstrate outstanding ability for heavy metals adsorption, like chromium (Cr^6+^), mercury (Hg^2+^), and lead (Pb^2+^). Their rich surface functional groups enable efficient ion exchange and chemisorption processes, resulting in remarkable removal performance. As described in (Fig. 22). Because of their large specific surface area, abundance of functional groups, and adjustable surface chemistry, MXenes have adsorption activity that is comparable to or even greater than that of other nanomaterial adsorbents. The diverse functional groups on the MXene surface provide active sites for surface complexation and ion exchange with heavy metals, while also enabling the reduction of certain metals.^256–258^

Alyasi *et al.* (2025) synthesized a composite material by integrating magnetic MXene (Ti_3_C_2_T_*x*_), chitosan–lignosulfonate (CLS) nanobeads, and delaminated Ti_3_C_2_T_*x*_ (DL-Ti_3_C_2_T_*x*_) using a simple route involving integration of Fe_3_O_4_. The synthesized material was constructed to achieve the specific adsorption effect of MXene, the magnetically separate property of Fe_3_O_4_, and the biocompatibility of CLS. The composite material was first tested to adsorb Cr(vi) from a batch system with a removal efficiency of 90% and an adsorption capacity of 42.5 mg g^−1^. The adsorption was found to comply with the pseudo-second-order kinetics model and was found to fit the Langmuir isotherm showing adsorption to be a monolayer process. The findings indicated that the primary adsorption mechanisms involved electrostatic interactions, complexation, surface intercalation, and the reduction of toxic Cr(vi) to Cr(iii) on the prepared material, also, the obtained data demonstrated different levels of attraction for various metal ions, with the removal efficiency following this order: Cr(vi) > Ni(ii) > Cu(ii) ≈ Co(ii).^258^

Luo *et al.* (2025) prepared a new MXene material, TiVCT_*x*_, by *in situ* etching. They explored the adsorption of the heavy metal anion Cr(vi) by it in terms of adsorption time, pH values, effect of temperature, initial adsorbent and adsorbate concentrations, and impact of interference ions on Cr(vi) adsorption. The resultant data show remarkable Cr(vi) adsorption capacity by TiVCT_*x*_ to a maximum adsorption of 600 mg g^−1^. The adsorption capacity is substantially better compared to other MXene materials. Moreover, the adsorption of Cr(vi) by TiVCT_*x*_ attains the adsorption equilibrium in a very short time of 10 minutes, reflecting its high adsorption rate. The adsorption tests demonstrated that the Cr(vi) adsorption technique followed the Langmuir isotherm model and the pseudo-second-order kinetic model, suggesting that the process was an endothermic, monolayer chemisorption.^259^ Zhang *et al.* (2024) introduced a new composite membrane constructed from MXene and poly-melamine-formaldehyde (PMF). The amine groups in PMF and the OH group on MXene work together to improve pollutant adsorption, while glutaraldehyde prevents the membrane from swelling. In static adsorption tests, the prepared membranes of MXene/PMF-0.2 achieved removal efficiencies of 96.2% for Zn^2+^, 91.7% for Pb^2+^, 99.1% for phenol, and 96.4% for crystal violet, which is significantly superior to that of most MXene-based membranes reported in the literature. The regeneration examination demonstrated that the pollutant removal efficiency remained above 90% still after completing four cycles.^260^ Daulbayev *et al.* (2024) developed a three-dimensional scaffold composed of polyethyleneimine cryogel (PEI), multilayered Ti_3_C_2_T_*x*_ MXene, and silver (Ag) nanoparticles. Their research focused on evaluating its effectiveness in removing mercury (Hg) from solutions containing three distinct mercury salts: HgCl_2_, Hg(OAc)_2_, and Hg(NO_3_)_2_. The unmodified PEI cryogel achieved highest mercury (Hg) adsorption capacity of 340 mg Hg per g when using HgCl_2_ as the mercury source. However, incorporating MXene into the structure doubled the removal efficiency. When Hg(OAc)_2_ and Hg(NO_3_)_2_ were used as mercury sources, positively charged Hg^2+^ ions were generated, enhancing the material's mercury removal performance. The PEI/MXene composite achieved maximum adsorption capacities of 912 mg g^−1^ and approximately 840 mg g^−1^ for Hg(OAc)_2_ and Hg(NO_3_)_2_, respectively. Comparatively, the PEI/MXene@Ag system adsorbed 761 mg g^−1^ and 1280 mg g^−1^ of Hg from the same salts. The research proved that the PEI/MXene@Ag composite and the bare MXene and cryogels utilized multiple mechanisms towards the removal of mercury and presented the synergy of efficient adsorption of Hg(ii) and catalytic reduction.^261^

Dong *et al.* (2019) introduced new MXene (Ti_3_C_2_T_*x*_)/alginate composites, which were studied for their excellent adsorption capacity and rapid equilibrium times, making them effective adsorbents for the removal of Pb^2+^ and Cu^2+^ ions. The prepared composite, with its high adsorption capacity and quick equilibrium time, acquires maximum adsorption of Pb^2+^ and Cu^2+^ at 382.7 and 87.6 mg g^−1^, respectively, and achieves equilibrium within 15 minutes. Besides, it can be easily restored with acid handling and reused without significant reduction in performance.^262^

Ying *et al.* (2015) synthesized Ti_3_C_2_T_*x*_ nanosheets by etching bulk Ti_3_C_2_T_*x*_ powders using HF solutions at varying concentrations (50%, 25%, and 10%) followed by ultrasonic treatment. The resultant material demonstrated excellent efficiency in removing toxic Cr(vi) from waste water. Among the variants, Ti_3_C_2_T_*x*_ treated with a 10% HF solution showed the highest Cr(vi) removal capacity, getting 250 mg g^−1^, exceeding those treated with 25% HF (120 mg g^−1^) and 50% HF (170 mg g^−1^). Moreover, the residual Cr(vi) concentration in the treated water was reduced to less than 5 ppb, significantly below the World Health Organization's drinking water standard of 0.05 ppm for Cr(vi). This distinguished performance was associated with two key factors: (1) XRD analysis demonstrated that the Ti_3_C_2_T_*x*_-10% nanosheets had greater interlayer distances compared to Ti_3_C_2_T_*x*_-25% and Ti_3_C_2_T_*x*_-50%, suggesting that a decreased content of HF is more effective for facilitating intercalation and delamination; and (2) Ti_3_C_2_T_*x*_-10% exhibited the most increased specific surface area reaches 57 m^2^ g^−1^, particularly greater than Ti_3_C_2_T_*x*_-25% (20 m^2^ g^−1^) and Ti_3_C_2_T_*x*_-50% (9 m^2^ g^−1^), enhancing its ability to adsorb Cr(vi) more efficiently.^263^

##### 4.3.1.2. Organic pollutant removal

Functionalized MXene composites have also been used as photocatalysts to degrade and break down organic pollutants such as dyes, pesticides, and pharmaceuticals. The vast surface area and tunable bandgap impart a remarkable boost to their catalytic performance. The MXene group has been in the spotlight lately for its uses in different environmental processes, especially adsorption and decomposition. Organic pollutants used abundantly in printing, textiles, and paper manufacturing industries have been the main concern because of their designation as hazardous pollutants and mass discharge into water bodies.

Luo *et al.* (2025) prepared a novel MXene material, TiVCT_*x*_ by *in situ* etching. They explored its methylene blue (MB) dye cation removal ability by testing variables like adsorption time, pH values, effect of temperature, initial concentrations of both adsorbate and adsorbent, and interference of adsorbing methylene blue (MB) dye cation. The resultant differences reveal that TiVCT_*x*_ has superior (MB) adsorption ability with a maximum adsorption capacity of 1430 mg g^−1^. The performance is much better than other MXene materials. Besides, TiVCT_*x*_ achieves adsorption equilibrium of (MB) in 30 s, reflecting its superior adsorption rate. The (MB) adsorption test showed the technique adopted a Freundlich isotherm model and the pseudo-second-order kinetic model and it indicates the MB adsorption was an exothermic multilayer chemisorption. Both XPS and zeta potential measurements illustrate that, under the alkaline condition, the predominant mechanisms of MB adsorption are hydrogen bonding and electrostatic interactions.^259^

Li *et al.* (2025) synthesized Mn_3_O_4_/MXene composites using a combination of hydrothermal and calcination processes. The methodology efficiently tuned the Mn_3_O_4_ activation mechanism for PMS and facilitated a significant enhancement of the degradation efficiency of bisphenol A (BPA). The optimized Mn_3_O_4_/MXene composites show a first-order rate constant of 0.0218 g (m^−2^ min^−1^), compared to a factor of 8.4 and a factor of 2.0 higher than MXene and Mn_3_O_4_, respectively. The catalyst also exhibits exceptional mineralization performance with maximum total organic carbon removal efficiency as high as 88.2%.^264^

Mansoor *et al.* (2024) proposed a high-performance flowing electrode capacitive deionization (FE-CDI) system to remove ammonia. The system exhibited remarkable performance and used flowing electrodes consisting of Ti_3_C_2_T_*x*_ MXene at a concentration of 1 mg mL^−1^. Despite having low material loading of the Ti_3_C_2_T_*x*_ flowing electrodes, the system showed significantly improved performance with ion removal efficiency to the tune of 60% in a period of 115 minutes and an adsorption capacity of 460 mg g^−1^. The system obtained a charge efficiency of between 58% and 70% and had a power consumption of merely 0.45 kWh per kg removed ions. Moreover, a regeneration efficiency of 92% was a testament to the stability of the electrodes and their ability to work in long-term and broader applications.^253^

Miri-Jahromi *et al.* (2022) examined Ti_2_C MXene and superior adsorption capacities with semi-synthetic antibiotics like amoxicillin, ampicillin, and cloxacillin. Data analysis indicated that Ti_2_C-MXenes were found to adsorb antibiotic compounds with a 100% adsorption of cloxacillin, 88% of amoxicillin, and 44% of ampicillin. The research indicates that MXene functionalization with hydroxyl and amine groups improves its capacity to adsorb antibiotics and further validates that Ti_2_C could be reused as a good adsorbent.^265^ Kim *et al.* (2021) explored the ability of multilayered 2D MXene of Ti_3_C_2_T_*x*_ to adsorb pharmaceutical substances and found amitriptyline (AMT) and verapamil (VRP) to exhibit better adsorption efficiency. The capacity of AMT reached its maximum value of 58.7 mg g^−1^ at pH 7, resulting from the electrostatic interaction between negatively charged MXene and AMT having a positive charge. In order to enhance the performance of adsorption, MXene was sonicated at 0, 28, and 580 kHz to adsorb AMT and the maximum capacity was found to be at 28 kHz (214 mg g^−1^), followed by 580 kHz (172 mg g^−1^) and 0 kHz (138 mg g^−1^). Sonication generates cavitation bubbles to better disperse MXene and promote the formation of oxygenated functional groups on its surface.^266^

Yao *et al.* (2021) proposed a feasible route to synthesize a porous MXene/single-walled carbon nanotube film as a freestanding electrode whose vast surface area, electrostatic interactions and hydrogen bonding facilitated efficient electrosorption of MB from wastewater. The as-prepared film has a remarkable adsorption amount of methylene blue (MB) up to 1068.8 mg g^−1^ under an applied voltage of −1.2 V, much higher than the 55.8 mg g^−1^ under open-circuit condition. The electrode shows good reusability with a maximum adsorbing capacity of up to 28 403.7 mg g^−1^, shows high selectivity towards MB and methyl orange (MO) by pH adjustment and maintains around 95.2% of its capacity in five-cycle reuse with no secondary pollution.^267^

Cai *et al.* (2020) synthesised a novel MXene composite material (phytic acid (PA)–MXene) through a simple hydrothermal process and discovered that the PA–MXene composite had better adsorption capacity towards Rhodamine B (RhB) and retained around 85% of its adsorption capacity even up to the 12th cycle, opening up its possibility to treat wastewater.^268^

Wojciechowski *et al.* (2019) investigated the synthesis, characterization, band gap evaluation, and photodegradation efficiency of salicylic acid (SA) through the employment of Ti_2_C MXene with six composites prepared by modifying MXene with TiO_2_, Ag_2_O, Ag, PdO, Pd, and Au. Experimentally identified band gaps by employing Tauc's formula varied from 0.90 to 1.31 eV. All MXene-derived specimens exhibited very good activities in the photodegradation of (SA), with a degradation rate of 86.1–97.1% in 3 hours when the initial SA concentration was 100 µM and 0.875 cm^3^ min^−1^.^269^

Iqbal *et al.* (2019) explored the photo-degradation of acetophenone and Congo Red in aqueous solutions upon visible light illumination. For harnessing solar energy in photocatalysis, they developed a nanohybrid system as a blend system of two-dimensional (2D) MXene sheets and bismuth ferrite nanoparticles (BFO)/Ti_3_C_2_ (MXene). The hybrid composite was found to exhibit total photocatalytic degradation with 100% removal of the organic dye (Congo Red) and the colorless pollutant (acetophenone) in 42 and 150 minutes, respectively, this process predominantly due to large surface area which was 147 m^2^ g^−1^.^270^

Zhu *et al.* (2019) produced a two-dimensional MXene modified with Fe_3_O_4_ by adopting an *in situ* growth method and investigated its properties and methylene blue adsorption capacities at varied temperatures. The reduction process resulted in a decolorization efficiency of 91.93% at a temperature of 55 °C, with adsorption conforming to Freundlich isotherm at higher temperatures (40–55 °C) and Langmuir isotherm at 25 °C. The thermodynamic study indicated the MB removal as an exothermic chemisorption process and the increase of removal efficiency of the developed composite at a higher temperature.^271^

Wei *et al.* (2018) prepared Ti_3_C_2_T_*x*_ and Alk-Ti_3_C_2_T_*x*_ and examined their ability to adsorb cationic dye (MB). Treatment with an alkali solution to produce Alk-Ti_3_C_2_T_*x*_ facilitated the expansion of MXene's interlayer spacing and transformation of surface functional groups, which improved the material's adsorption capacity and increased the rate of MB elimination. During the procedure, LiOH increased the interlayer spacing of Ti_3_C_2_T_*x*_ MXene by as much as 29%, while also modifying the surface functional groups by replacing –F with –OH. The adsorption behavior of Alk-Ti_3_C_2_T_*x*_ obeys the Langmuir model, indicating that Alk-Ti_3_C_2_T_*x*_ has a uniform adsorption surface and can form only a single layer of adsorbate. The study indicated that NaOH-treated Ti_3_C_2_T_*x*_ exhibits the most increased adsorption capacity and rapid MB eliminated rate (189 mg g^−1^), mainly due to the combined effects of surface and intercalation adsorption techniques.^272^

Shahzad *et al.* (2018) employed a TiO_2_/Ti_3_C_2_T_*x*_ MXene composite for the photocatalytic breakdown of the carbamazepine (CBZ). The investigation showed that the composite reached a degradation efficiency of 98.67% within 4 hours. The photocatalytic reaction kinetics were found to adhere to the Langmuir–Hinshelwood model, with a Kapp value of 0.0304 min^−1^ under ultraviolet light, which was higher than that observed under natural solar light. Additionally, the degradation efficiency was significantly influenced by acidic conditions, particularly in the pH range of 3.0–5.0. In the photocatalytic degradation process, hydroxyl radicals (OH) and oxygen (O_2_) interacted with the CBZ molecule, and based on these interactions, complex degradation routes were suggested.^273^Table 7 summarizes the common characteristics of pollutants.

**Table 7:** Common provided specifications of the pollutants

Pollutant	Material	Max *Q* (mg g^−1^)	Kinetics (*t*_90_)	Regeneration cycles	Notes/method	Reference
Cr(vi)	Fe_3_O_4_@MCLS (MXene–chitosan–lignosulfonate)	42.5	Equilibrium ≈60 min	4 cycles; *Q* ∼33.2	Batch removal 90% efficiency	258
Cr(vi)	TiVCT_*x*_ (novel MXene)	553	Equilibrium ∼10 min	—	Langmuir monolayer chemisorption	259
Hg(ii) (HgCl_2_)	PEI/MXene composite	875	Equilibrium ∼48 h	—	Multiple mechanisms; enhanced by positive Hg^2+^	261
Hg(ii) Hg(OAc)_2_	PEI/MXene composite	761	Equilibrium ∼48 h	—	Multiple mechanisms	261
Hg(ii) Hg(NO_3_)_2_	PEI/MXene@Ag	1280	Equilibrium ∼48 h	—	Synergy of adsorption + catalytic reduction	261
Pb(ii)	Ti_3_C_2_T_*x*_/alginate composite	382.7	Equilibrium ∼15 min	No significant loss	Rapid adsorption	262
Cu(ii)	Ti_3_C_2_T_*x*_/alginate composite	87.6	Equilibrium ∼15 min	No significant loss	Rapid adsorption	262
Cr(vi)	Ti_3_C_2_T_*x*_ (10% HF etched)	250	Equilibrium ∼150 min	—	Residual <5 ppb; increased surface area/interlayer spacing	263
Zn(ii)	MXene/PMF-0.2 membrane	Removal 96.2%	—	4 cycles; >90%	96.2% removal; >90% after 4 cycles	260
Pb(ii)	MXene/PMF-0.2 membrane	Removal 91.7%	—	4 cycles; >90%	91.7% removal; >90% after 4 cycles	260
MB	TiVCT_*x*_ (novel MXene)	1406	Equilibrium ∼30 s	—	Freundlich; exothermic multilayer chemisorption	259
MB	Porous MXene/SWCNT freestanding film	1068.8	—	∼95.2% after 5 cycles	At −1.2 V; high selectivity *via* pH tuning	267
Rhodamine B (RhB)	Phytic acid–MXene (PA-MXene)	22.1	Equilibrium ∼12 h	∼85% after 12 cycles	Hydrothermal composite	268
MB	Phytic acid–MXene (PA–MXene)	41.4	Equilibrium ∼12 h	∼85% after 12 cycles	Hydrothermal composite	268
MB	Alkali-treated Ti_3_C_2_T_*x*_ (Alk-Ti_3_C_2_T_*x*_)	189	Rapid	—	Interlayer expansion; Langmuir monolayer	272
Amitriptyline (AMT)	Ti_3_C_2_T_*x*_ (sonicated 28 kHz)	214	—	90% after 5 cycles	pH 7; sonication enhances dispersion/functional groups	266
Antibiotics (amoxicillin, ampicillin, cloxacillin)	Ti_2_C MXene (functionalized)	Removal 66%	—	Reusable; % removal AMX 88, AMP 44, CLOX 100	Hydroxyl/amine groups improve adsorption	265
Phenol	MXene/PMF-0.2 membrane	Removal 99.1%	—	4 cycles; >90%	99.1% removal; >90% after 4 cycles	260
Crystal violet (CV)	MXene/PMF-0.2 membrane	Removal 96.4%	—	4 cycles; >90%	96.4% removal; >90% after 4 cycles	260
Ammonium (NH_4_^+^)	Ti_2_C_2_T_*x*_ FE-CDI	460	—	—	Low-energy consumption of 0.45 kWh kg^−1^	253

##### 4.3.1.3. Removal of radioactive nuclides

MXene and its derivatives are the best adsorbents for the removal of radioactive isotopes such as uranium (^238^U), thorium (^232^Th), cesium (^137^Cs), and strontium (^90^Sr), and possess advantages in terms of both technological and economic considerations. The isotopes possess long half-lives and fast mobility in aqueous media and pose a serious threat to water ecosystems and human health. Consequently, providing safe and efficient wastewater treatment contaminated by radioactive isotopes is a serious issue in environmental fields. MXenes possess a range of properties consisting of high specific surface area, better hydrophilicity, many active surface sites on the surface, strong mechanical strength, and tunable interlayer distance. Besides, they exhibit good resistance to radiation damage, good compatibility with chemicals, and high thermal stability and permit the preparation of MXene derivatives with tailored structures and varied compositions.^274–277^

Zhang *et al.* (2025) used an *in situ* assembling technique to build an integrating zeolitic imidazolate framework (ZIF-67) into the two-dimensional (2D) layers of transition metal carbides/nitrides (MXenes) Ti_3_C_2_. The sensitized composite was used to adsorptively remove Cs^+^ from wastewater and had a removal efficiency of 96.2% at a Cs^+^ loading of 5 mg L^−1^ in 6 hours and a maximum uptake capacity of 50.0 mg g^−1^. The adsorbent also showed consistent reusability up to four cycles.^278^

Wang *et al.* (2024) fabricated hierarchical potassium titanate nanostructures (K-HTNs) from MXene by a simple molten salt process using the precursor of Ti_3_C_2_T_*x*_ MXene and were targeted towards efficient recovery and removal of Sr(ii) from wastewater and seawater. The study showed the pH effect on the adsorption of Sr(ii) onto K-HTN and reached a maximum adsorption capacity of 204 mg g^−1^ with a removal efficiency of 93.3% of Sr(ii) in real seawater under ambient temperature. Impressively, adsorption efficiency decreased by merely 2.2% when five adsorption–desorption cycles were conducted, which exemplifies the superior reusability of K-HTNs towards Sr(ii) recovery. Removal was explained by the exchange of Sr^2+^ with K^+^ or H^+^ ions in the interlayers of K-HTN. The adsorbed Sr(ii) also engaged in strong interactions with the available Ti–O– groups on the titanate structure.^279^

Wei *et al.* (2024) explored the viability of a thin film nanofiltration membrane of a ZIF-8@Ti_3_C_2_T_*x*_ composite material nanocomposite, which was successfully developed by interfacial polymerization of a composite material of ZIF-8@Ti_3_C_2_T_*x*_ in a polymer using a microemulsion with the ZIF-8@Ti_3_C_2_T_*x*_ as a doping agent. The electronegativity of the developed composite enhanced the negative charge on the surface of the composite membrane and resulted in a remarkable modification of its ability to reject ReO_4_^−^ without hampering a low retention rate of Na^+^. The performance as seen using a microemulsion doping concentration of 0.5 g L^−1^ was optimal (*J*_p_ = 41.92 L m^−2^ h^−1^ and *R*(ReO_4_^−^) = 65.81%). In fact under a higher salt condition (20 g L^−1^), the M–O-1 membrane was found to retain approximately 55% of impurities at a continuous operation of 5 hours.^280^

Liu *et al.* (2023) presented a bio-adsorbent (DACNF–MXenes–NH_2_–PT) fabricated using a simple technique whereby dialdehyde cellulose nanofibers (DACNF) were cross-linked with amino-functionalized MXenes (MXenes–NH_2_) and persimmon tannin (PT). The resultant composite had high adsorption capacities towards U(vi) (105.7 mg g^−1^) and Th(iv) (95.1 mg g^−1^). In addition, equilibria analysis showed the adsorption behavior to conform to the Langmuir type isotherm and pseudo-second-order kinetic models and imply the process of extraction to be dominated by electron exchange interactions between the bio-adsorbent and target ions. Most interestingly, competitive adsorption experiments showed that the composite had a sharp selectivity towards U(vi) and Th(iv). The removal efficiency of the material had a simple decrease following three adsorption and desorption cycles.^281^

Zhao *et al.* (2022) surface-functionalized Ti_3_C_2_T_*x*_ MXene with polyhedral oligomeric silsesquioxanes (POSS-OHs) and hydroxypropyl celluloses (HPCs). The functionalized Ti_3_C_2_T_*x*_ MXene had a remarkably increased adsorption capacity of uranium ions of around 307.67 mg g^−1^, which was a 35.08% increase compared to the non-modified performance. In addition to that, the analysis results showed that uranium ion adsorption consisted of a twofold mechanism involving strong coordination with hydroxyl groups and electrostatic force.^282^

Zhang *et al.* (2022) also prepared amidoxime-functionalized TC–AO by modifying the surface of Ti_3_C_2_T_*x*_ MXene using amidoxime groups by diazonium salt grafting. The work explored numerous variables affecting radionuclide elimination efficiency, including the course of the reaction time, temperature levels, pH levels, electrolyte strength, and presence of various anions and cations. The data reaffirmed TC–AO to exhibit fast kinetic rates of reaction by achieving equilibrium in around 20 minutes as well as good elimination capacities of 279.57 mg g^−1^ and 73.99 mg g^−1^ towards U and Eu, respectively. The material also showed good selectivity and good stability.^283^

Jun *et al.* (2020) explored the use of MXene (Ti_3_C_2_T_*x*_) as an adsorbent to collect Ba^2+^ and Sr^2+^ ions from a simulated wastewater model. The work showed MXene to have very high adsorption capacities of around 180 mg g^−1^ and 225 mg g^−1^ for Ba^2+^ and Sr^2+^ ions, respectively. These were obtained using a mixture of MXene with adsorbates of concentration 1 g L^−1^ and adsorbate of concentration 2 g L^−1^. MXene was found to have a greater affinity towards Sr^2+^ than Ba^2+^ due to the disparity in their electronegativity. MXene presented very fast adsorption characteristics by reaching an adsorption state in under an hour and reusability experiments verifying MXene to work with remarkable efficiency at least up to four successive recycles.^284^

Zhang *et al.* (2020) have outlined a process to modify the MXene surface *via* carboxylation by using diazonium salts. Carboxyl functionalization resulted in significantly improving MXene's ability to efficiently collect radionuclides. The carboxy-modified Ti_3_C_2_T_*x*_ MXene (TCCH) had exceptional efficiency in the removal of U(vi) and Eu(iii), evidenced by incredibly fast adsorption time of only 3 minutes, astounding maximum adsorption capacities of as much as 344.8 mg g^−1^ of uranium and 97.1 mg g^−1^ of europium, and more than 90% removal efficiency of radionuclides from the simulated model. The results obtained show that adsorption of U(vi) on TCCH takes place by an inner-sphere coordination mode of action and adsorption of Eu(iii) by a synergism of inner-sphere complexation and electrostatic interactions.^285^

Jun *et al.* (2020) also explored MXene (Ti_3_C_2_T_*x*_) as a possible means to remove radioactive Cs from simulated nuclear wastewater under different influencing factors such as MXene dosage, initial Cs concentration, pH levels, solution temperature, and contact time. MXene recorded fast adsorption kinetics and reached the equilibrium state in a contact time of merely 1 hour. MXene was found to possess a remarkable adsorption capacity of up to 148 mg g^−1^ at the values of 5 mg L^−1^ of the adsorbent and 2 mg L^−1^ of the adsorbate under neutral pH (pH 7).^286^

##### 4.3.1.4. Pesticides removal and sensing

Pesticide compounds are commonly used to increase crop yield but are prone to causing environmental contamination and pose a serious threat to human health when used excessively. The toxic effect of pesticides is primarily attributed to their tendency to permanently bind to acetylcholinesterase (AChE) and inhibit its action, leading to a severe neurological injury, organ failure, and even mortality. Owing to their hydrophilicity, high electronic conductivity, huge surface area, and good electrochemical properties, MXenes are attracting considerable interest as supercapacitors, sensors, fuel cells, and lithium-ion batteries. Moreover, the addition of noble metal nanoparticles such as gold or silver to MXene increases their conductivity and electrochemical activity and widens their scope of application in electrochemical biosensing purposes.^287–290^

Yola *et al.* (2024) constructed a manganese dioxide nanoprobe modified glassy carbon electrode using cyclic voltammetric electro-polymerization of a pyrrole monomer solution. The electrode was used to directly sense the contaminated fenitrothion pesticide. The results showed that the constructed nanocomposite was found to possess high conductivity, a high surface area, and an efficient sensing interface to sense fenitrothion. The sensor was found to have a linear range of 1.0 × 10^−9^ to 2.0 × 10^−8^ mol L^−1^ and a minute limit of detection equal to 3.0 × 10^−10^ mol L^−1^. The developed sensor was evaluated on white flours and returned values close to a value of 100%.^289^

Cao *et al.* (2024) proposed a thermal-assisted CoFe_2_O_4_/MXene system to activate peroxymonosulfate (PMS), with a remarkable 98.90% removal of atrazine (ATZ) under a condition of 1 min with a dose of 0.1 g L^−1^ of CoFe_2_O_4_/MXene and 60 °C. This system had a rate of reaction of 2.33 min^−1^ and was six times faster than the CoFe_2_O_4_/MXene-PMS system. The synergistic mechanism study indicates that heat facilitates mass transfer, ruptures the O–O bond to activate PMS more strongly and creates multiple oxygen vacancies (OV) in CoFe_2_O_4_/MXene. Moreover, the synergistic system also has a different pathway to degrade ATZ and maintained a close to 100% efficiency of removing ATZ during five consecutive cycle.^291^

Sharma *et al.* (2022) constructed a novel MXene–reduced graphene oxide (rGO) nanocomposite. The biosensor using an MXene–rGO/endosulfan Ed-Ab electrode was optimized to achieve electrochemical efficiency with a limit of detection of 0.497 ppt of Ed and exhibited low cross-reactivity towards other organochlorine insecticides. The sensor was stable for 21 days and had uniform performance to four successive services. Further on, it was successfully used to detect Ed in many examples such as root, leaf and water extracts and was found to be apt to monitor pesticide contamination.^292^

Guo *et al.* (2022) presented a direct hydrothermal route to synthesize Ti_3_C_2_–MoS_2_ composites and explored how they are as active adsorbers and removers of the organic pesticide paraquat (PQ). Adsorption equilibrated in 30 minutes and increased significantly from 8.7 mg g^−1^ to 105.53 mg g^−1^ compared to raw Ti_3_C_2_. In addition to that, PQ was adsorbed by spontaneous and exothermic means by Ti_3_C_2_–MoS_2_, and performance was modulated by multiple parameters such as temperature, pH, and ion strength. The increased adsorption effect of Ti_3_C_2_–MoS_2_ is explained by the increased surface area by the addition of flower-like MoS_2_. Moreover, the semiconducting ability of Ti_3_C_2_–MoS_2_ might promote the catalytic degradation of PQ.^293^

Khosrowshahi *et al.* (2022) has developed a novel technique in dispersive solid-phase extraction (DSPE) utilizing MXene (Ti_2_AlC) as a potent medium to isolate and quantify twelve different pesticides from fresh fruit juice samples. HF etching was used to prepare MXene to produce a characteristic accordion-shape nanostructure with a high surface area and plentiful active functional groups. The developed technique exhibited good performance with low detection values (0.08–1.0 µg L^−1^), good recoveries (69% to 75%), and a good reproducibility with relative standard deviations (RSD) less than or equal to 5.5%. The technique was used to identify pesticide residues at permissible levels. This novel technique highlights the strength of MXene as a tool in the analysis of pesticides in food safety and its applicability in sensing and environmental cleanup.^294^

Sinha *et al.* (2021) synthesized single-layered Ti_3_C_2_T_*x*_ MXene *via* the MILD (minimally intensive layer delamination) process to sense methiocarb and diethofencarb. They used 7.5 M lithium fluoride (LiF) and 9 M hydrochloric acid (HCl) to produce *in situ* HF as an etching agent to eliminate aluminum from the Ti_3_C_2_T_*x*_ MAX phase. Exfoliated sheets of Ti_3_C_2_T_*x*_ were found to comprise single or multiple layers with the support of surface groups like hydroxyl or oxygen linked. The as-prepared Ti_3_C_2_T_*x*_ sheets were used as electrodes and exhibited real-life sensing of methiocarb and diethofencarb through voltammetry. The sensing range of methiocarb and diethofencarb was found to be 0.19 µg m L^−1^ and 0.46 µg m L^−1^.^287^

Song *et al.* (2019) presented a novel electrochemical sensing platform with high sensitivity towards detecting organophosphorus pesticides (methamidophos) using composites of MnO_2_/Mn_3_O_4_-derived from MOFs and Au NPs decorated on MXene-derived Ti_3_C_2_ MXene. The sensing platform based on the developed composite detects methamidophos in a broad range of concentrations (10^−12^ to 10^−8^ M) with a high linearity (*R* = 0.995). The biosensor also has a very low limit of detection (1.34 × 10^−13^ M), much less than the maximum residue levels (MRLs) of methamidophos (0.01 mg kg^−1^) established by the European Union.^295^

##### 4.3.1.5. Sea water desalination and filtration

MXene membranes were found to exhibit high rates of salt rejection and water permeability. The laminated structure facilitates ion selectivity and has been used to achieve efficient desalination and water purification. The use of solar-driven interfacial evaporation as a technique to increase energy efficiency and foster sustainable ways of seawater desalination and water purification is a promising area of research. High evaporation efficiency requires material design by carefully controlling material structures, primarily porosity and surface chemistry. Innovative and sustainable materials are essential to utilize in solar water desalination as clean water becomes increasingly in demand.

Xie *et al.* (2024) developed a stable MXene@oleylamine/polyethylene terephthalate (MXene@OAm/PET) nonwoven with strong chemical cross-linking between oleylamine-modified MXene and PEG-treated PET, offering remarkable hydrophobicity and resistance to oxidation. The results demonstrated efficient solar absorption and reached an evaporation rate of 1.26 kg m^−2^ h^−1^ in 3.5 wt% NaCl solution. Furthermore, the prepared composite maintained a stable evaporation rate of approximately 1.22 kg m^−2^ h^−1^ after 40 days in a 3.5 wt% NaCl solution, highlighting its strong potential for prolonged seawater desalination and industrial use, with an evaporation rate of 1.27 kg m^−2^ h^−1^ even in industrial wastewater.^296^

Si *et al.* (2025) investigated porous MXene membranes to desalinate water efficiently using molecular dynamics calculations to analyze Ti_2_CF_2_, Ti_3_C_2_F_2_, and Ti_4_C_3_F_2_ membranes. The results obtained showed water flux to decrease as thickness increases (Ti_2_CF_2_ > Ti_3_C_2_F_2_ > Ti_4_C_3_F_2_), with Ti_2_CF_2_ rejecting ∼80% of ions and both Ti_3_C_2_F_2_ and Ti_4_C_3_F_2_ having a 100% ion rejection. The thicker transition metal layers are seen to reduce water flux from a higher water density in the pores, whereas charge distribution and the free energy analysis confirm the transition metal layer to block Na^+^ and trap Cl^−^.^297^

Zaed *et al.* (2024) have shown a new hexagonal-shaped evaporator consisting of a hydrophilic Ti_3_C_2_T_*x*_ MXene-coated carbonized (CHC) nanocomposite (Ti_3_C_2_T_*x*_ MXene@CHC). The evaporator has a remarkable water evaporation rate of 1.6 kg m^−2^ h^−1^ at 1 sun illumination with a high efficiency of 90%. The hydrophilic properties of the nanocomposite increase water molecule escape by augmenting the water layer thickness and the hydrophobic areas extended contact lines further augment evaporation.^298^

Farabi *et al.* (2024) used a MXene and RuO_2_ nanocomposite coated on a biodegradable luffa sponge. The MXene restacking is avoided by ruthenium oxide (RuO_2_), which reduces van der Waals interactions, while the large pores and robust structure of the biodegradable luffa sponge make it a good evaporator. The novel MXene–RuO_2_@LS absorber showed a rate of evaporation around 1.5 kg m^−2^ h^−1^ when exposed to solar illumination of one hour. The result also had an energy efficiency of 90.85%, with a very low heat loss of only 9.15%.^299^

Chen *et al.* (2024) constructed an MXene-based charge-gradient hydrogel evaporator using the Donnan effect to repel salts and internal osmotic pressure to promote water transport. The MCGH evaporator had an evaporation rate of 3.1 kg m^−2^ h^−1^ when it was under solar irradiation in a 3.5 wt% NaCl aqueous solution and had good performance in concentrated brine with a rate of 2.1 kg m^−2^ h^−1^ in 20 wt% NaCl.^300^

Zaed *et al.* (2024) developed a solar evaporator constructed by depositing Ti_3_C_2_T_*x*_ MXene on carbon-enriched cellulose fibers (CCF), to produce a composite of Ti_3_C_2_T_*x*_ MXene@CCF that was used in sustainable solar-powered water desalination. The developed composite had a superior evaporation rate of 3.8 kg m^−2^ h^−1^ when illuminated by a single sun. The hydrophilic MXene film on the porous CCF promotes efficient evaporation of water at high evaporation rates and keeps water clean.^301^

Zaed and others (2024) also pointed out the suitability of a Ti_3_C_2_T_*x*_-coated polypropylene fiber (PPF) composite as a desalination material, considering how it could serve as a very efficient solar desalination system. The composite had a remarkable efficiency of 93.48% because it has a remarkable solar energy absorption capability and produces solar vapor at a rate of 4.63 kg m^−2^ h^−1^. The process is a purification process using solar power and can clean water to the tune of 23–25 Lper m^2^ per day and operates for up to 5 hours in normal sunlight. The material was physically robust, was also durable against acidic and alkali environments, was good at resisting the buildup of salt and continued to work consistently for extended hours without decreased performance.^302^

Ding *et al.* (2020) report MXene non-swelling membranes obtained by intercalation of Al^3+^ ions. The swelling prevention results from the strong interactions between Al^3+^ ions and O– functional groups on the MXene surface. The engineered membranes show high resistance to swelling in water solutions up to 400 hours and high NaCl rejection rates of ∼89.5–99.6% and fast water fluxes of ∼1.1–8.5 L m^−2^ h^−1^.^303^

#### 4.3.2. Air purification

MXenes are 2D materials with excellent mobility, a large specific surface area, and a tunable band gap, making them highly versatile for various applications. They offer numerous active sites that enhance gas adsorption capabilities such as SO_2_, CO_2_, and NH_3_ due to strong affinity for gas molecules adsorption, especially terminations play a critical role in enhancing gas adsorption capacities.

Liu *et al.* (2025) fabricated nanocomposite inks composed of titanium dioxide (TiO_2_) and MXene (Ti_3_C_2_), with TiO_2_ nanoparticles positioned between the Ti_3_C_2_ layers. Gas adsorption experiments were performed by depositing these TiO_2_/Ti_3_C_2_ nanocomposite inks onto gold-coated silicon wafers using ultrasonic spray printing. Gas adsorption investigations revealed that the TiO_2_/Ti_3_C_2_ MXene nanocomposites exhibit remarkable selectivity for butane over other alkane gases. The butane adsorption capacity reached 18.81 cm^3^ g^−1^, exceeding the maximum adsorption capacities for methane, ethane, and propane by 10.19 cm^3^ g^−1^, 16.18 cm^3^ g^−1^, and 16.28 cm^3^ g^−1^, respectively.^304^

Wang *et al.* (2024) investigated the gas sensing properties of M2CF2 (M = Sc, Ti, V) MXenes towards AsH3 *via* density functional theory (DFT). The research found weak physisorption on the pristine MXenes but increased chemisorption with adsorption energies as high as −2.166 eV and recovery times as low as 3.96 s.^305^

Zhang *et al.* (2024) synthesized α-Fe_2_O_3_/Ti_3_C_2_T_*x*_ MXene composites with a two-dimensionally layered structure using a hybrid of hydrothermal and annealing methods. The α-Fe_2_O_3_ blend with the inclusion of Ti_3_C_2_T_*x*_ MXene enabled the composite sensor to work optimally at room and bending states and boosted its gas sensing characteristics by a considerable margin. The α-Fe_2_O_3_/Ti_3_C_2_T_*x*_ composite showed a response of 20.27 to 100 ppm of hydrogen sulfide at a room temperature of 25 °C compared to pure α-Fe_2_O_3_ under the same condition with a response of merely 5.17.^306^

Shin *et al.* (2024) developed an H_2_S gas sensor using a MXene/MoS_2_ heterostructure fabricated by combining the Langmuir–Blodgett (LB) technique and chemical vapor deposition (CVD). The developed sensors showed a remarkable increase in gas sensitivity. While the response of pure MXene towards H_2_S was 0.1, the MXene/MoS_2_ heterostructure showed a much higher response of 0.5, representing a five-fold increase in sensitivity. The increase arises from the formation of a heterojunction, which facilitates electron mobility and reduces the depletion layer to increase gas sensing. The developed composite also showed remarkable selectivity sensing towards H_2_S, as against other gases like H_2_, NO_2_, NH_3_, NO, and VOCs.^307^

Dong *et al.* (2023) investigated VTi-deficient VTi–Ti_3_C_2_O_2_ and TM-doped (TM = Pt, Au and Ti) Ti_3_C_2_O_2_ as gas-sensing materials and by means of density functional theory DFT analyzed the adsorption structures, energies, electron densities, and desorption times of SO_2_, SOF_2_, and SO_2_F_2_ on the materials. The results obtained show that Pt–Ti_3_C_2_O_2_ and Au–Ti_3_C_2_O_2_ facilitate facile desorption of gases through physical adsorption while VTi–Ti_3_C_2_O_2_ has a strong tendency to undergo chemical adsorption and hence gas desorption is difficult. TM doping also increases the gas adsorbing capability of pristine Ti_3_C_2_O_2_ towards SF_6_ decomposition products.^308^

Li *et al.* (2023) showed a recent theoretical work investigated the SO_2_–Hf_2_CO_2_ monolayer interaction revealing weak adsorption on the clean surface. The drawback of this was overcome by analyzing the preadsorption of different gas molecules, especially H_2_O. The presence of preadsorbed H_2_O was shown to strongly enhance SO_2_ adsorption by shifting the system to a chemisorption state. Not only did this increase the strength of the adsorption but also decreased recovery times, and the systems were shown to hold promise as a means of reusable SO_2_ gas sensing.^309^

Li *et al.* (2023) explored the gas sensing properties of Ti_3_C_2_O_2_ and Pt–Ti_3_C_2_O_2_ towards NH_3_, H_2_S, and CO_2_ through DFT calculations. Although Ti_3_C_2_O_2_ shows good conductivity, it has a poor gas adsorption ability through the absence of surface defects. The addition of Pt strongly enhances the sensing ability by creating active adsorption sites, enhancing charge transfer, and reducing adsorption distances with the binding energy (−1.796 eV) and transferred charge (0.373*e*). The Pt–Ti_3_C_2_O_2_ composite shows high interactions with NH_3_ with a considerable transfer of electrons. The adsorption energy for the system of NH_3_ is found to be −2.593 eV with the donation of 0.327 electrons by NH_3_ to the Pt–Ti_3_C_2_O_2_ surface.^310^

Javaid *et al.* (2022) reported noble gas adsorption Xe and Kr on Ti- and V-based MXenes was explored using van der Waals-corrected DFT simulations. Adsorption energies for Xe and Kr on V_2_C reached up to (−0.32, and −0.19 eV), with distances of (3.12, and 3.17 Å), exceeding Ti_2_C (−0.18, and −0.12 eV), (3.47, and 3.66 Å). V_2_C demonstrated enhanced Xe selectivity over Kr and stronger interfacial interactions.^311^

Zeng *et al.* (2022) presented adsorption process of Ti_3_C_2_T_*x*_ (T = O, F, OH) towards the reduction of NO, NO_2_, N_2_O, and NF_3_. The results showed all the four gases could adsorb spontaneously on the surface of Ti_3_C_2_T_*x*_ and that Ti_3_C_2_(OH)_2_ had the highest adsorption energies and greatest charge transfers to NO, NO_2_, N_2_O, and NF_3_ compared to Ti_3_C_2_F_2_ and Ti_3_C_2_O_2_. The adsorptions increased the electrical conductivity of all the materials towards NO and NO_2_ but improved conductivity was found in case of Ti_3_C_2_(OH)_2_ towards NF_3_ and N_2_O. Strong chemisorption was found on Ti_3_C_2_(OH)_2_ towards NF_3_ and N_2_O showing strong interactions and high charge transfer.^312^

Banu *et al.* (2022) presented a comprehensive computational investigation of the adsorption of NH_3_ on MXenes (M_2_C; M = Cr, Fe) and their oxygen-functionalized analogs (M_2_CO_2_) showing interesting gas-surface interactions. Pristine M_2_C (*E*_ad_ = −1.40/−1.33 eV) surfaces exhibit strong chemisorption controlling adsorption of NH_3_, but oxygen termination decreases the reactivity (*E*_ad_ = −0.29/−0.22 eV), transforming the interaction to weak physisorption. Significantly, Cr_2_CO_2_ shows remarkable stability and selectivity towards sensing of NH_3_ and this has been attributed to weak adsorption and efficient electron transfer.^313^

The impact of atomic defects in Mo_2_TiC_2_T_*x*_ MXenes on their electronic and electrochemical properties and CO_2_ adsorption properties has been investigated by Khaledialidusti *et al.* (2020) using density functional theory (DFT). The outcomes presented showed that defects greatly increase CO_2_ adsorption by providing sites for exothermic and spontaneous interactions and adsorption energies as low as −0.35 eV at distances ranging from 2.87 to 2.90 Å, while pristine MXene surfaces reveal weak physisorption.^314^

## 5. Advancements in materials for enhancing MXene stability: applications and research trends

A wide variety of polymers, carbon-based materials, metal oxides, hybrid materials, functional groups, and 3D architectures represent the development of various materials to improve MXene stability. These have been shown to create revolutionary impacts on a wide array of energy-related applications: batteries, supercapacitors, hydrogen production, and catalysis. Protection with appropriate material for improving MXene properties has been sensitively designed to overcome several critical challenges regarding oxidation, layer restacking, and mechanical instability, therefore optimizing its electrochemical performances.

The major role that PSt and similar polymers serve, together with PIB, is to prevent, in the end, oxidation and maintain conductivity over time. Their somewhat insulating nature creates layer protection, thus prolonging most of the operational life stages of MXene-based devices. Conductive kinds of polymers, specifically PANI and Ppy, go a step further by combining electrical conductivity with improved mechanical stability, which turns materials suitable for supercapacitors and flexible electronic devices.

It mainly includes carbon-based materials: graphene oxide, reduced graphene oxide, and carbon nanotubes as structural supports or spacers that prevent the restacking of MXene layers. This property not only improves ion accessibility but also improves energy storage efficiency, rendering them indispensable in advanced systems for batteries and supercapacitors. For example, CNTs are highly valued, especially for their role in facilitating ion transport and the structural integrity of electrodes.

Most impressive values for chemical stability and electrochemical performance are achieved in hybrid-like MXenes, such as titanium dioxide (TiO_2_) and molybdenum dioxide (MoO_2_); they stabilize the layers, enhance ion intercalation capability relevant to high-performance batteries and electrocatalytic systems, for which nitrogen doping and sulfur decoration promote further redox activity increase by increasing conductivity. Thus, modification with functional groups will take them to more specialized purposes, such as sodium-ion batteries and catalytic activity.

The hybrid materials such as MoS_2_ and WS_2_ were used for the enhancement of catalytic properties and corrosion resistance. These combinations allow making robust hybrid systems, especially in hydrogen evolution and storage technologies. The surface functionalization with fluorine, hydroxyl, and oxygen groups provides control of the MXene surface properties, optimizing their electrochemical performances for different energy applications.

Finally, 3D architectures represent a breakthrough in MXenes' structural frameworks. Based on templating and foaming techniques, these architectures improve volumetric performance and ion transport due to the critical challenges of compact energy storage devices. The graph accompanying this section depicts the distribution of research publications, pointing out the prominence of applications in batteries and supercapacitors while highlighting the growing interest in catalysis and hydrogen production.

**Fig. 23:**
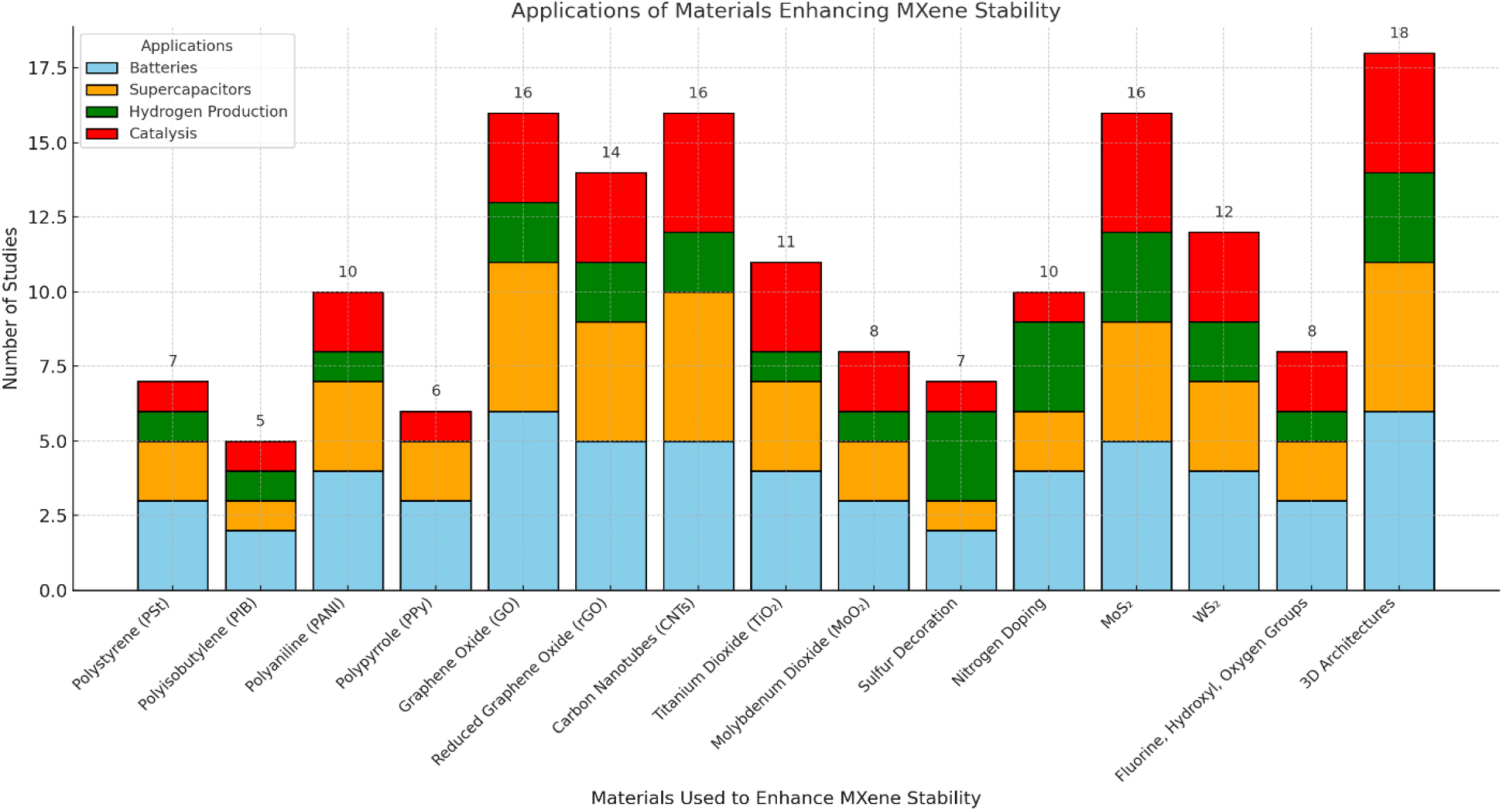
Distribution of research publications on materials enhancing MXene stability across energy applications: research trend analysis highlighting the distribution of studies focused on various materials used to enhance MXene stability across key energy-related applications—batteries, supercapacitors, hydrogen production, and catalysis.

The integration of these protective materials into MXenes illustrates their flexibility for the advancement of energy technologies through various means, as outlined in Table 5 and further supported by research trends represented in (Fig. 23). These advances create possibilities for sustainable and high-performance energy storage and conversion systems.

Table 8 provides the thermal stability and structural parameters of modified MXenes.

**Table 8:** Thermal stability and structural parameters of modified MXenes

MXene system	Coating/dopant	Oxidation onset temp. (°C)	Interlayer spacing (nm)	Conductivity retention (%)	Stability (hours in air)	Reference
Ti_3_C_2_T_*x*_	Al_2_O_3_ coating	≈450	1.05	—	<24	263
Ti_3_C_2_T_*x*_/PSt	Polyimide	≈460	1.10	85	>200	278
Ti_3_C_2_T_*x*_	Polyaniline	≈380	1.12	90	>150	268
Ti_3_C_2_T_*x*_/GO	Graphene oxide	≈500	1.08	95	>300	252
Ti_3_C_2_T_*x*_/CNT	Carbon nanotubes	≈420	1.03	92	>250	255
N-doped Ti_3_C_2_T_*x*_	Nitrogen doping	≈380	1.01	88	>100	145

The chart (Fig. 23), illustrates how polymers (*e.g.*, PSt, PANI), carbon-based materials (*e.g.*, CNTs, GO, rGO), metal oxides (*e.g.*, TiO_2_, MoO_2_), hybrid materials (*e.g.*, MoS_2_, WS_2_), functional groups (*e.g.*, –F, –OH, –O), and 3D architectures are strategically chosen to address MXene challenges like oxidation, restacking, and mechanical instability. These material integrations drive performance improvements in electrochemical systems, supporting the advances discussed in (Table 9) and throughout the text.

**Table 9:** Protective materials for enhancing MXene stability, properties, and applications with references

Protective material	Type	Function/role	References
Polystyrene (PSt)	Polymer	Prevents oxidation and maintains MXene conductivity over time	315
Polyisobutylene (PIB)	Polymer	Forms a protective coating to reduce conductivity degradation during long-term storage	316
Polyaniline (PANI)	Conductive polymer	Enhances mechanical stability and electrical conductivity	317
Polypyrrole (PPy)	Conductive polymer	Encapsulates MXenes, improving durability and electrochemical performance	318
Graphene oxide (GO)	Carbon-based material	Prevents layer restacking and improves structural stability	319
Reduced graphene oxide (rGO)	Carbon-based material	Enhances ion accessibility and provides structural support	320
Carbon nanotubes (CNTs)	Carbon-based material	Act as spacers to mitigate self-stacking of MXene layers	321
Titanium dioxide (TiO_2_)	Metal oxide	Stabilizes MXene layers and improves ion intercalation properties	322
Molybdenum dioxide (MoO_2_)	Metal oxide	Enhances chemical stability and electrochemical performance	323
Sulfur decoration	Functional group	Improves redox activity and ionic conductivity for battery applications	324
Nitrogen doping	Functional group	Boosts redox performance and conductivity	325
MoS_2_	Hybrid material	Enhances catalytic and corrosion-resistant properties	326
WS_2_	Hybrid material	Improves chemical stability and electrochemical properties	327
Fluorine, hydroxyl, oxygen groups	Surface functionalization	Controls surface properties to optimize electrochemical performance	163
3D architectures	Structural framework	Improves volumetric performance and ion transport through templating and foaming techniques	328

### 5.1. Scalability, economic feasibility, and safety considerations in MXene production

Despite their remarkable laboratory-scale success, most MXene synthesis strategies remain limited by issues of scalability, environmental compatibility, and process safety. A realistic industrial assessment must account not only for chemical yield but also for cost per kilogram, waste management, and occupational exposure risk.

#### 5.5.1. Economic feasibility

HF-based etching remains the most cost-effective route at the bench scale due to inexpensive reagents and moderate energy inputs. However, industrial analyses estimate a production cost of approximately 1200–1500$ per kg of Ti_3_C_2_T_*x*_ MXene, primarily due to acid neutralization and hazardous waste management. Electrochemical and alkali-assisted hydrothermal routes—though requiring specialized equipment—show potential for cost reduction to 900$ per kg through reagent recycling and continuous-flow processing. Molten-salt techniques, while producing high-purity materials, entail higher energy demands (>550 °C) and thus reach 1800$ per kg at pilot scale.

#### 5.5.2. Recycling and waste mitigation

Closed-loop HF recovery systems, employing distillation and CaF_2_ precipitation, can achieve >85% acid recycling efficiency, lowering both cost and environmental burden. Similarly, alkaline neutralization (*e.g.*, Ca(OH)_2_ treatment) converts fluoride waste into inert CaF_2_ sludge suitable for reuse in cement manufacture. Adoption of such recycling schemes is essential for sustainable scaling.

#### 5.5.3. Occupational safety

Direct handling of HF entails significant risk (dermal absorption, systemic toxicity). Regulatory limits set by OSHA (Occupational Safety and Health Administration) (3 ppm TWA) necessitate enclosed reaction systems and full PPE.^329^ Replacing concentrated HF with *in situ* generated HF (LiF/HCl, NH_4_F/HCl) or fluoride-free media (NaOH, algae extracts) significantly reduces personnel risk and simplifies effluent treatment.

#### 5.5.4. Scalability outlook

Among emerging methods, electrochemical etching and alkali-hydrothermal routes demonstrate the most industrial promise combining moderate cost, low toxicity, and scalability to multi-gram batches. Bio-assisted and UV-induced methods, while inherently safe, remain early in development but could become viable with process intensification.^76,330,331^

Overall, scalable MXene production will hinge on integrating reagent recyclability, waste valorization, and process automation to minimize cost and environmental footprint. The present assessment provides a first quantitative framework to evaluate these parameters, bridging the gap between academic synthesis and industrial implementation.

## 6. Challenges and innovations in MXene-based electrode materials for rechargeable batteries and supercapacitors

Despite their promising nature, MXenes do come with their challenges, to which innovative strategies are developed to address them, but all with their setbacks. The review targets key challenges and their corresponding innovations of MXene-based electrode materials for rechargeable batteries and supercapacitors-the most crucial challenges that face MXenes applications.

### 6.1. Oxidation sensitivity

In this regard, oxidation of MXenes, particularly in an atmosphere rich in oxygen or in the case of high humidity, degrades these nanomaterials very efficiently. Such degradation includes the loss of metallic conductivity and structural integrity and thereby seriously degrades the performances of the energy storage systems based on MXenes. While addressing this problem, several innovations were discovered by the researchers. First, protective coatings: thin coatings of polymers, metal oxides, or graphene provide a physical barrier against oxygen and moisture, preserving MXene properties over time. For instance, Ti_3_C_2_T_*x*_ showed enhanced stability upon polymer-based encapsulation. Although stability was attained, in most of the applications, coatings add dead weight and thickness to the electrode, which may reduce energy density. It is difficult to realize a uniform and scalable coating; this increases production costs. Second, inert conditions of storage: the storage of MXenes in atmospheres without oxygen–argon or vacuum-sealed environments-minimizes the exposure of materials to reactive agents. This method is pretty costly and consists of many complications in logistics, especially in large-scale production and long-term storage.^332–334^

Third, functionalization strategies: surface modification using the help of reducing agents or using –OH and –F type stabilizing functional groups will prevent oxidation by self-passivating the reactive sites on the surface of MXene. In this regard, functionalization may change the inherent electrochemical properties of the MXenes, for instance, reduction in conductivity or compatibility with a particular electrolyte.^316^

### 6.2. Restacking prevention

Owing to strong van der Waals forces, MXene nanosheets tend to restack, hence reducing their accessible surface area and ion transport channels, further worsening the performances of these materials in applications such as batteries and supercapacitors. The scientists tried to overcome these problems with a few innovations: first, the intercalation of nanoparticles.^335^

The principle relies on the fact that incorporation of nanoparticles, such as SiO_2_ or ZnO, between the layers of MXene acts like spacers, keeping the structure apart and allowing improved ion diffusion. One of the weaknesses of this invention is that the nanoparticles increase electrode weight and complicate the fabrication process of the electrode, hence affecting energy density and scalability.^336^

Second, polymer integration: integration of the MXenes with such polymers as polyvinyl alcohol (PVA) or polyaniline (PANI) results in the development of porous, flexible composites that enhance ion transport and mechanical stability. The weakness of this idea is that, generally, polymers possess relatively poor conductivity compared to MXene, which may weaken overall electrode performance.^337^

Additionally, some polymers degrade over time in certain electrolytes, compromising long-term stability.^337^

Third, 3D architectures developing three-dimensional structures, such as MXene aerogels or foam-like frameworks, allows for much better ion accessibility and avoidance of restacking. The drawback of this solution is that the creation of 3D architectures is resource-intensive, involving advanced fabrication techniques that may not be viable industrially,^336,338^ such structures may also introduce mechanical weaknesses, reducing durability during cycling.

### 6.3. Scalability

In addition, several big issues related to cost, environmental safety, and consistency in material quality are linked to the synthesis of MXenes on an industrial scale. For instance, the use of hydrofluoric acid (HF) in traditional methods has some risks regarding safety and the environment. Fluoride-free approaches include molten salt etching or acid-free chemical ways, which are safer and more eco-friendly. However, in these methods, MXenes mostly possess variable properties, for instance, inhomogeneous surface terminations.^93,339^

They are also not so efficient and require further optimization to reach the output of the HF-based processes. Some methods involving LiF and HCl have been developed for the delamination of MXenes, for example, Minimally Intensive Layer Delamination, reducing HF consumption in MXenes with fewer defects and larger lateral sizes. Though much safer, it is a process that deals with acidic reagents whose treatment and disposal should be carefully controlled; it could be a more time-consuming process and therefore difficult to scale. Continuous-flow systems are being developed for etching and delamination to reduce difficulties in large-scale MXene production. Automatization requires huge upfront investments in capital and technical know-how, thus remaining unreachable by smaller-scale producers.^340^

Consistency in the quality of the final product remains a concern. Recycling of reagents and abundant precursors reduce production costs and environmental impact. Recycling processes may introduce impurities into the final product, affecting the quality of MXene. Less pure precursors also compromise performance.^341^

The performance of MXenes products is highly appreciated but scientists should take in considerations these challenges to fix them in the future.

## 7. Challenges and innovations in MXene-enhanced photocatalysis for hydrogen production

Especially, MXenes, like Ti_3_C_2_, have demonstrated great potential in photocatalytic hydrogen evolution reactions due to their outstanding conductivity, tunable surface functionalities, and robust structure. Nevertheless, their real applications face enormous barriers, and innovative strategies need to be adopted to get through these challenges. In this section, the crucial challenges and relevant optimization approaches will be thoroughly discussed with reference to literature reports provided by Cheng *et al.* (2020) and Ran *et al.* (2016).^334,342^

### 7.1. Rapid electron–hole recombination

One of the major limitations of photocatalysis is the fast recombination of photoinduced electron–hole pairs, which seriously reduces the efficiency of hydrogen production. For instance, the hydrogen evolution rate of the bare CdLa_2_S_4_ photocatalyst was only 832.0 µmol g^−1^ h^−1^, which means that there were serious inefficiencies. To offset this, the addition of Ti_3_C_2_ as a co-catalyst was used with its high conductivity for good charge separation. Indeed, hydrogen evolution when incorporating Ti_3_C_2_ amazingly increased to 11 182.4 µmol g^−1^ h^−1^. Ran *et al.* (2016)^334^ further emphasized that MXene, in general, significantly promotes charge transfer and retards recombination, which could not be done without having them in HER.

**Fig. 24:**
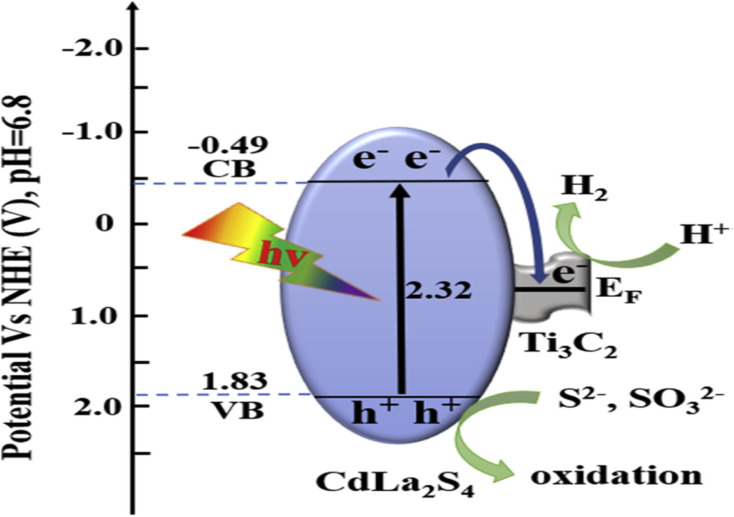
Mechanism of enhanced charge separation and hydrogen evolution in CdLa_2_S_4_/Ti_3_C_2_ composite photocatalysts: upon light irradiation (*hν*), photoinduced electrons in the conduction band (CB) and holes in the valence band (VB) of CdLa_2_S_4_ are generated. Without Ti_3_C_2_, rapid recombination of these charge carriers limits the hydrogen evolution rate. The introduction of Ti_3_C_2_ MXene serves as a conductive co-catalyst that facilitates electron transfer due to its high conductivity and favorable Fermi level (*E*_F), enabling electrons to reduce protons (H^+^) into hydrogen gas (H_2_). Meanwhile, holes are consumed by sacrificial agents (*e.g.*, S^2−^, SO_3_^2−^), further suppressing recombination. This strategy enhances photocatalytic performance significantly, increasing the H_2_ evolution rate from 832.0 to 11 182.4 µmol g^−1^ h^−1^. Reproduced with permission from ref. 342, Copyright 2020, Elsevier.

As illustrated in (Fig. 24) (Ran *et al.*,^334^ 2016), the mechanism of how MXene Ti_3_C_2_ can enhance the photocatalytic hydrogen production of CdLa_2_S_4_ to solve the critical problem of rapid electron–hole recombination. In the absence of the Ti_3_C_2_, the photoinduced electrons in the CB and holes in the VB of CdLa_2_S_4_ will result in fast recombination, limiting the hydrogen evolution rate to only 832.0 µmol g^−1^ h^−1^. This limitation is significantly minimized upon the introduction of other co-catalysts called Ti_3_C_2_s. It can be re-illustrated from here that Ti_3_C_2_ acts as an electron receptacle with high electrical properties and a favorable Fermil level, *E*_F_ because photogenerated electrons in Cd La_2_S_4_ undergo efficient transfer to the vicinity of the surface of thusly introduced Ti_3_C_2_, where they were involved in hydrogen evolution; that is, reacting back with H^+^ available to form H_2_ gas. This efficient charge separation suppresses the recombination of electrons and holes, enhancing the hydrogen evolution rate to 11 182.4 µmol g^−1^ h^−1^. Besides, as is shown in this image, the sacrificial agents consume the holes in the VB of CdLa_2_S_4_, further inhibiting the recombination process to ensure continuous photocatalytic activity. The mechanism described here reveals the transformation that Ti_3_C_2_ provides in enhancing charge carrier dynamics toward superior hydrogen production efficiency. Quantitative photophysics further corroborates this mechanism. In CdS/Ti_3_C_2_, steady-state PL (∼560 nm) is strongly quenched after MXene loading, while time-resolved PL lifetimes lengthen from an intensity-averaged *τ* ≈ 3.98 ns for bare CdS to ≈4.68 ns at 2.5 wt% Ti_3_C_2_ (components: *τ*_1_ = 0.61 → 1.54 ns; *τ*_2_ = 4.64 → 5.60 ns), evidencing suppressed recombination and more efficient electron extraction. Concomitantly, the interfacial charge-transfer resistance drops from ∼100.6 kΩ to ∼25.6 kΩ and the transient photocurrent rises, consistent with improved separation and transport.

The same trend is observed in other chalcogenide/MXene systems; for example, ZnIn_2_S_4_/Ti_3_C_2_O_*x*_ shows *τ*_avg increasing from ∼0.167 ns (pristine) to ∼0.594 ns with Ti_3_C_2_O_*x*_, along with enhanced photocurrent and a smaller EIS arc, indicative of a Schottky junction that retards recombination.

### 7.2. Content optimization of Ti_3_C_2_

The catalytic efficiency of MXene-based systems strongly depends on the precise optimization of the content of Ti_3_C_2_. Too low content leads to low efficiency in electron transfer, while high loading above 1.0 wt% creates recombination centers and hinders light absorption. Experimental results show that the optimum balance, which maximizes catalytic performance without the introduction of adverse effects, is reached with 1.0 wt% of Ti_3_C_2_. This agrees with the work done by Ran *et al.* (2016),^334^ who obtained an optimum loading that has to be maintained in order not to result in aggregation, with consequent inefficiencies.

**Fig. 25:**
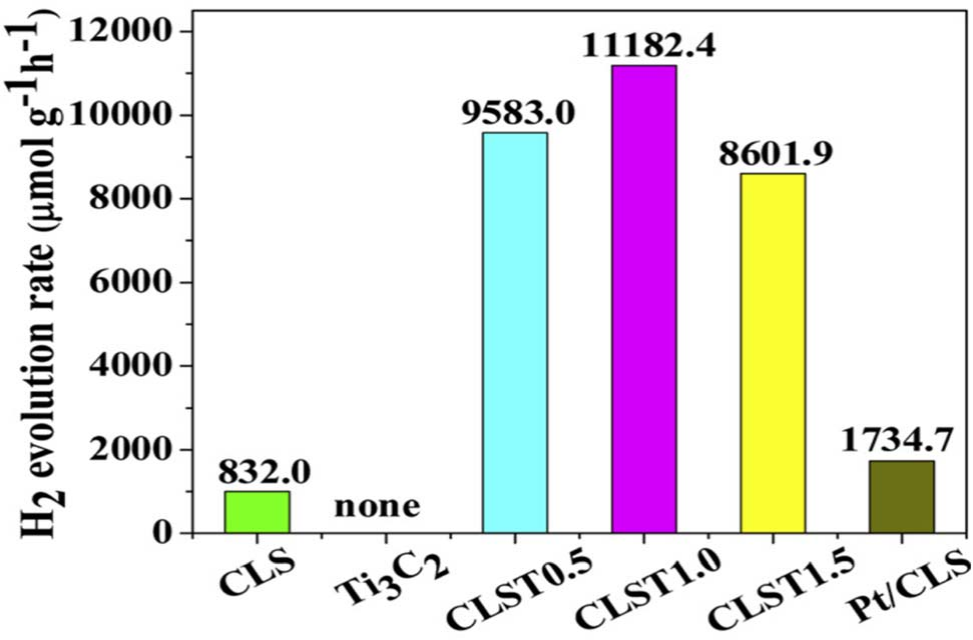
Optimization of Ti_3_C_2_ content for maximizing hydrogen evolution rates in CdLa_2_S_4_ photocatalysts: the graph shows the significant enhancement in hydrogen production upon introducing Ti_3_C_2_ as a co-catalyst. The bare CdLa_2_S_4_ (CLS) exhibits a low hydrogen evolution rate of 832.0 µmol g^−1^ h^−1^, which drastically increases to 11 182.4 µmol g^−1^ h^−1^ at the optimized Ti_3_C_2_ loading of 1.0 wt% (CLST1.0). Loadings below or above this optimum (*e.g.*, 0.5 wt% and 1.5 wt%) result in lower performance, highlighting the importance of precise content tuning to avoid inefficient electron transfer or excess aggregation that can suppress light absorption and increase recombination centers. Reproduced with permission from ref. 342, Copyright 2020, Elsevier.

As shown in (Fig. 25) (Cheng *et al.*,^342^ 2020), hydrogen evolution rates of photocatalysts CLS with various contents of loaded Ti_3_C_2_, showing the role of optimization in deciding catalytic efficiency. Bare CdLa_2_S_4_ catalysts possess very low intrinsic activity, amounting to only 832.0 µmol g^−1^ h^−1^. The addition of the co-catalyst Ti_3_C_2_ significantly enhances the hydrogen evolution, which further supports the important role that it plays in improving electron transfer and hindering recombination. At the optimum Ti_3_C_2_ loading, 1.0 wt% reaches to CLST1.0, showing a peak hydrogen evolution rate of 11 182.4 µmol g^−1^ h^−1^. This impressive improvement reflects a perfect balance that provides adequate electron transfer and minimizes the recombination centers. When further increasing the loading beyond 1.0 wt%, as represented by CLST1.5, it decreased the hydrogen evolution rate to 8601.9 µmol g^−1^ h^−1^, which might be due to the extra amount of Ti_3_C_2_, which induced aggregation to block light absorption and formed a recombination center, thereby reducing catalytic efficiency.

This figure is in good agreement with the discussion that optimization of the Ti_3_C_2_ content should be precise to obtain the maximum catalytic performance: too little Ti_3_C_2_ leads to poor electron transfer, while too much depresses light absorption and introduces inefficiency; thus, confirmation that keeping an optimum content at 1.0 wt% is necessary for the best catalytic outcome was obtained.

### 7.3. Sensitivity to oxidation

The MXenes have been highly susceptible to oxidation under both oxygen-rich and humid environments, leading to a serious loss of metallic conductivity along with structural integrity. Therefore, this degradation puts major limits on their long-term usability in HER applications. The other strategy involves the design of new solutions using polymer, graphene, or metal oxides protective coatings that protect MXenes from oxidative destruction and improve operational stability. Indeed, this has been demonstrated in several works, including that by Ran *et al.* in 2016. Functionalization with the likes of –OH and –F stabilizing groups further mitigates the oxidation, though with possible minor influences on conductivity and compatibility with certain sorts of electrolytes.

### 7.4. Restacking and surface accessibility

The strong van der Waal forces between MXene nanosheets induce restacking, reducing their active surface area and limiting pathways for ion and electron transportation. This is a key structural bottleneck that hampers the catalytic activities of these materials. The intercalation with nanoparticles, such as SiO_2_ or ZnO, and the preparation of three-dimensional architectures, like MXene aerogels or foam-like structures, are among other adopted strategies for solving this. This would avoid restacking and increase the active surface area, although generally involving more complicated synthesis processes.

### 7.5. Synthesis and homogeneous dispersion

Homogenous dispersion of photocatalysts such as CdLa_2_S_4_ onto the MXene surface remains a big challenge without sacrificing the two-dimensional structure. Cheng *et al.*^342^ developed a two-step synthesis protocol combining HF etching, ultrasonic exfoliation, and *in situ* growth of CdLa_2_S_4_ nanoparticles. This method effectively gave rise to a robust composite formation with preservation of structural integrity for Ti_3_C_2_, thus enhancing catalytic performance.

### 7.6. Stability and durability

Generally, the long-term stability of MXene-based systems is not ensured and eventually leads to photocorrosion and degradation in the case of repeated HER cycles. Long-term stability remains the most cardinal factor in their practical usage. The strong interfacial bonds between MXenes and photocatalysts have been effective in enhancing durability. For example, the structure and catalytic activity of the Ti_3_C_2_–CdLa_2_S_4_ system did not change after six recycling tests with very slight losses. Ran *et al.*^334^ (2016) also pointed out that hybridization and surface functionalization play a significant role in enhancing stability for long periods of operation.

### 7.7. Cost and scalability

Traditional HER systems rely on the use of expensive and hardly upscaled noble metals such as Pt. Similarly, there are some tough environmental and safety problems in the synthesis of MXenes by HF-based etching. The use of Ti_3_C_2_ as a co-catalyst has been an economic way out to achieve similar catalytic performances to those of noble metals. Besides, the development of fluoride-free or less intensive delamination methods improved scalability and safety. Continuous-flow systems and automation for large-scale production are under exploration, although further optimization is needed to ensure product consistency and reduce costs. This discussion which explained depending on the studies of Cheng *et al.*^342^ (2020) and Ran *et al.*^334^ (2016) indicates that MXenes, more importantly Ti_3_C_2_, have also shown very good promise as co-catalysts in the hydrogen evolution reaction for sustainable and scalable options besides the traditional concept of noble metals. There has been impressive overcoming challenges associated with electron–hole recombination, sensitivity of oxidation, and scaling-up of such photocatalysts to the advancement in hydrogen generation. But we cannot ignore putting emphasis on MXene materials as truly transformative photocatalysis able to open a way toward different sustainable energy technologies. While fluoride-based etching methods are effective, their reliance on hazardous chemicals poses risks, especially in biomedical applications where residual HF can cause cytotoxicity. Additionally, fluoride-terminated MXenes have a lower percentage of more functionally diverse groups like –OH and –O, which are better candidates for further chemical conjugation. Therefore, fluoride-free etching processes are thought to be more desirable to customize surface terminations, particularly for MXenes targeted towards biological or environmentally friendly applications.

## 8. Conclusion

Over the past decade, MXenes have proven to be among the most stimulating classes of two-dimensional materials owing to their exemplary physicochemical features like higher electrical conductivity, very large surface areas, mechanical stability, hydrophilicity, and controllable surface chemistry. In the present review article, structure, preparation procedures, and multimodal applications of MXenes have all been exhaustively explored with a particular emphasis placed on their applications towards energy storage and conversion, environmental protection, and biomedical purposes.

One of the most important insights is how the synthesis route of the MXenes—whether bottom-up or top-down—plays a key factor in dictating their structural stability, surface chemistry, and functionalization to a particular application. Top-down etch processes are currently more scalable and widespread but are accompanied by environmental and safety concerns as a result of the aggressive chemicals used. The bottom-up processes have better control of the material parameters, yield better quality MXenes but are constrained by price and complexity. Thus, the ultimate challenge of the future lies in balancing scalability, friendliness to the environment, and synthesis precision to enhance the production of the MXenes.

An important research directions is the precise control of surface terminations and chemical functionalization. Potentially, controlling the surface chemistry of MXenes could have dramatic impacts on their suitability for catalysis, sensing, and energy storage. This unifying principle of surface chemistry is critical for advancing the use of MXenes in real-world applications. For instance, the same surface groups that prevent oxidation in energy storage devices are also key to enhancing long-term stability in medical and environmental applications, where exposure to air and fluids is common. In addition, their incorporation into hybrid composites and scalability into architectural structures of devices will be required to utilize them in real applications. Advancement in such areas will bring MXenes from a research material to a prospective building block of next-generation technologies in the fields of energy, the environment, and medicine.

In the future, the highest attention has to be on the green and eco-friendly synthesis methods that avoid the excessive use of toxic reagents like hydrofluoric acid. The methods have to introduce safe as well as sustainable routes to MXene fabrication without affecting the quality of the material. Most importantly, enhancing their long-term stability as well as their resistance against oxidation, especially in applications like medicine and the environment, where exposure to air, water vapor, or biological fluids has a drastic impact on their functionability, is a necessity.

Looking forward, the next phase of MXene research will require convergence between synthetic precision, environmental safety, and device integration. The field is now moving from laboratory-scale etching protocols toward continuous-flow, fluorine-free, and hybridized production routes that minimize cost and environmental burden while maintaining high electronic quality. Equally, bridging data-driven modeling with real-world performance metrics will enable predictive design of MXenes tailored for specific end uses ranging from high-rate supercapacitors to biodegradable biomedical coatings. Our review provides a consolidated foundation for this transition, identifying unresolved challenges and offering a forward-looking perspective that extends beyond current literature to guide both fundamental inquiry and applied innovation.

In summary, this work extends beyond prior syntheses by constructing a multidimensional, data-driven view of MXene research. Unlike earlier reviews that treated energy, environmental, and biomedical uses as parallel topics, our analysis interlinks these domains through common synthesis–structure–function pathways. The quantitative benchmarking tables and graphical comparisons provide, for the first time, a normalized view of etching efficiency, conductivity, surface area, and scalability across diverse MXene systems. This integrative and comparative approach positions the current review as both a continuation and a conceptual advancement of the MXene literature.

## Conflicts of interest

Authors declare no competing interest.

## Data Availability

No primary research results, software or code have been included and no new data were generated as part of this review.
